# An Automated Image Analysis System to Quantify Endosomal Tubulation

**DOI:** 10.1371/journal.pone.0168294

**Published:** 2016-12-22

**Authors:** Timothy M. Newton, Evan Reid

**Affiliations:** Department of Medical Genetics and Cambridge Institute for Medical Research, University of Cambridge, Cambridge, United Kingdom; "INSERM", FRANCE

## Abstract

Recycling of cargos from early endosomes requires regulation of endosomal tubule formation and fission. This regulation is disrupted in cells depleted of the microtubule severing enzyme spastin, causing elongation of endosomal tubules and mis-trafficking of recycling endosomal cargos such as the transferrin receptor. Spastin is encoded by SPAST, mutations in which are the most frequent cause of autosomal dominant hereditary spastic paraplegia, a condition characterised by a progressive loss of lower limb function resulting from upper motor neuron axonopathy. Investigation of molecular factors involved in endosomal tubule regulation is hindered by the need for manual counting of endosomal tubules. We report here the development of an open source automated system for the quantification of endosomal tubules, using ImageJ and R. We validate the method in cells depleted of spastin and its binding partner IST1. The additional speed and reproducibility of this system compared with manual counting makes feasible screens of candidates to further understand the mechanisms of endosomal tubule formation and fission.

## Introduction

### Endosomal tubulation in hereditary spastic paraplegia (HSP)

Endocytosed membrane proteins that are destined for recycling, rather than degradation in the lysosome, are sorted away from the endosomal compartment via several different recycling pathways. In general, a first step in recycling involves the formation and fission of endosomal tubules from the endosomal body. Some endosomal membrane protein cargoes are selectively recruited into these membrane tubules whilst others are transported by bulk flow, with the large surface area to volume ratio of tubules helping to provide selectivity for these cargoes [1, 2]. Formation of endosomal tubules involves the sorting nexin (SNX) proteins, which bind the cytosolic face of endosomal membrane via an arched BAR (Bin, amphiphysin, Rvs) domain, which preferentially associates with highly curved membranes [3]. Different recycling pathways are preferentially marked by specific SNX proteins, e.g. SNX1 marks the retromer pathway that recycles receptors to the Golgi, while SNX4 marks a pathway involved in recycling to the plasma membrane [4, 5].

Mechanisms of endosomal tubule fission are emerging. We proposed recently that the microtubule severing enzyme spastin is involved in this process [6]. Spastin is recruited to the ESCRT (endosomal sorting complexes required for transport) III complex at endosomes by binding to the ESCRT-III complex-associated proteins IST1 and CHMP1B [7–9], and depletion of either spastin or IST1 in tissue culture cells results in an increased number of long endosomal tubules, including those marked by SNX1 and SNX4 [6].

Spastin is encoded by the SPAST gene, which is mutated in approximately 30–40% of autosomal dominant HSP families [10]. HSP is clinically characterised by progressive weakness and spasticity in the lower limbs, resulting from distal axonal degeneration in the corticospinal tract upper motor neurons [11]. The pathological relevance of endosomal tubulation in this axonopathy is suggested by the presence of endosomal tubules in Zebrafish axons depleted of spastin [6]. Thus, as well as elucidating a process of basic cell biological importance, identifying proteins that can modulate endosomal tubulation may reveal mechanisms involved in HSP and axonal maintenance.

### Manual analysis of endosomal tubulation is a rate limiting factor

In our work on the role of spastin in endosomal tubulation we have quantified the phenotype by manually counting the number of SNX1 positive tubules in images taken by a widefield fluorescent microscope or by reporting the percentage of cells with at least one long tubule. This manual counting process is time consuming and a rate-limiting step in investigating endosomal tubulation phenotypes. The laborious nature of this process limits the type of future work that can be conducted, such as assessing a wider variety of genetic knockdowns that may lead to tubulation, or conducting rescue experiments to determine sequence-function correlations.

To overcome these difficulties, we have developed an automated tubule counting system that analyses manually recorded images from a widefield fluorescent microscope. The accuracy of this system was validated versus manual counting. The speed and accuracy of analysis opens the possibility of higher throughput tubule analysis, including small, targeted screens of genes and proteins of interest.

## Materials and Methods

### Cell culture conditions

HeLa-M cells were obtained from the Lehner lab, Cambridge Institute for Medical Research and MRC5 fibroblasts were obtained from the Morrell lab, Department of Medicine, University of Cambridge. Cell were cultured in Dulbecco's Modified Eagle's Medium (DMEM) 6456 (Sigma) supplemented with 10% (v/v) foetal calf serum (FCS), 1% Penicillin/ Streptomycin and 2 mM L-Glutamine at 37°C and 5% CO2 in a humidified incubator.

### siRNA and DNA transfections

For siRNA transfection, cells were transfected in six well plates with 5 μl Oligofectamine (Invitrogen) per well in antibiotic-free media. Transfections were carried out one day after cells were plated at cell densities stated in specific experimental descriptions. siRNA (Dharmacon) was used at 10 nM final concentration per gene targeted. Depletion of proteins following siRNA transfection was verified by western blotting using rabbit polyclonal anti-spastin 86–340 (generated in house [12]) or rabbit polyclonal anti-IST1 (Proteintech 51002-1-AP).

### Immunoflourescence and image collection

HeLa cells were plated onto coverslips in 6-well plates (Gibco) at a density of 20,000 cells per well and transfected with siRNA after 24 hours. Cells were incubated for a further 120 hours after transfection before fixation with 4% formaldehyde. Cells were labelled for SNX1 with 1:200 α-SNX1 (611482—BD Transduction Laboratories) antibody and Alexafluor 488 (Thermo Fisher Scientific) secondary antibody as described previously [12], with an additional 30 minute incubation in 1:10,000 whole cell stain (HCS cell mask red, Thermo Fisher Scientific) before imaging on an AxioImager Z2 widefield fluorescent microscope. 1024 x 1024 images were taken with a 63x lens, with 20 ms exposure for the red channel (cell mask) and 400–800 ms exposure for the SNX1. Exposure times for these markers were constant in individual experimental biological repeats, but were adjusted between experiments so that the imaged pixels were as bright as possible without becoming saturated.

### Datasets

Independent datasets were used for developing and testing the automated counting system. The HeLa cell ‘test’ dataset contained images taken from three independent biological repeats. The MRC5 cell dataset corresponds a single biological experiment, with three technical repeats.

### Method overview

ImageJ and R scripts (S1 and S2 Appendix), a user guide (S3 Appendix) and example images/results (S1–S3 Datasets) can be found in supporting materials. A brief description of the method is given below:

#### ImageJ macro

**Fig 1:**
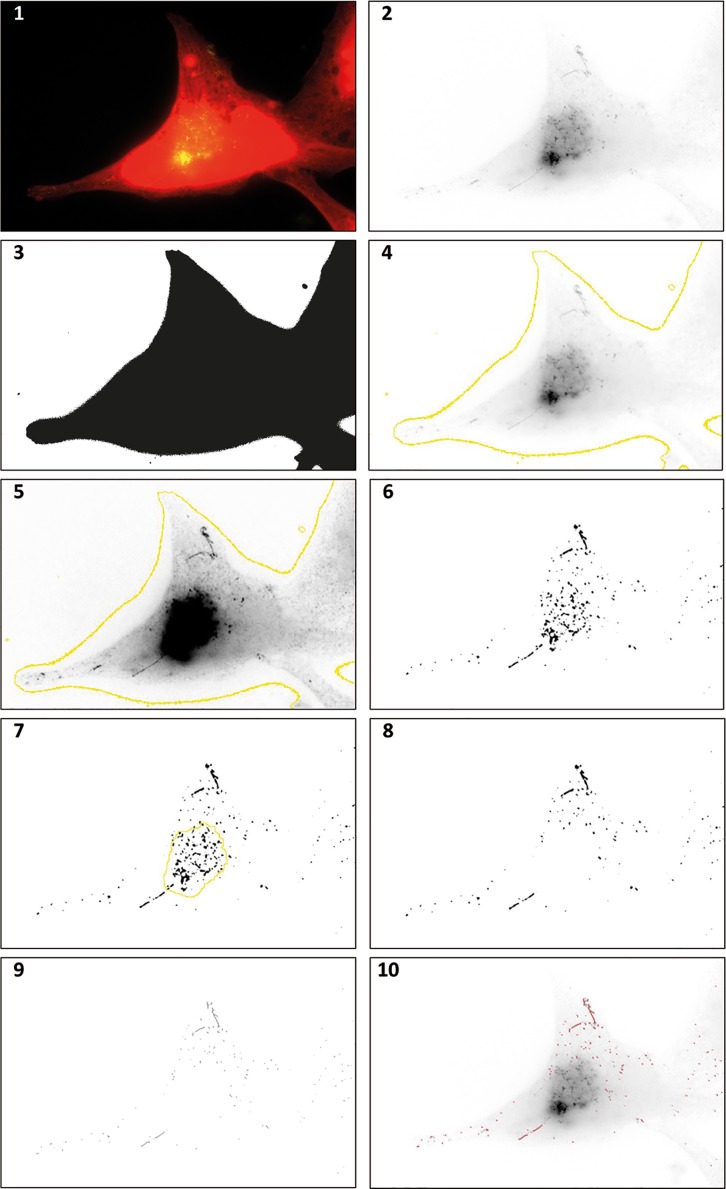
ImageJ processing of endosomal tubulation images. Panel 1: HeLa cell stained with whole cell stain (red) and snx1 (green). Panel 2: Green channel only. Note the presence of endosomal tubules. Panel 3: Red channel only, used as a mask to exclude areas in the image that are not part of a cell from image analysis. Panel 4: Overlaid boundary from whole cell stain on the green channel. Panel 5: Green channel after image manipulation. Background subtraction, smoothing, contrast enhancement and noise removal have been carried out. These manipulations are necessary to facilitate successful representation of tubules in the next step. Panel 6: A binary image taken from image 5 for purposes of comparison. The microtubule organising centre (MTOC) can be seen as a dense area of Snx1 staining, which greatly increased noise in image analysis. Panel 7: The identification of the MTOC (yellow) allows its removal before an image is made binary. Panel 8: A binary image made after the MTOC has been removed. Panel 9: A ‘skeletonised’ image processed from the binary image. Tubules can then be identified with the ImageJ plugin “analyse skeleton”. Panel 10: Identified tubules (purple) overlaid on the original green only image from panel 2.

Recognition of endosomal tubules labelled by SNX1 was carried out by ImageJ, with the ‘Tubule recognition’ macro (supplementary materials). An overview of the steps included in the Tubule recognition macro is given in Fig 1. This macro enhances SNX1 signal, removes noise and ‘skeletonises’ areas of signal to 1-pixel width lines. During development of the macro we found that the dense and overlapping clustering of SNX1 puncta at the microtubule organising centre (MTOC) led to frequent mis-identification and over-calling of tubules in this region, leading to poor signal to noise discrimination. We addressed this by introducing steps to identify the MTOC and remove it from analysis (Fig 1, steps 6–8). After skeletonisation, the macro then utilises the ‘analyse skeleton’ plug-in which identifies and measures each skeletonised line in an image and records the data in a.csv file for every image analysed [13].

#### Data analysis

Data produced by ImageJ was processed within the statistical programming environment R. Tubules were classified as linear or curvilinear structures over 20 pixels in length, which corresponds to 2μm, the minimum length of tubules we previously used as a cut-off to successfully identify tubulation phenotypes in manual counting approaches [6]. Three methods for analysing data were used: Mean number of tubules per image, the percentage of images with at least one identified tubule and mean length by which the longest tubule measured exceeded 2μm, (i.e. 20 pixels). The latter metric identifies the longest of the tubules that exceed the 2μm threshold in each image, measures its length, calculates the length by which it exceeds 2μm, then generates a mean value for this 4ure over all images analysed. As well as giving an indication of the effect of an experimental manipulation on tubule length, when considered with the tubule numbers parameter, this metric helps identify phenotypes resulting in fewer but longer tubules, which may be underrepresented if only tubule number is taken into account. We used this metric rather than simply longest tubule length, as it is easier to visually discriminate differences in the corresponding histograms. To extract these three sets of data the R script ‘Tubule Analysis’ was used (S2 Appendix). Data is exported from R in a.txt file listing the file name along with the three metrics given above.

#### Z’ calculation

Z’ scores were calculated with the following formula:

$$1-\frac{3*(\sigma^{p}+\sigma^{n})}{{\bar{x}}^{p}+{\bar{x}}^{n}}$$

In which *σ*^*p*^ represents the standard deviation of the positive control, *σ*^*n*^ represents the standard deviation of the negative control, ${\bar{x}}^{p}$ represents the mean of the positive control and ${\bar{x}}^{n}$ represents the mean of the negative control.

## Results

### Training data

**Fig 2:**
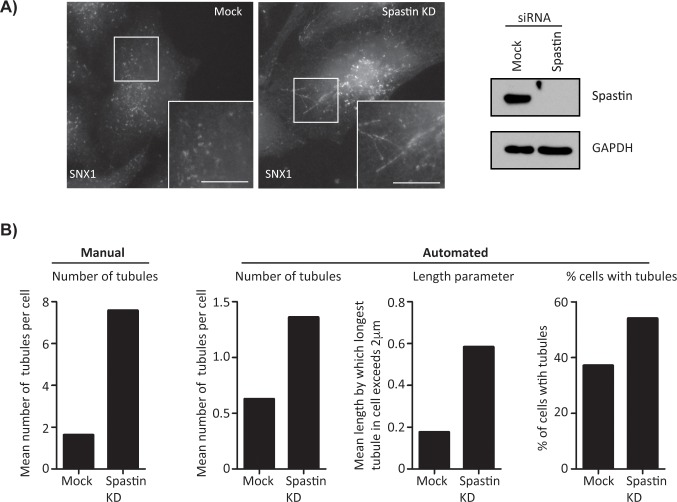
System development. A) Endosomal tubulation in mock transfected and spastin depleted cells. In mock transfected cells (left panel) the endosomal resident protein SNX1 marks an endosomal subdomain and short endosomal tubules. These elongate in cells treated with spastin siRNA (right panel). Scale bar = 10μm. B) Results of automated tubule analysis with data used to develop the system. Increases in endosomal tubulation are observed in all three analysis types. The same data, manually counted, is shown for comparison. True mean length of longest tubule per image can be calculated by adding 2μm to the values shown in the length parameter histogram. 30 images were taken per condition, n = 1.

We began by using the automated counting system (see Methods) with a ‘training’ dataset consisting of 30 images taken from control cells or cells depleted of spastin by siRNA transfection. Analysis of this dataset identified increased endosomal tubulation in cells lacking spastin with all three categories of analysis; percentage of cells with tubules, mean number of tubules per cell and the mean length by which the longest tubule in an image exceeded the 2μm threshold (Fig 2). The automatically counted tubule number results showed a similar proportional increase versus manually counted data (Fig 2B), although a fewer absolute number of tubules was detected with the automated counting system.

### Test data

**Fig 3:**
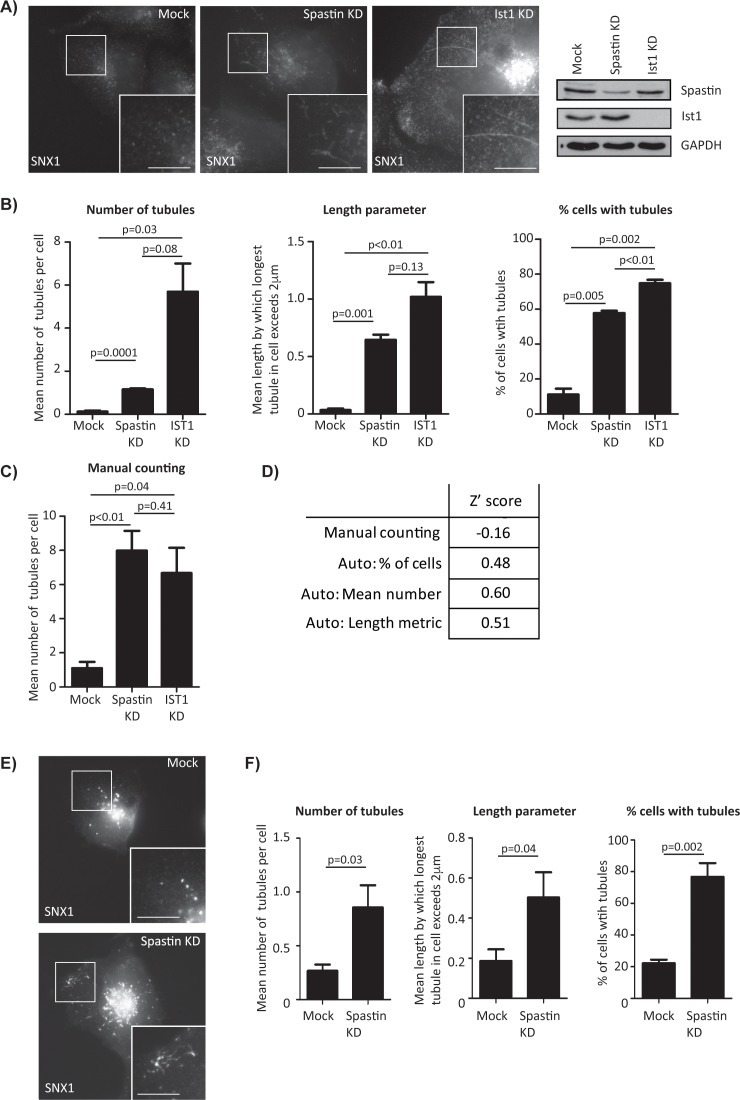
Verification of automated tubule counting. A) SNX1 signal in spastin and IST1 depleted cells. Scale bar = 10μm. B) Results from automated tubule counting on a data set not used in training. This dataset contained 3 independent repeats, with 45 images per condition. C) Manually counted results from the same data set. D) Z’ scores between spastin depleted and mock cells. E) SNX1 signal in wild-type MRC5 and a cell depleted of spastin. F) Automated counting data generated from a spastin depletion experiment in MRC5 cells. In B) and C), results are shown as the mean of three experiments plus the standard error of the mean, in F) results are the mean of three technical repeats plus the standard error of the mean, with 60 images analysed per condition. For the length metric, true mean length of longest tubule can be calculated by adding 2μm to the values shown in the length parameter histogram. In B) and C) p values were calculated with paired t-tests, in F) with unpaired t-tests.

When creating automated image recognition systems, there is a risk of ‘over-training’ to a sample set of data. Rather than creating a system that recognises the phenotype of choice, over-training causes the system to distinguish between two specific sets of images, without being generally applicable to other data. To verify the automated counting system and ensure over-training had not occurred a new ‘test’ dataset of control cells or cells transfected with spastin siRNA was used. Images from cells depleted of IST1 were also included to verify the method as being applicable to a tubulation phenotype caused by depletion of an independent protein. Significant effects for cells depleted of spastin or IST1 versus controls were detected in all three categories of analysis (Fig 3A–3C). Of note, in each category of automated counting the size of the IST1 depletion effect was greater than the spastin depletion effect; this is not reflected in the corresponding manual counts.

Z’ scores are used to identify an assay’s suitability for high throughput screening; scores above 0.5 indicate a robust assay for screening, scores between 0 and 0.5 suggests the assay is functional, but marginal, and scores below 0 suggest the assay is not suitable for screening. Z’ scores for the auto-counting system suggest that mean tubule number and mean tubule length metric would provide robust screening methodologies, while the percentage of cells with tubules would provide a functional but marginal assay (Fig 3D).

To determine whether the automated counting methodology would be applicable to other cell types, we carried out spastin depletion experiments in MRC5 lung fibroblasts and imaged endogenous SNX1. Subsequent automated counting identified a clear effect on endosomal tubulation analysis parameters (Fig 3E and 3F).

The minimal dataset for results presented in Figs 1–3 is provided in S4 Dataset.

## Discussion

We have developed an automated counting system for quantifying endosomal tubulation using as an example depletion of two proteins, spastin and IST1, which we have proposed to drive endosomal tubule fission [6]. The automated counting system produced results with speed, reproducibility and specificity. The system was much less labour intensive than manual counting (we estimate a factor of approximately 30 versus manual counting of a 600 image experiment) and the increase in analysis speed allows more images to be analysed per condition, further improving accuracy.

Multiple parameters could be analysed at the same time with automated counting, and this highlighted possible differences between the spastin and IST1 phenotypes that were not obvious from manual counting of the number of tubules per cell; for example the mean length of the longest tubule metric showed a trend towards being greater in cells depleted of IST1. In addition, while the absolute number of tubules counted in IST1 depleted cells was broadly comparable between automated and manual results, the number of tubules detected by automated counting in spastin depleted cells was fewer than in manual counting. These differences almost certainly relate to an interplay between different biological characteristics of the tubules, and choices made in the design of the autocounting system; tubules in IST1 depleted cells are generally straighter and longer than those in spastin depleted cells (a representative example is shown in Fig 3A), and the autocounting design prioritised specificity of tubule detection over sensitivity, using length as a factor in making this decision. Thus shorter tubules (as seen in spastin depletion) are under-counted by the automated counting system versus manual counting. Nevertheless, the system was able to robustly detect both spastin and IST1 tubulation phenotypes.

There are several caveats and practical points to note regarding use of our system. A) We have only tested the system on relatively flat (HeLa) or very flat (MRC5) adherent cell lines, and so the system may not perform as well for cell lines that do not have these characteristics. B) The images we analysed were collected with epiflourescence using a high quality wide field microscope and imaging system, and the automated counting system should be validated and optimised before use with other microscopy approaches, for example Z-stacks generated by confocal microscopy. The analysis is also dependent upon the input of high quality in-focus images into the system and for this reason we collected images manually. The automated counting system may be applicable to images generated from an automated microscope, provided that images of sufficient quality could be produced. The better performance of high-quality images may be reflected in the modestly improved discrimination of the effect of spastin depletion in the test dataset (Fig 3B) versus the training set (Fig 2B) set; cells from the training dataset were imaged several times during development of the system, resulting in some loss of fluorescence signal and less optimal signal to noise. C) The system is likely to undercount short tubules (see comments above regarding the differing performance of the automated system for quantifying numbers of tubules in spastin-depleted versus IST1-depleted cells). In a screen, incorporation of the mean longest tubule length metric and the percentage of cells with at least one tubule metric into the analysis will minimise the risk of a false negative result in this situation. D) We have so far only used automated counting to detect tubules marked by antibodies to SNX1. Theoretically the system could be used to analyse other tubular compartments, but would need to be optimised for other markers on a case by case basis, as each will have different sensitivities and specificities, resulting in different signal-to-noise ratios. E) During development of the automated counting system, we found that removal of the region of the MTOC from analysis reduced false-positive calling of tubules and improved discrimination of the effects of spastin or IST1 depletion on SNX1 tubulation. However, this manipulation means that experimental conditions that exclusively or even predominantly cause increased tubulation at the MTOC will not be identified by the system, which will likely deliver a false-negative in this situation. In addition, experimental manipulations that cause the marker under test to cluster at, or be dispersed from, the MTOC are likely to cause effects on tubule counts that are unrelated to the true extent of tubulation. Thus effects on the distribution of the marker should be taken into account in interpreting results.

Despite these caveats, the creation of a semi-automated system for counting endosomal tubules greatly reduces the time taken to analyse endosomal tubulation and could be useful in many scenarios. The speed and accuracy of this system will make feasible small scale siRNA screens examining endosomal tubulation or perhaps larger scale screens if combined with an automated image capture system. These screens could identify other proteins required for tubule fission, or reveal other factors that can rescue the spastin-depleted phenotype, increasing our understanding of endosomal tubule fission and its role in diseases such as HSP.

## Supporting Information

S1 Appendix ImageJ script that recognises tubules in an Image. This macro must be modified by the user before use.
(TXT)

S2 Appendix R script that processes data produced by the ImageJ macro provided in S1 Appendix. This script must be modified by the user before use.
(TXT)

S3 Appendix User guide containing instructions for use of the auto counting system. This includes modifications the user must make to the ImageJ and R scripts (S1 and S2 Appendix) before analysing images.
(DOCX)

S1 Dataset Example images of mock-transfected cells. Whole cell stain in red channel, endogenous SNX1 labelled in green channel.
(ZIP)

S2 Dataset Example images of spastin-depleted cells. Whole cell stain in red channel, endogenous SNX1 labelled in green channel.
(ZIP)

S3 Dataset Example results. Results of automated counting using the S1 and S2 datasets.
(XLSX)

S4 Dataset Minimal dataset used in Figs 1–3. (XLSX)
