# Supplementary material for: An Automated Image Analysis System to Quantify Endosomal Tubulation
Source: PLoS One. 2016 Dec 22;11(12):e0168294. doi: 10.1371/journal.pone.0168294 (PMC5179261; doi:10.1371/journal.pone.0168294)
Supplement: S3 Appendix — This includes modifications the user must make to the ImageJ and R scripts (S1 and S2 Appendix) before analysing images. (DOCX) [file pone.0168294.s003.docx]

**User guide**

**Outline**

This user guide explains the steps necessary for counting endosomal tubules using ImageJ, R and Excel. In order to carry out this analysis the user must have ImageJ and R installed on their PC.

ImageJ can be downloaded here:

<https://imagej.nih.gov/ij/download.html>

R can be downloaded here:

<https://cran.r-project.org/bin/windows/base/>

The following steps are described in this userguide:

1. Image Collection

- *This section includes microscopy parameters used in image acquisition.*

1. Install ‘Analyse Skeleton’ ImageJ plug-in

- *This installs an additional feature to ImageJ required for tubule analysis.*

1. ImageJ macro customisation

- *The ImageJ macro produces.csv files which will be saved in a location determined by the user. This destination is included in the macro and must be manually modified before analysis.*

1. ImageJ run macro

- *The process of running ImageJ macros is described.*

1. R script customisation

- *The R script requires a ‘working directory’ This must match the destination folder for .csv files specified by the user in step 2*

1. Run R script

- *Running the ‘Tubules Analysis’ script in R is described.*

1. Excel

- *Final data processing to obtain average values per condition is carried out in R*

**1) Image Collection**

Images collected for analysis were taken with an AxioImager Z2 microscope. Other widefield fluorescent microscopes should be suitable, providing image quality is high. 1024 x 1024 images were taken with a 63x lens, with a final scale per pixel of 103 μm x 103 μm. Exposure was set at 20 ms for the red channel (cell mask) and 400-800 ms exposure for the green channel (SNX1). Images should be stored as ‘.tiff’ files in a single folder.

**2) Obtain the ‘Analyse Skeleton’ plugin for ImageJ**

This tubule recognition system uses the ‘Analyse Skeleton’ ImageJ plugin (Arganda‐Carreras et al., 2010). Before commencing with image analysis this plug-in must be installed in ImageJ using instructions found [here](http://imagejdocu.tudor.lu/doku.php?id=plugin:analysis:analyzeskeleton:start).

**3) ImageJ macro modification**

The ImageJ macro ‘Tubule recognition’ produces a .csv file for every image analysed. Before running the ImageJ macro this save location must be specified by the user. To select which folder to save .csv files in open the file ‘Tubule Recognition.txt’ present in supplementary materials, and scroll to the bottom of the file:


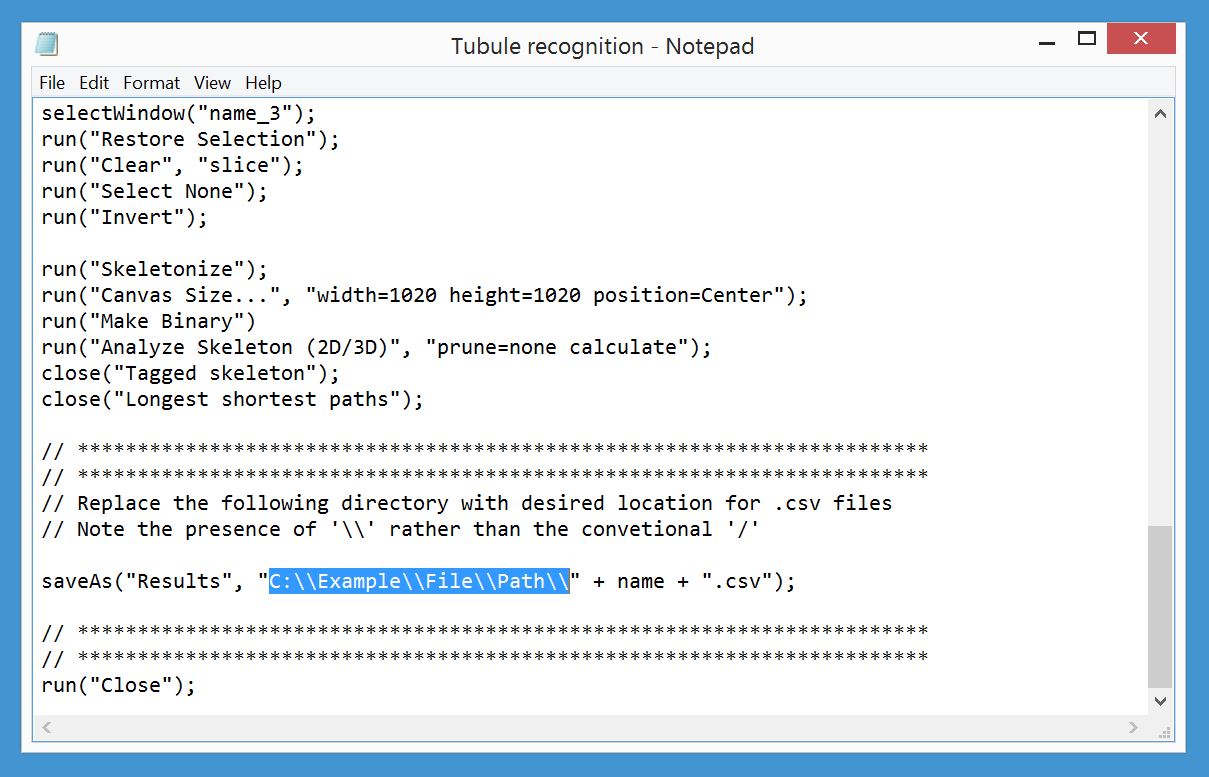


The highlighted segment in blue is the file path where the results of image analysis by ImageJ will be stored as .csv files. Change this highlighted segment to the desired location, noting the presence of ‘\\’ as a separator in the file-path rather than ‘/’. Also note the presence of the final ‘\\’ after the last level of the file-path. The location specified by the user should already exist as an empty folder before the ImageJ macro is used. After editing this macro it should be saved ready for use in section 4.

**4) Run ImageJ macro**

To run ‘Tubule analysis’ open ImageJ, select ‘Process’ -> ‘Batch’ -> ‘Macro’:


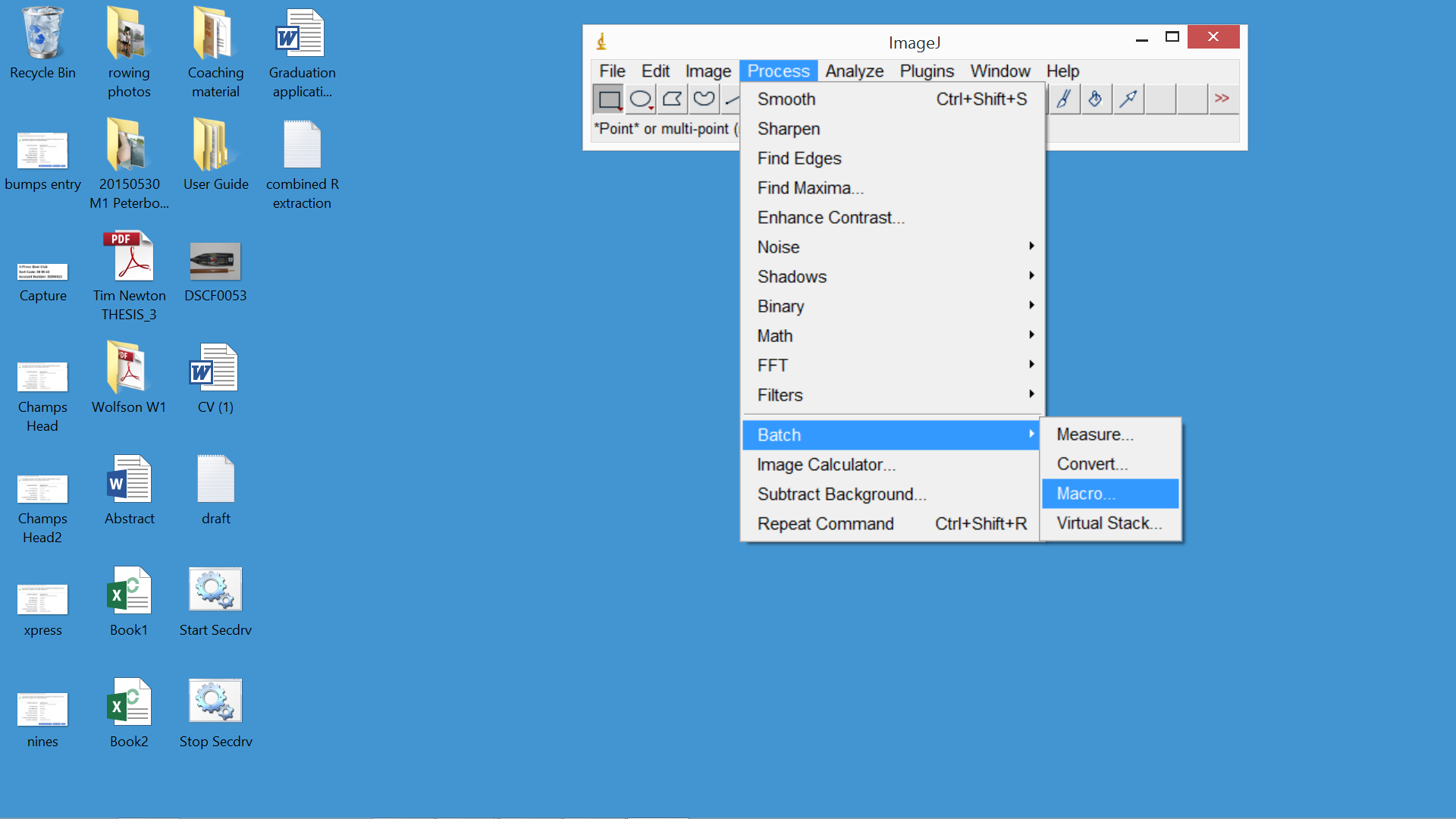


This opens the ‘Batch Process’ dialog box. Select the ‘input’ button and specify the folder containing ‘.tiff’ files produced during image collection:


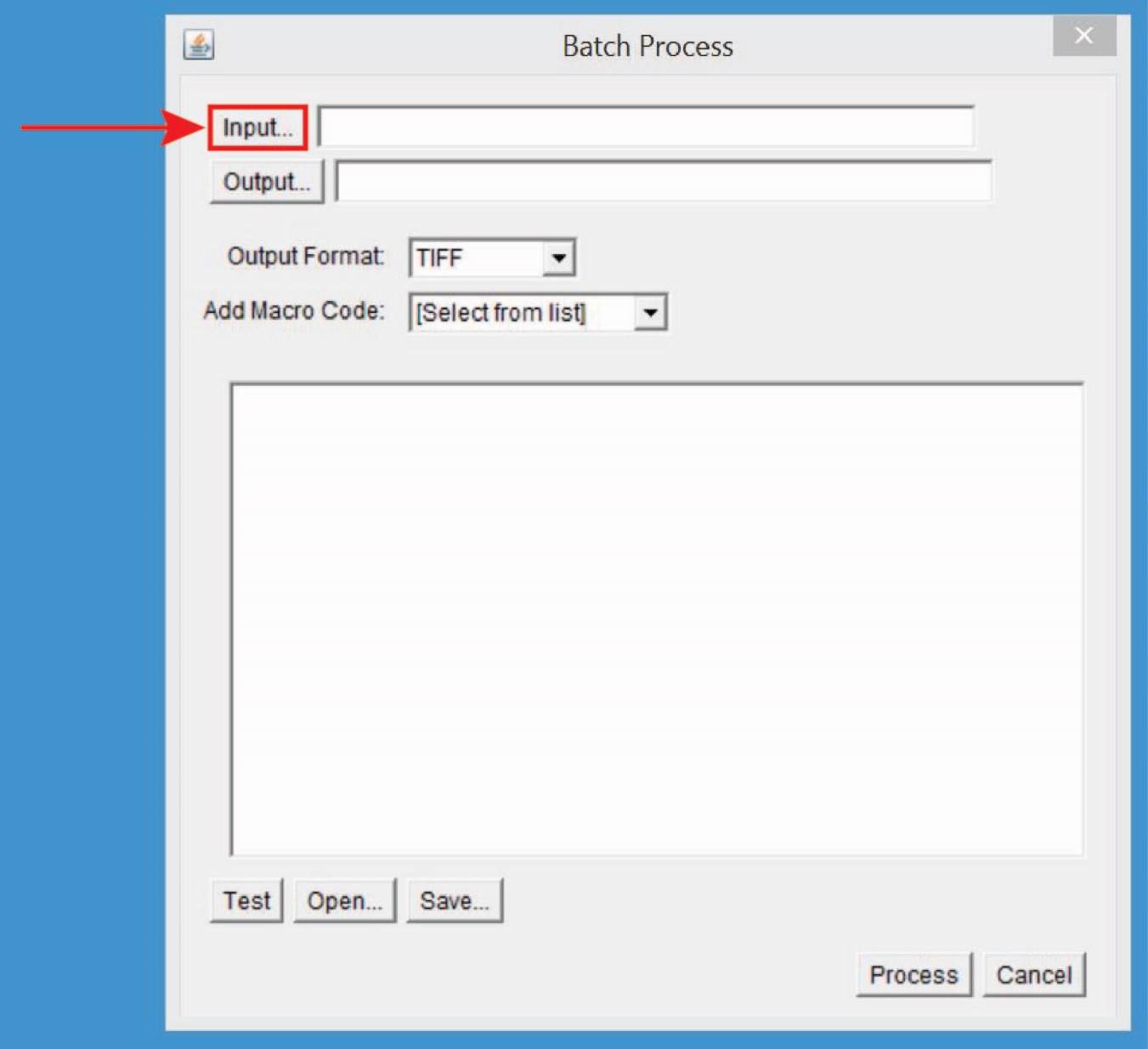


The macro is now selected by pressing the ‘Open’ button and selecting the ‘Tubule recognition’ ImageJ macro modified by the user in step 2:


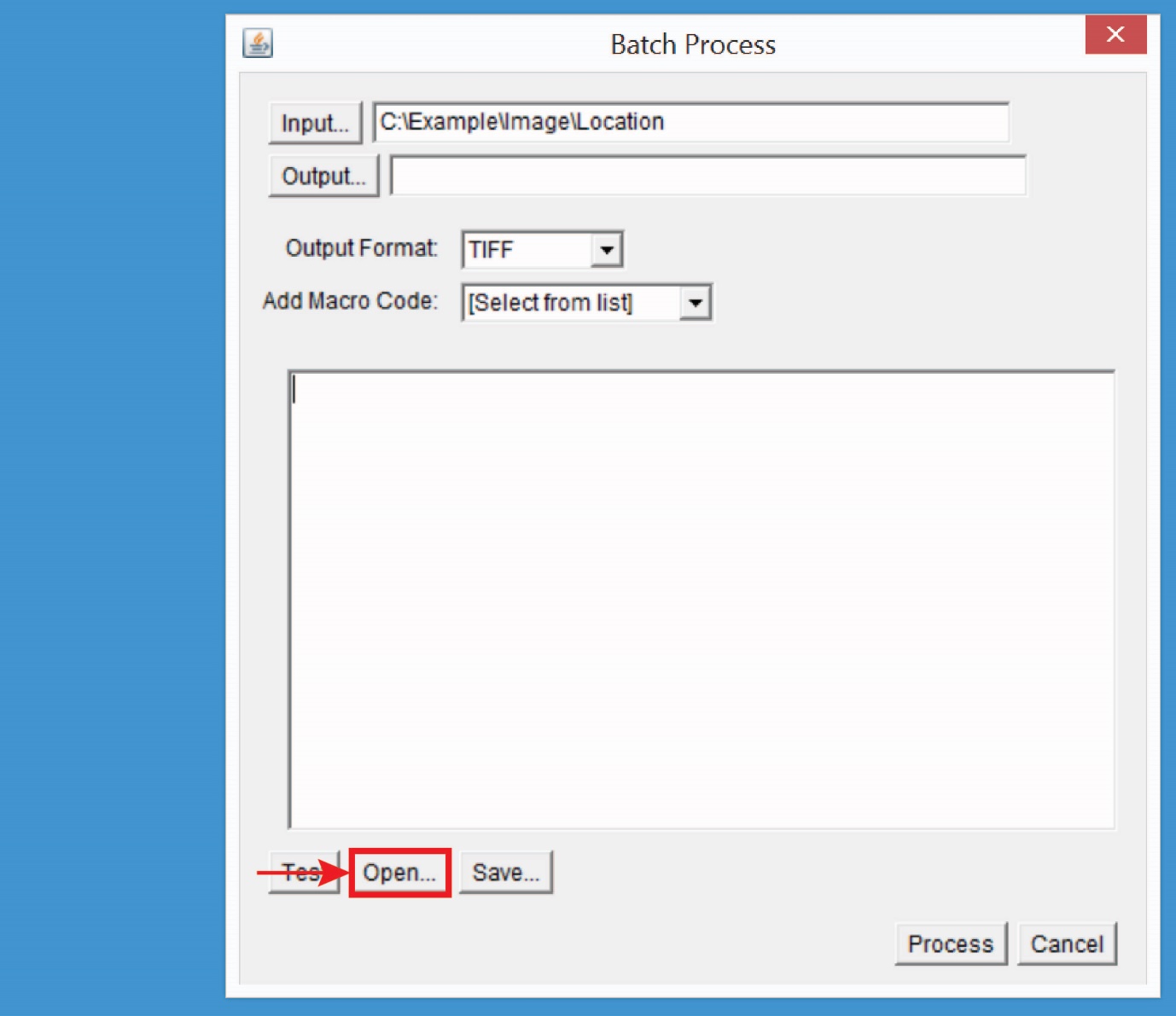


Finally, to start image analysis press the ‘Process’ button:


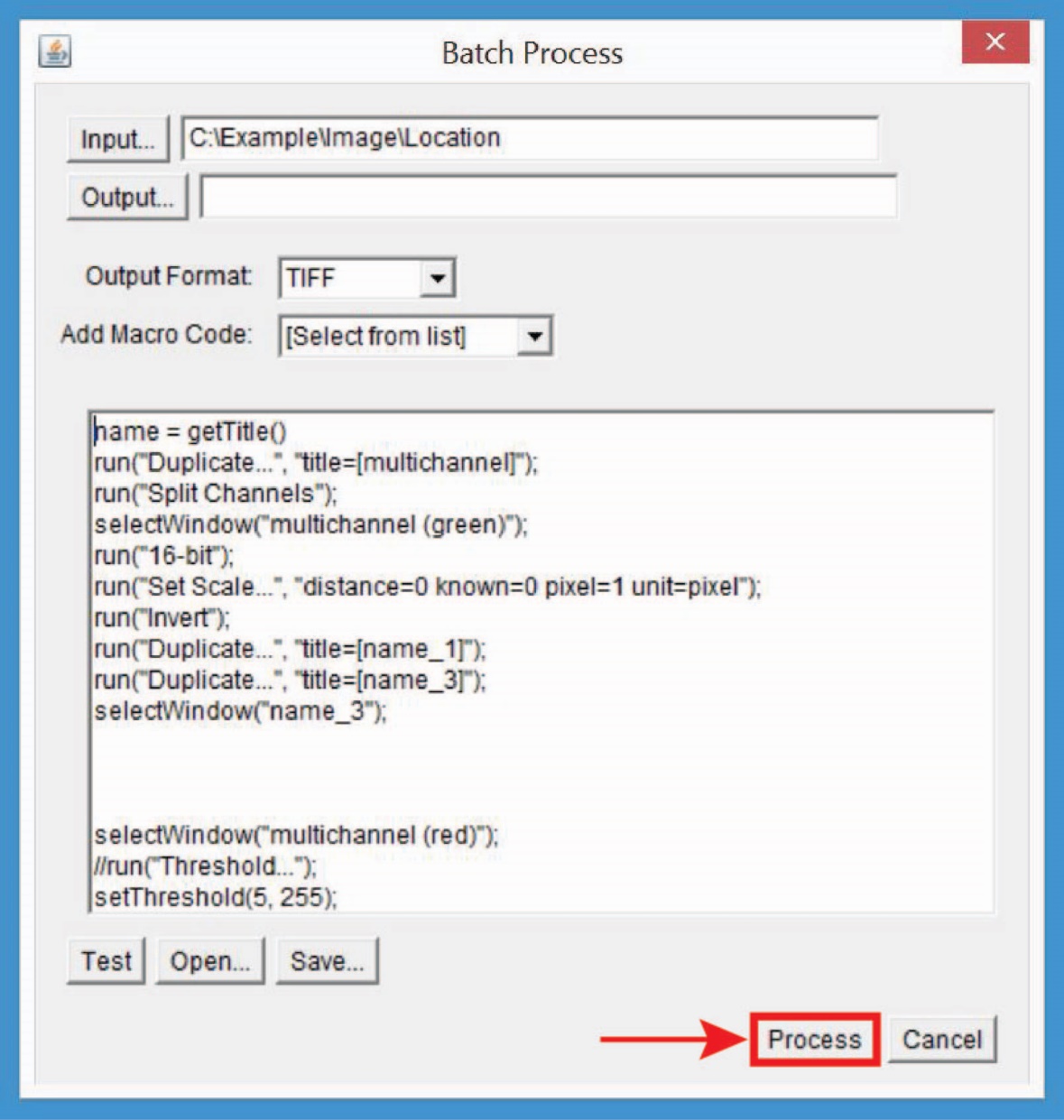


**5) R script modification**

Analysis of .csv files created by ImageJ can now be carried out using R. Before running the ‘Tubule Analysis’ R script modifications are required to specify where the .csv files can be found. Open ‘Tubule Analysis in R’ in a text editor:


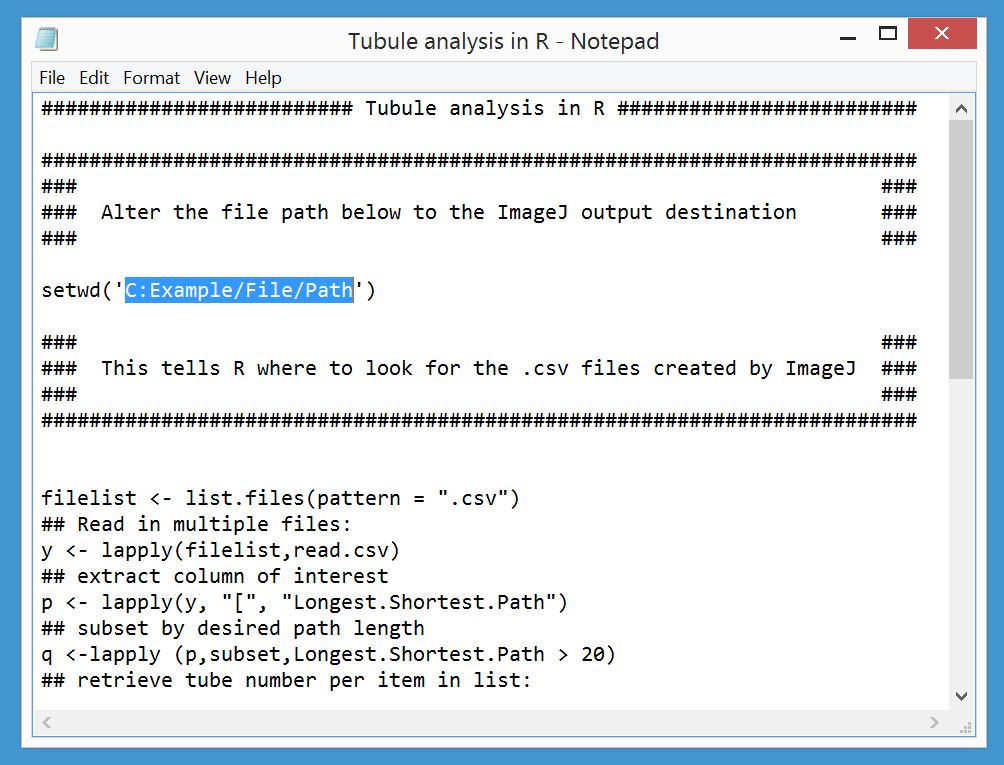


The text highlighted in blue must be modified to the filepath specified in the user in step 3. This sets the working directory of R to the same location as the stored .csv files created by ImageJ. This will also be where the results of the analysis are stored. Once modified save the R script ready for use in step 6.

**6) Analysis with R**

To analyse tubules select all the text in the R script modified in step 5, open R and paste the copied text into the R console:


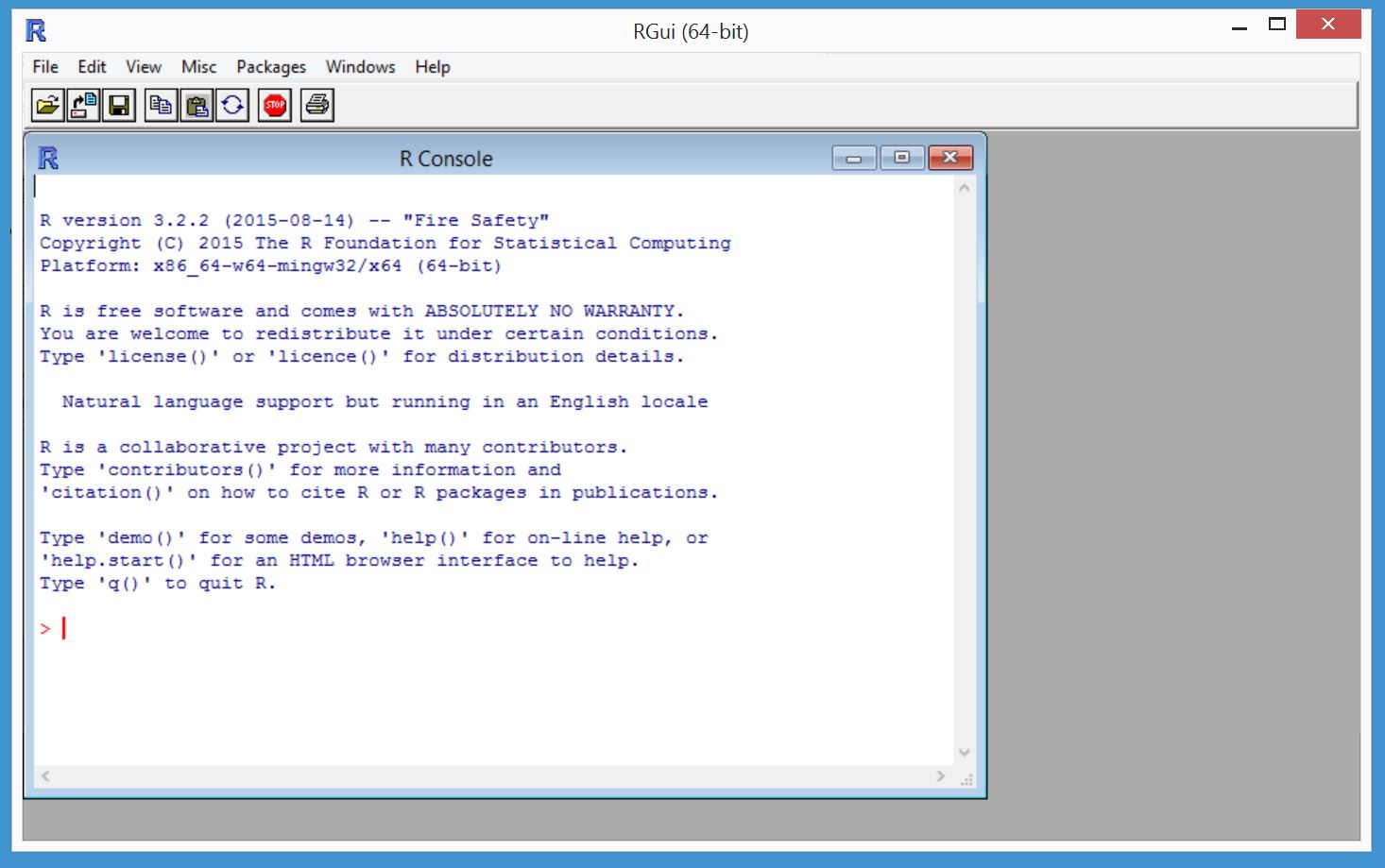


**7: Analysis in Excel**

The R script ‘Tubule Analysis in R’ will save a file named “output_results.txt” in the location specified by the user in steps 3 and 5. Insert this data into Microsoft Excel and group conditions by file name. For the tubule length and tubule number categories calculate the average value for each condition. For the tubule percentage category sum the values in each condition, divide this value by the number of images in the condition, then multiply by 100 to obtain the % of cells with tubules.

**8: Troubleshooting**

It is recommended the user runs the autocounting process using the example images and ensures that the results match those in the example results file. If this isn’t the case ImageJ settings need altering. These settings are located in your home directory -> .imageJ -> IJ_Prefs.txt.

Alter these settings as below:

bcolor=\#000000

fcolor=\#FFFFFF

Then add the line:

.bs.background=true
