# Supplementary figures and images for: An Automated Image Analysis System to Quantify Endosomal Tubulation
Source: PLoS One. 2016 Dec 22;11(12):e0168294. doi: 10.1371/journal.pone.0168294 (PMC5179261; doi:10.1371/journal.pone.0168294)

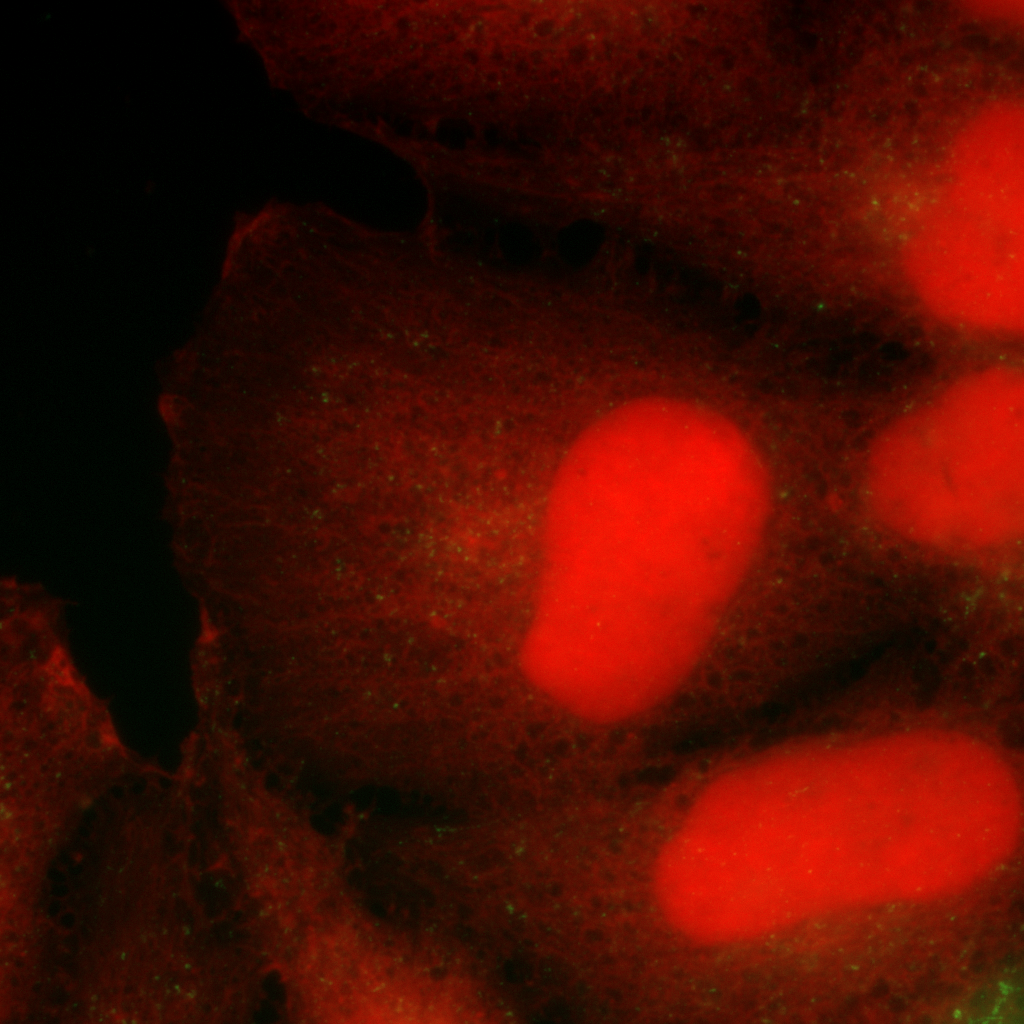

Supplement: S1 Dataset — Whole cell stain in red channel, endogenous SNX1 labelled in green channel. (ZIP) [file pone.0168294.s004.zip › Example Mock Images/Mock 1.tif]

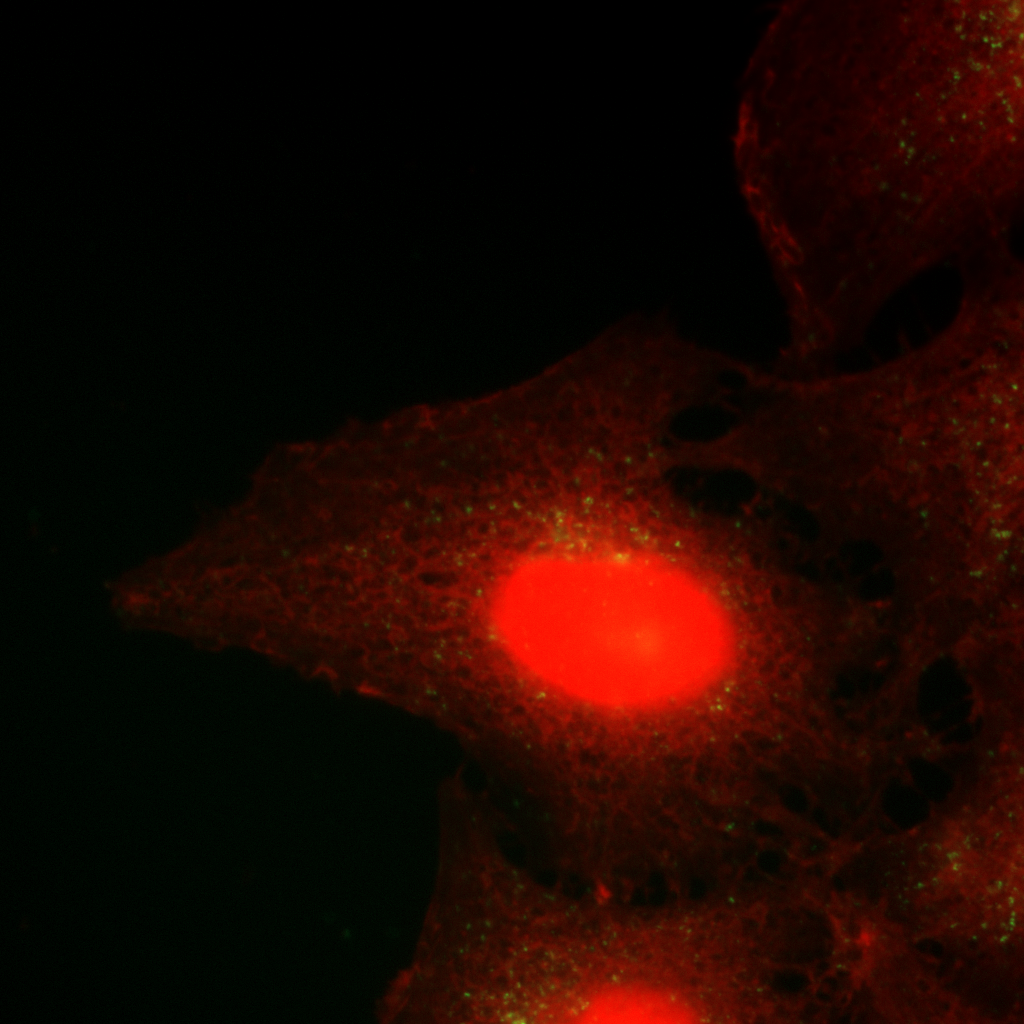

Supplement: S1 Dataset — Whole cell stain in red channel, endogenous SNX1 labelled in green channel. (ZIP) [file pone.0168294.s004.zip › Example Mock Images/Mock 10.tif]

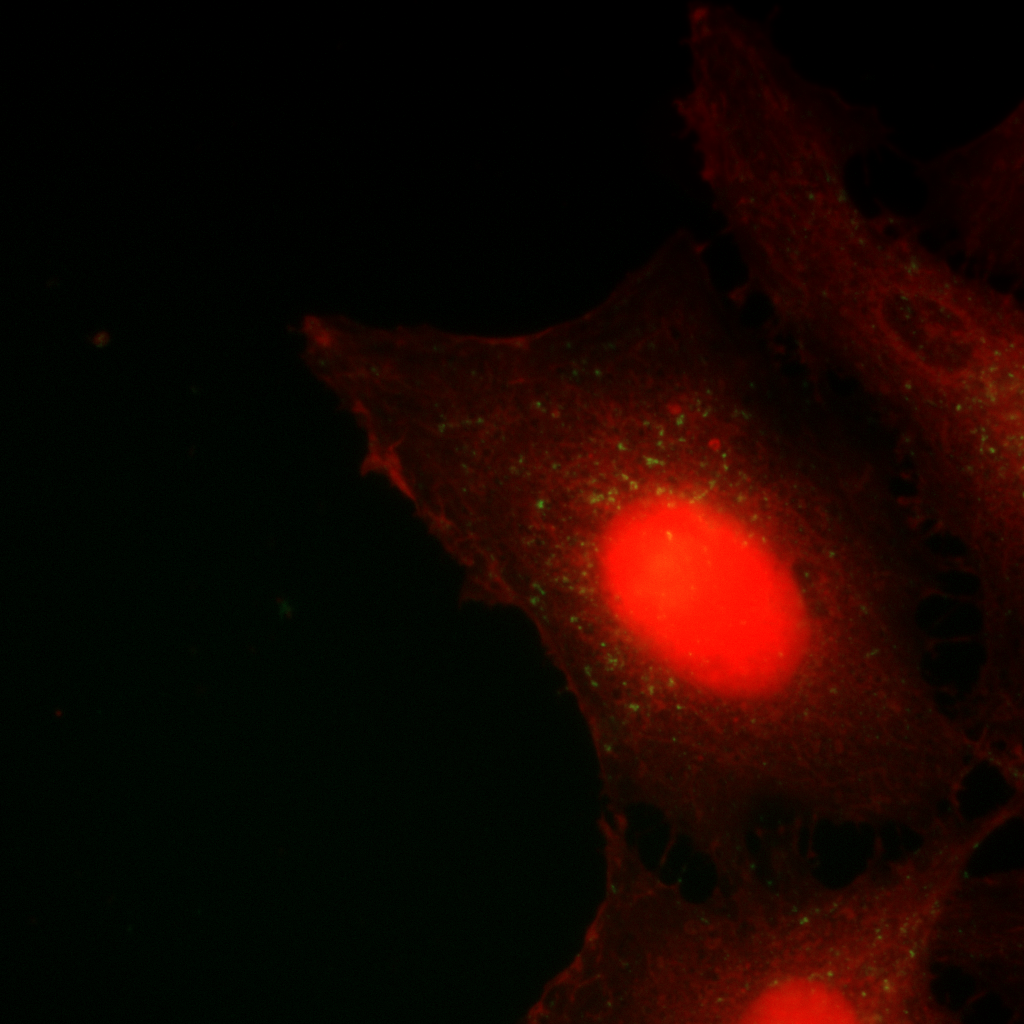

Supplement: S1 Dataset — Whole cell stain in red channel, endogenous SNX1 labelled in green channel. (ZIP) [file pone.0168294.s004.zip › Example Mock Images/Mock 11.tif]

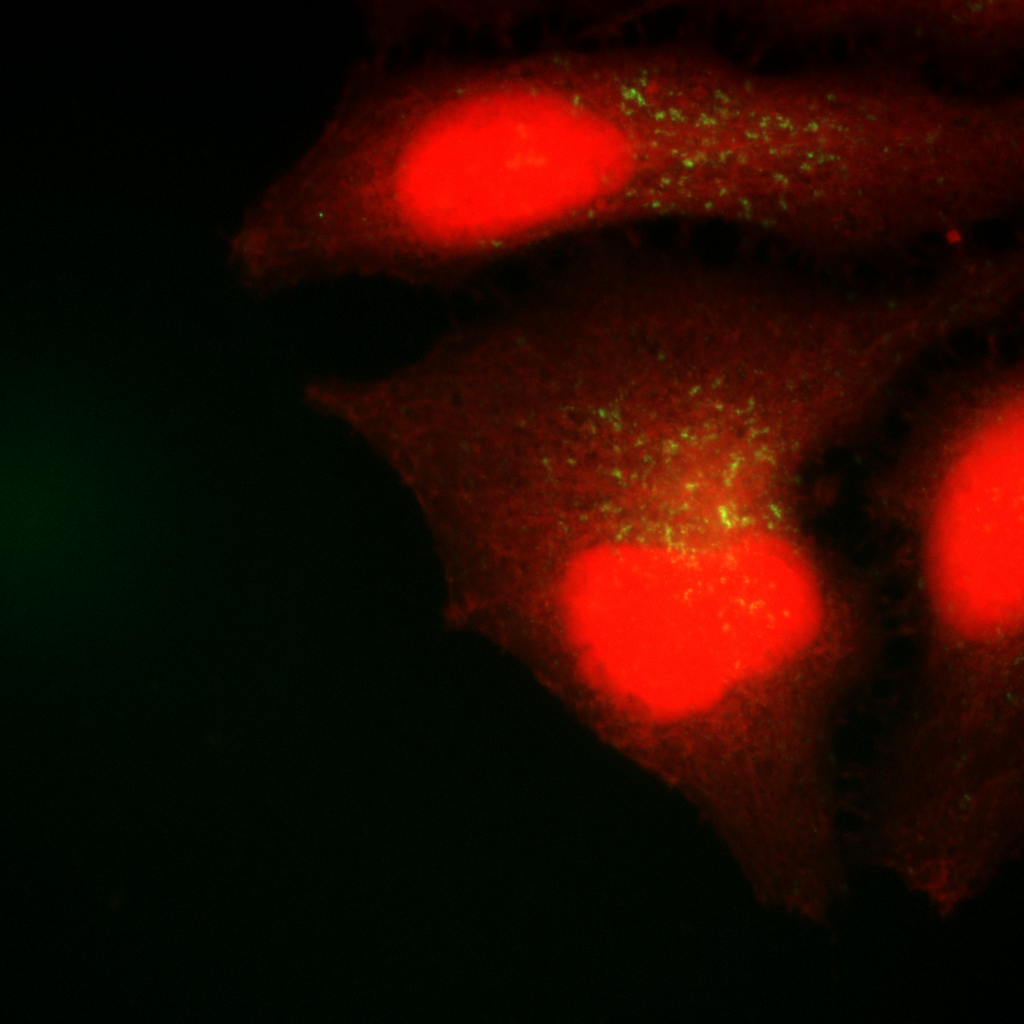

Supplement: S1 Dataset — Whole cell stain in red channel, endogenous SNX1 labelled in green channel. (ZIP) [file pone.0168294.s004.zip › Example Mock Images/Mock 12.tif]

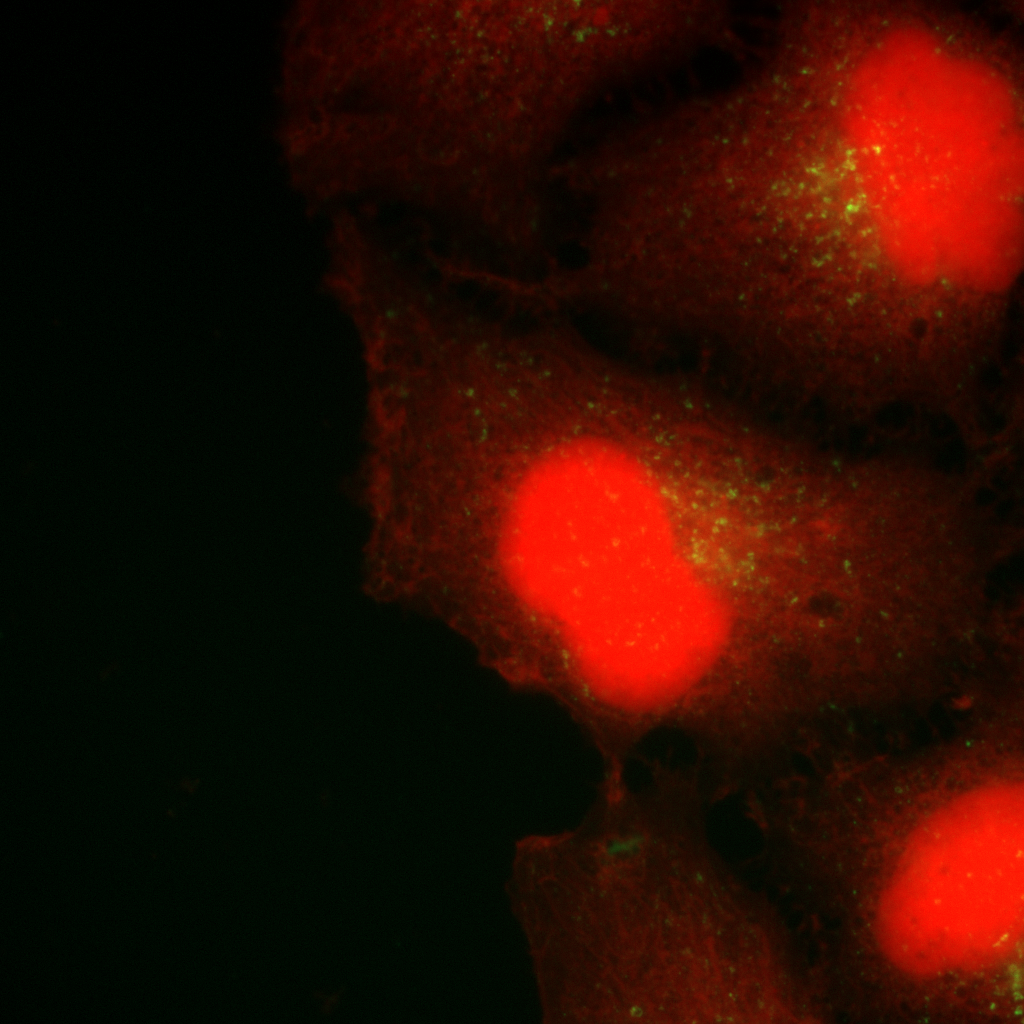

Supplement: S1 Dataset — Whole cell stain in red channel, endogenous SNX1 labelled in green channel. (ZIP) [file pone.0168294.s004.zip › Example Mock Images/Mock 13.tif]

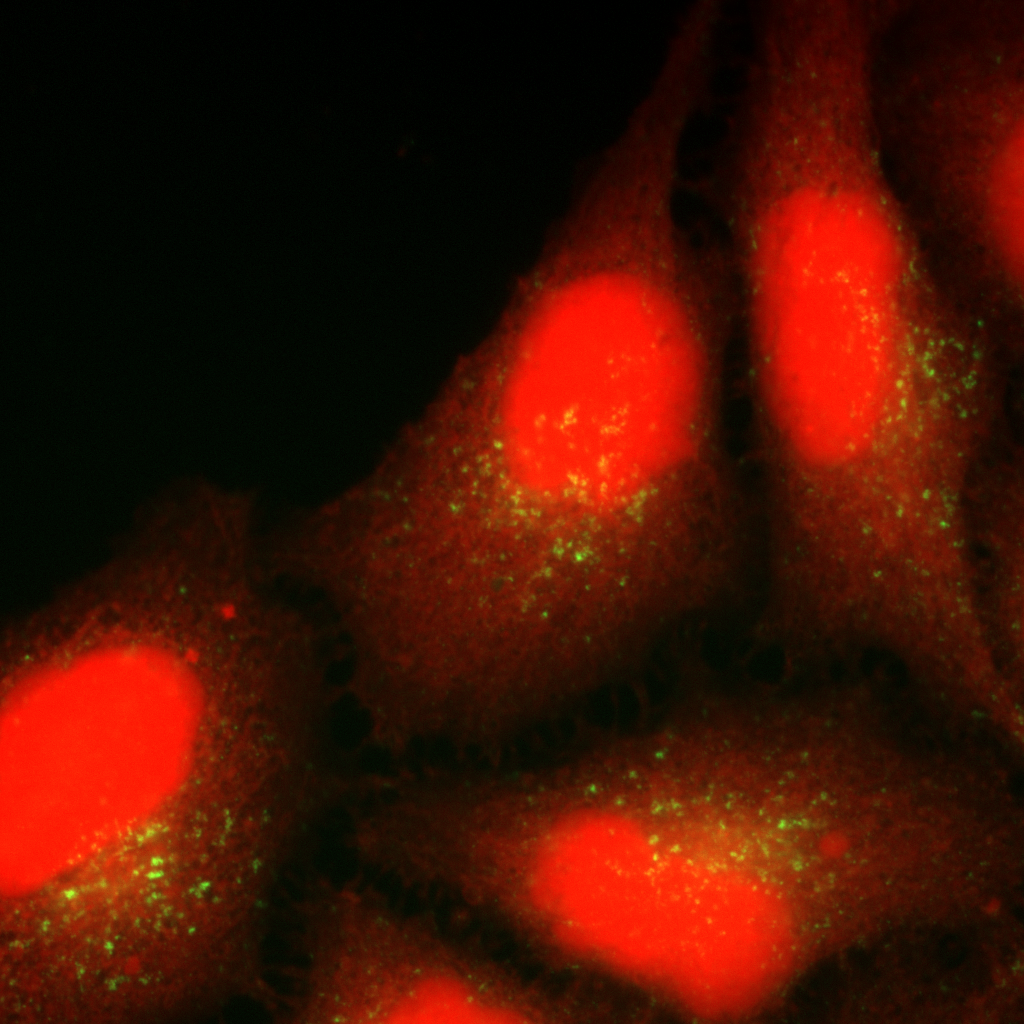

Supplement: S1 Dataset — Whole cell stain in red channel, endogenous SNX1 labelled in green channel. (ZIP) [file pone.0168294.s004.zip › Example Mock Images/Mock 14.tif]

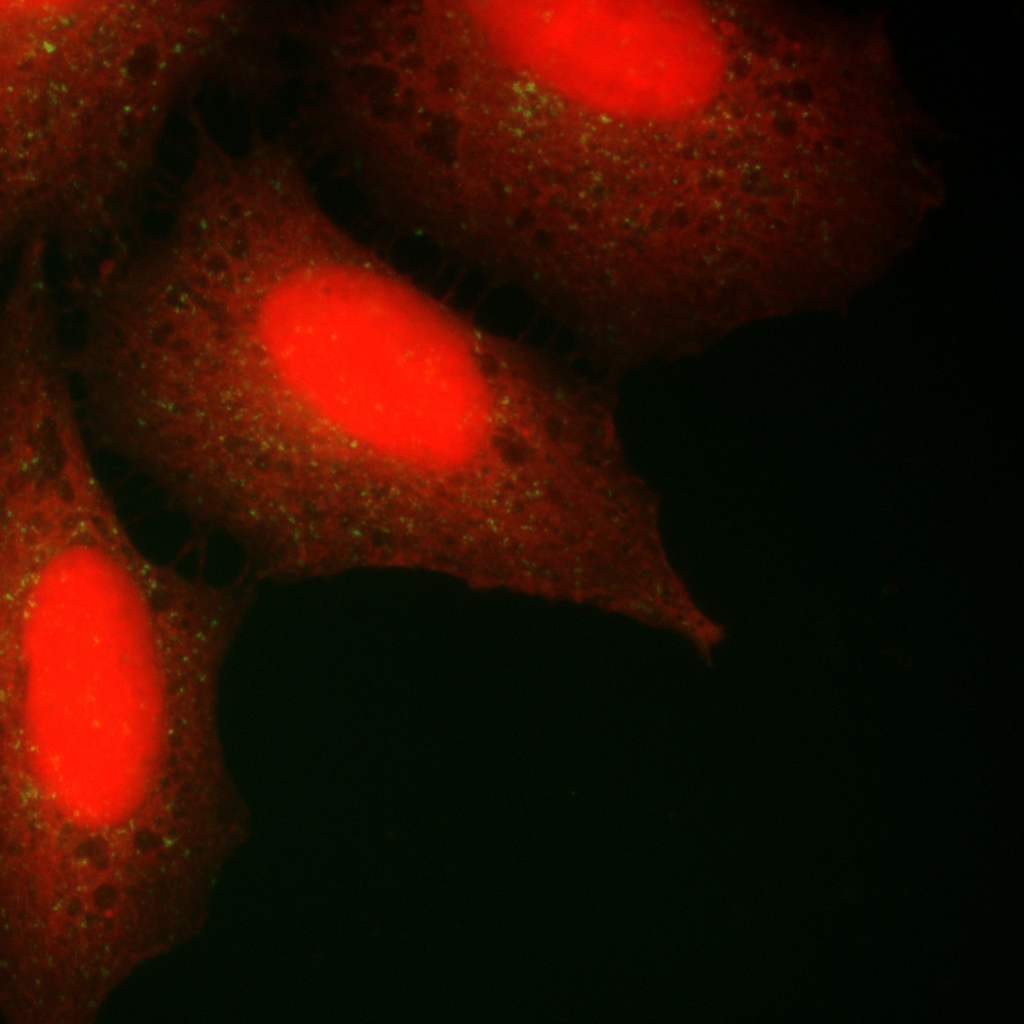

Supplement: S1 Dataset — Whole cell stain in red channel, endogenous SNX1 labelled in green channel. (ZIP) [file pone.0168294.s004.zip › Example Mock Images/Mock 15.tif]

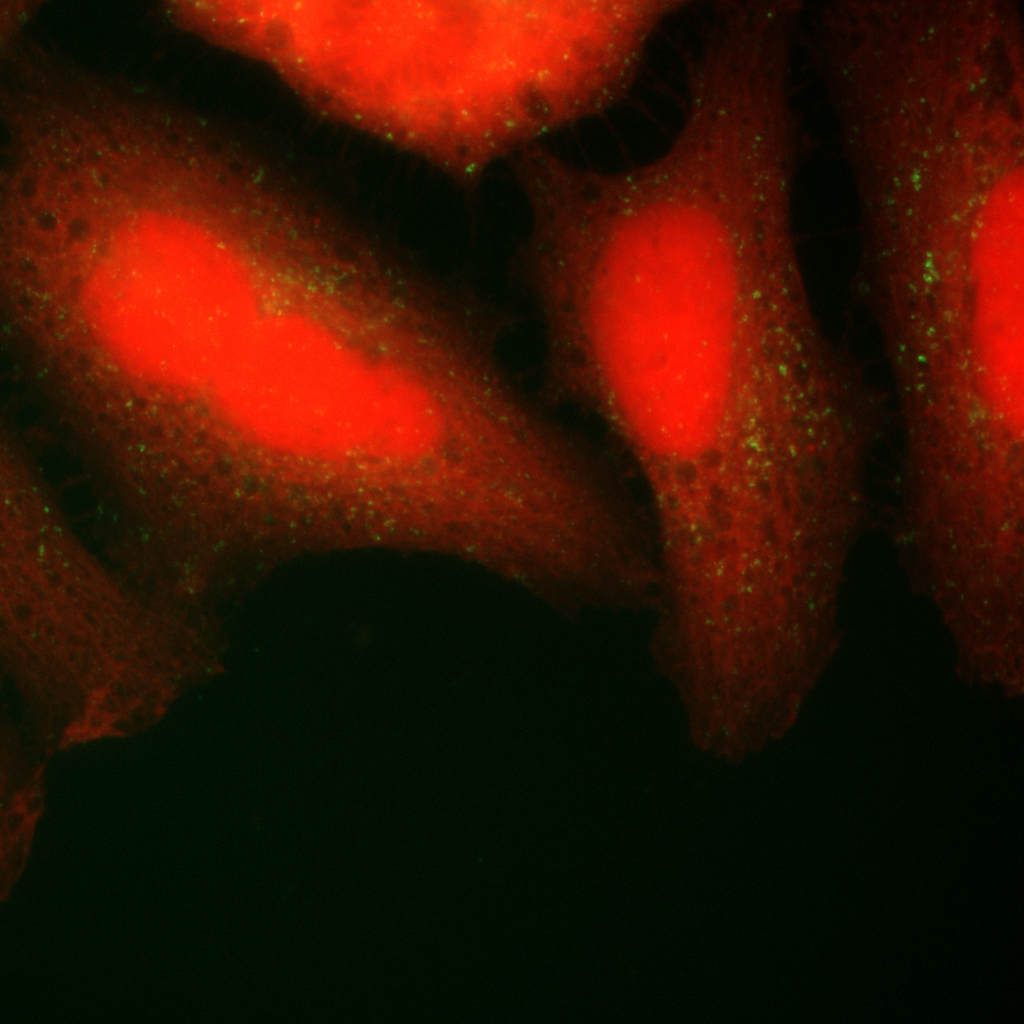

Supplement: S1 Dataset — Whole cell stain in red channel, endogenous SNX1 labelled in green channel. (ZIP) [file pone.0168294.s004.zip › Example Mock Images/Mock 16.tif]

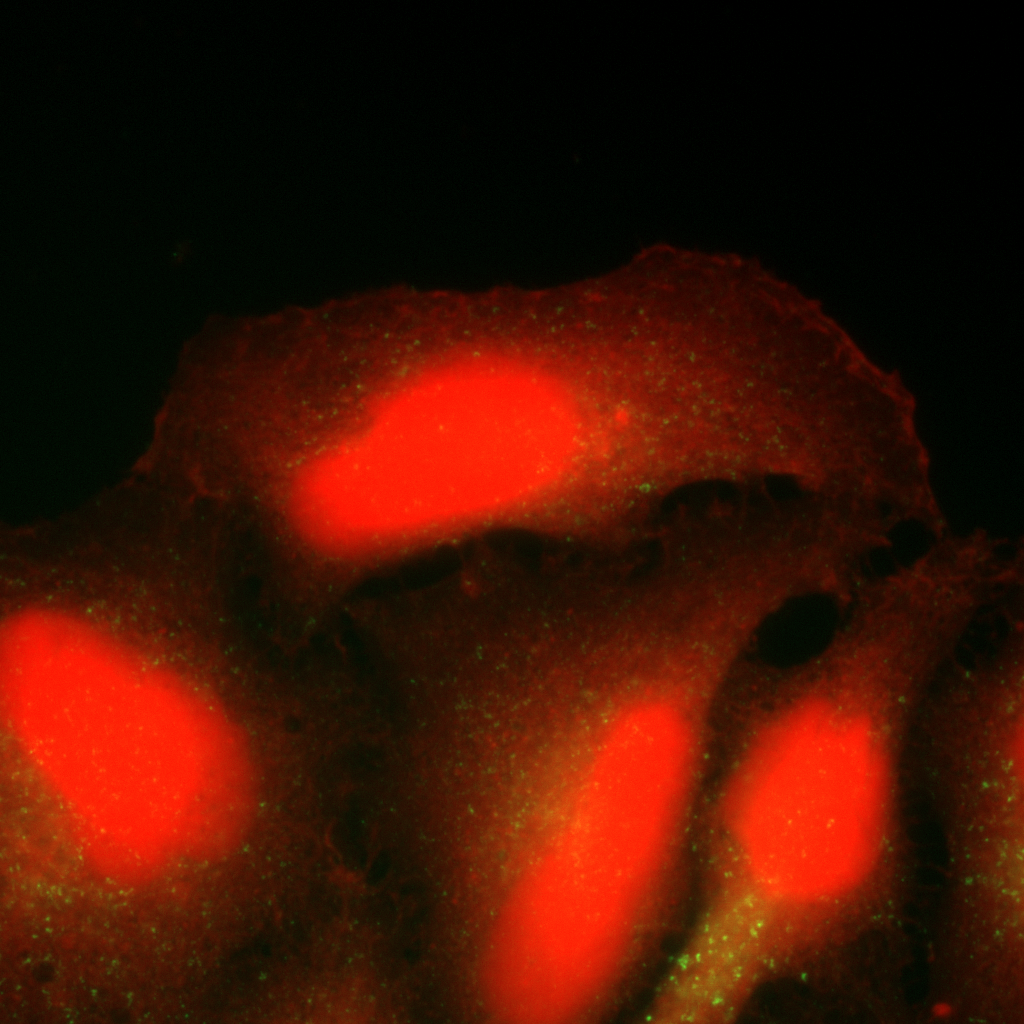

Supplement: S1 Dataset — Whole cell stain in red channel, endogenous SNX1 labelled in green channel. (ZIP) [file pone.0168294.s004.zip › Example Mock Images/Mock 17.tif]

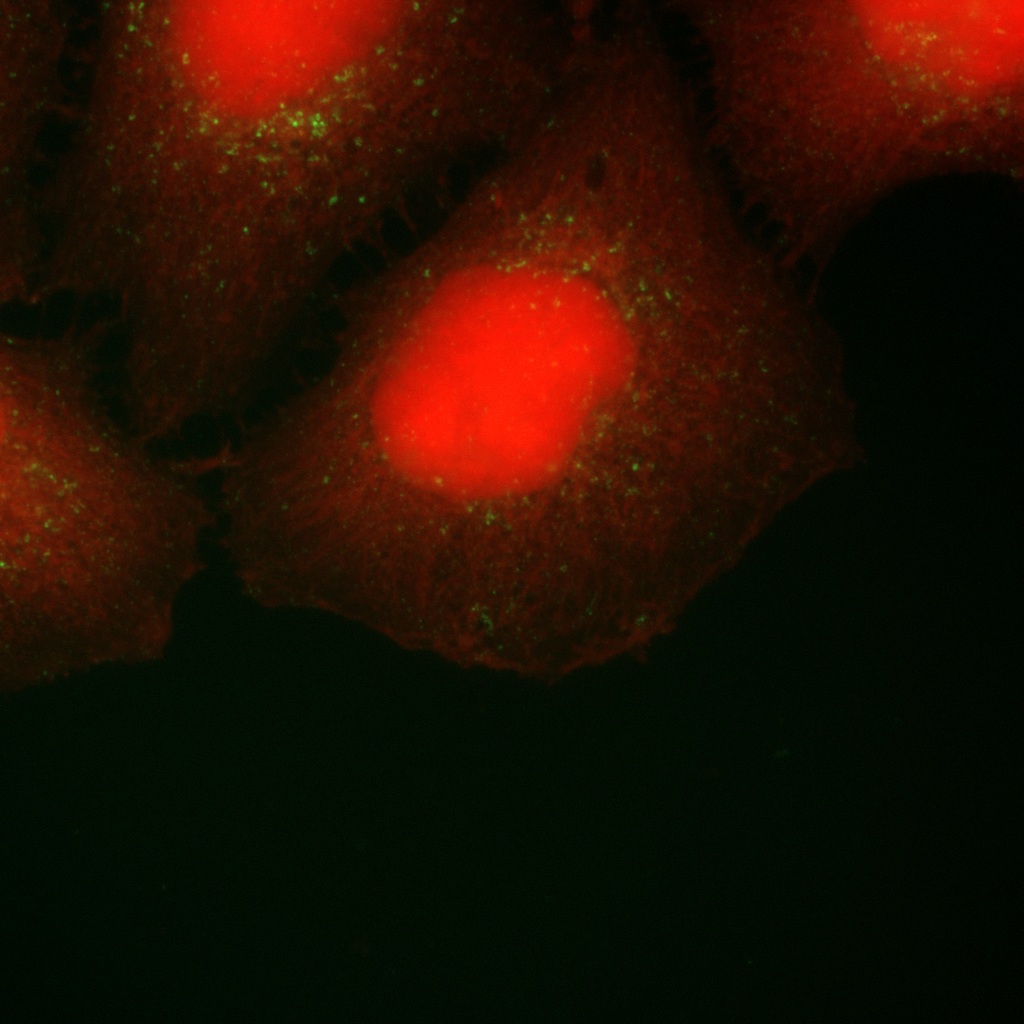

Supplement: S1 Dataset — Whole cell stain in red channel, endogenous SNX1 labelled in green channel. (ZIP) [file pone.0168294.s004.zip › Example Mock Images/Mock 18.tif]

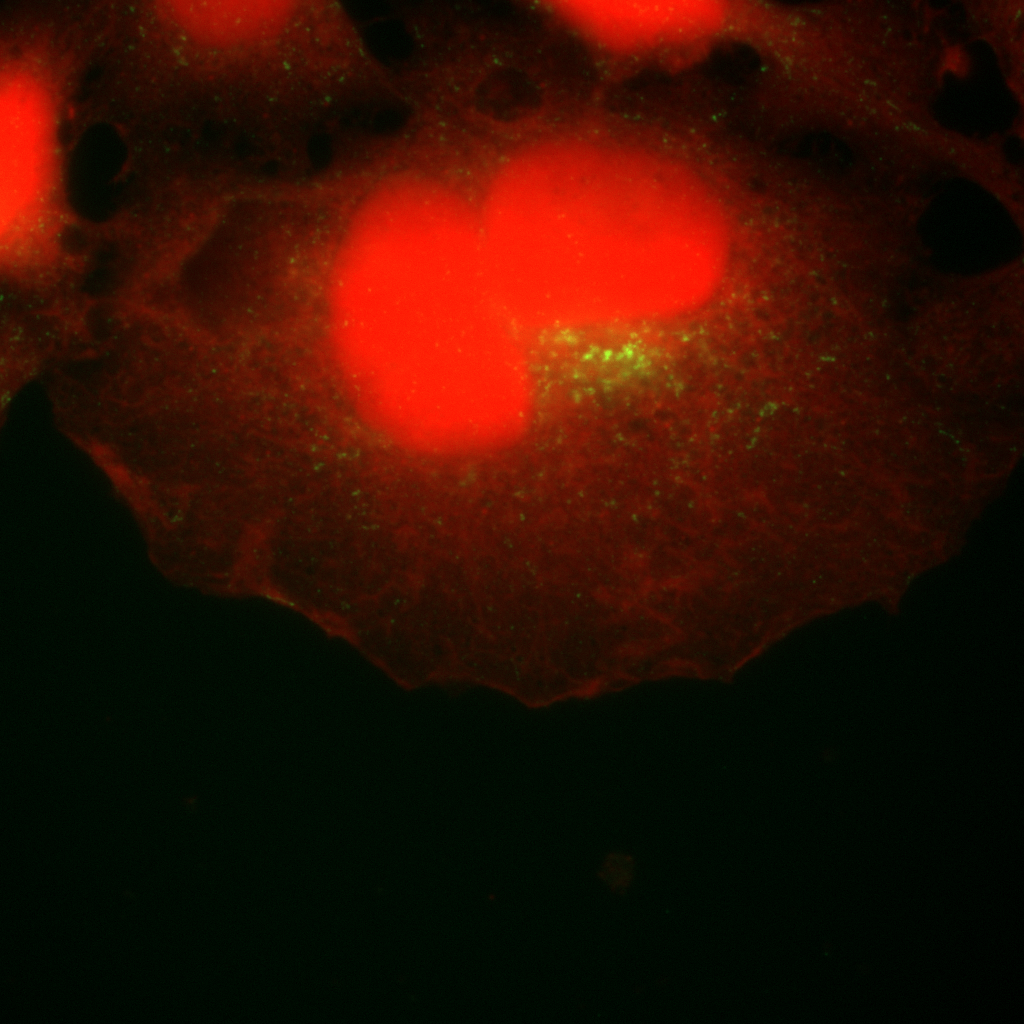

Supplement: S1 Dataset — Whole cell stain in red channel, endogenous SNX1 labelled in green channel. (ZIP) [file pone.0168294.s004.zip › Example Mock Images/Mock 19.tif]

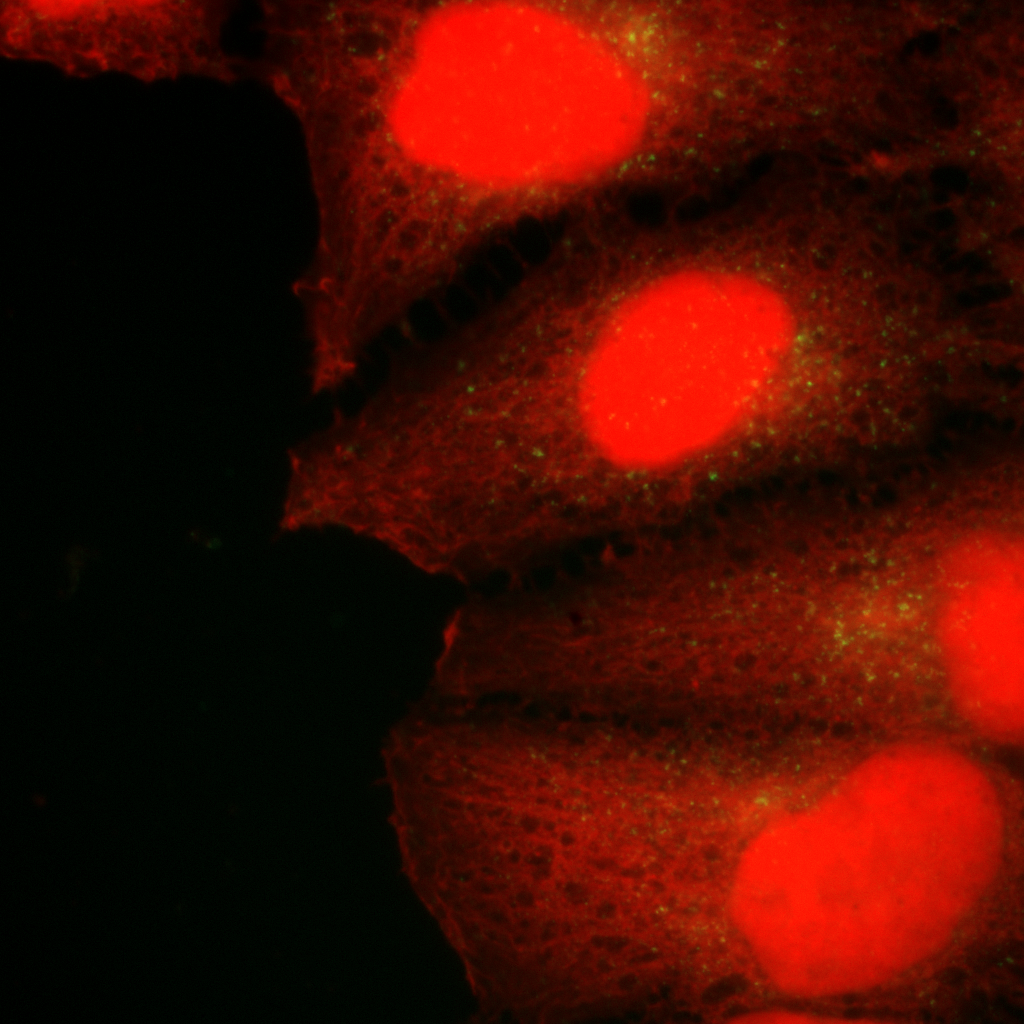

Supplement: S1 Dataset — Whole cell stain in red channel, endogenous SNX1 labelled in green channel. (ZIP) [file pone.0168294.s004.zip › Example Mock Images/Mock 2.tif]

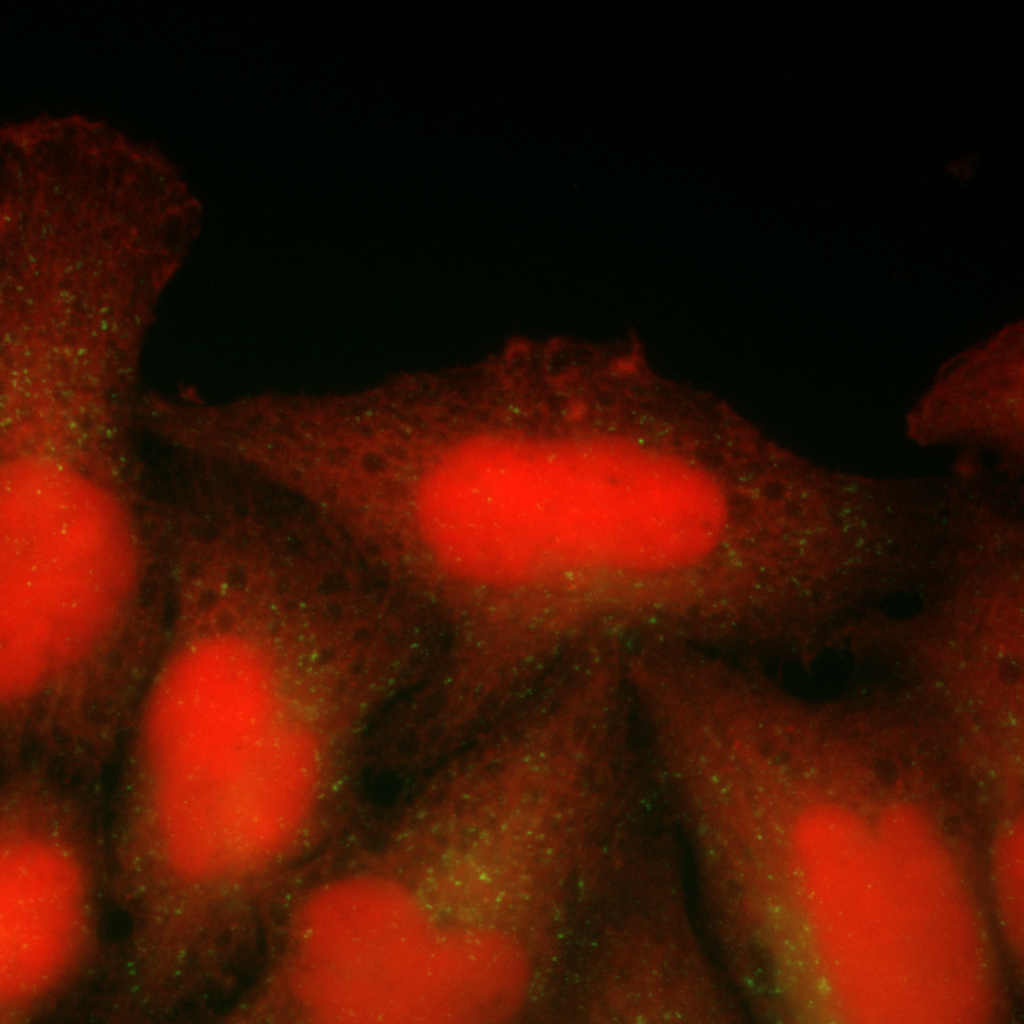

Supplement: S1 Dataset — Whole cell stain in red channel, endogenous SNX1 labelled in green channel. (ZIP) [file pone.0168294.s004.zip › Example Mock Images/Mock 20.tif]

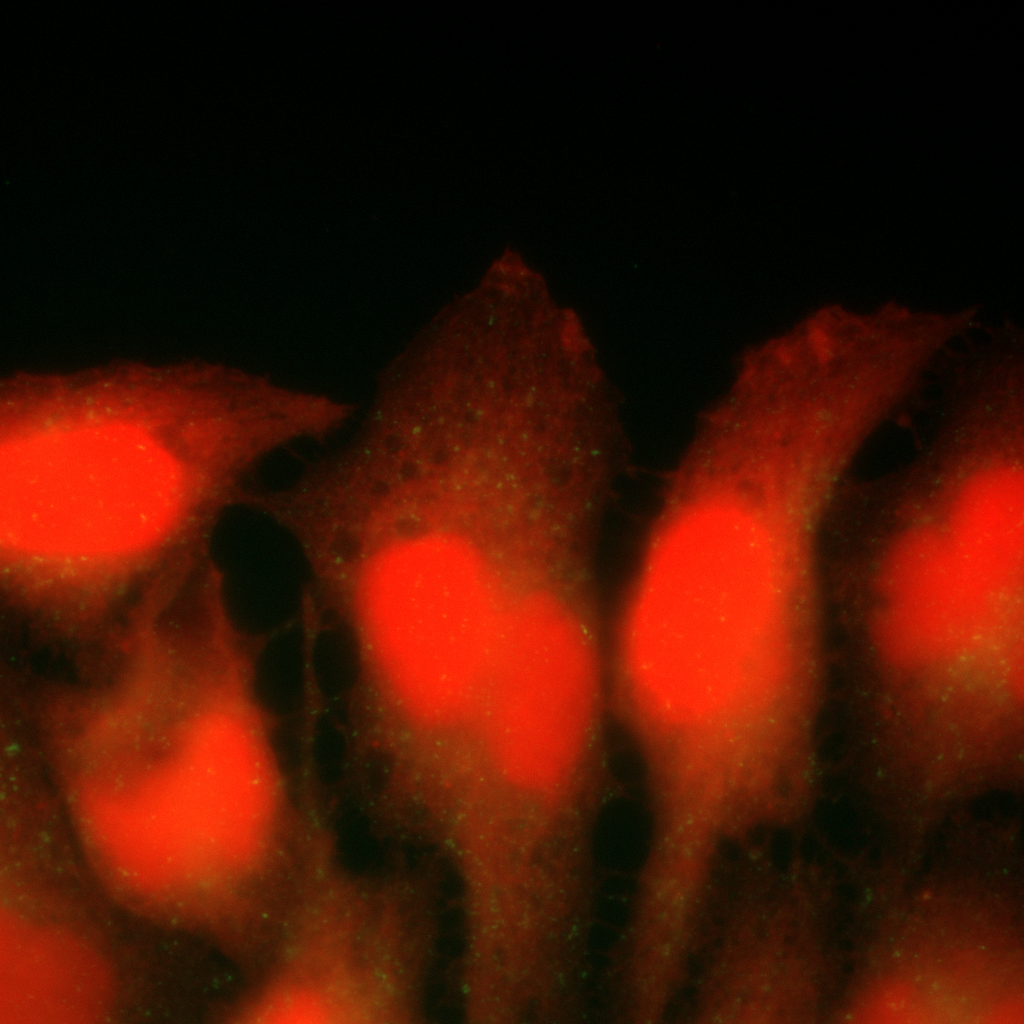

Supplement: S1 Dataset — Whole cell stain in red channel, endogenous SNX1 labelled in green channel. (ZIP) [file pone.0168294.s004.zip › Example Mock Images/Mock 21.tif]

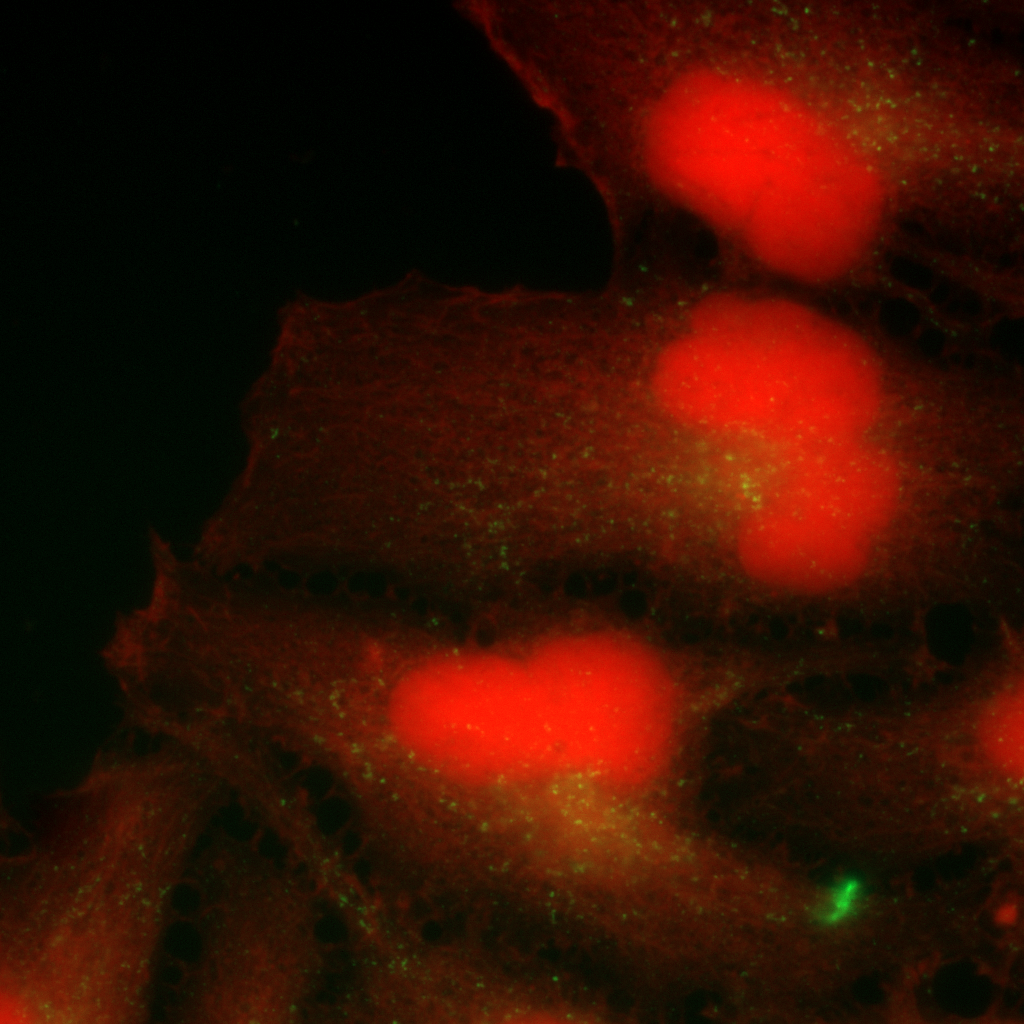

Supplement: S1 Dataset — Whole cell stain in red channel, endogenous SNX1 labelled in green channel. (ZIP) [file pone.0168294.s004.zip › Example Mock Images/Mock 22.tif]

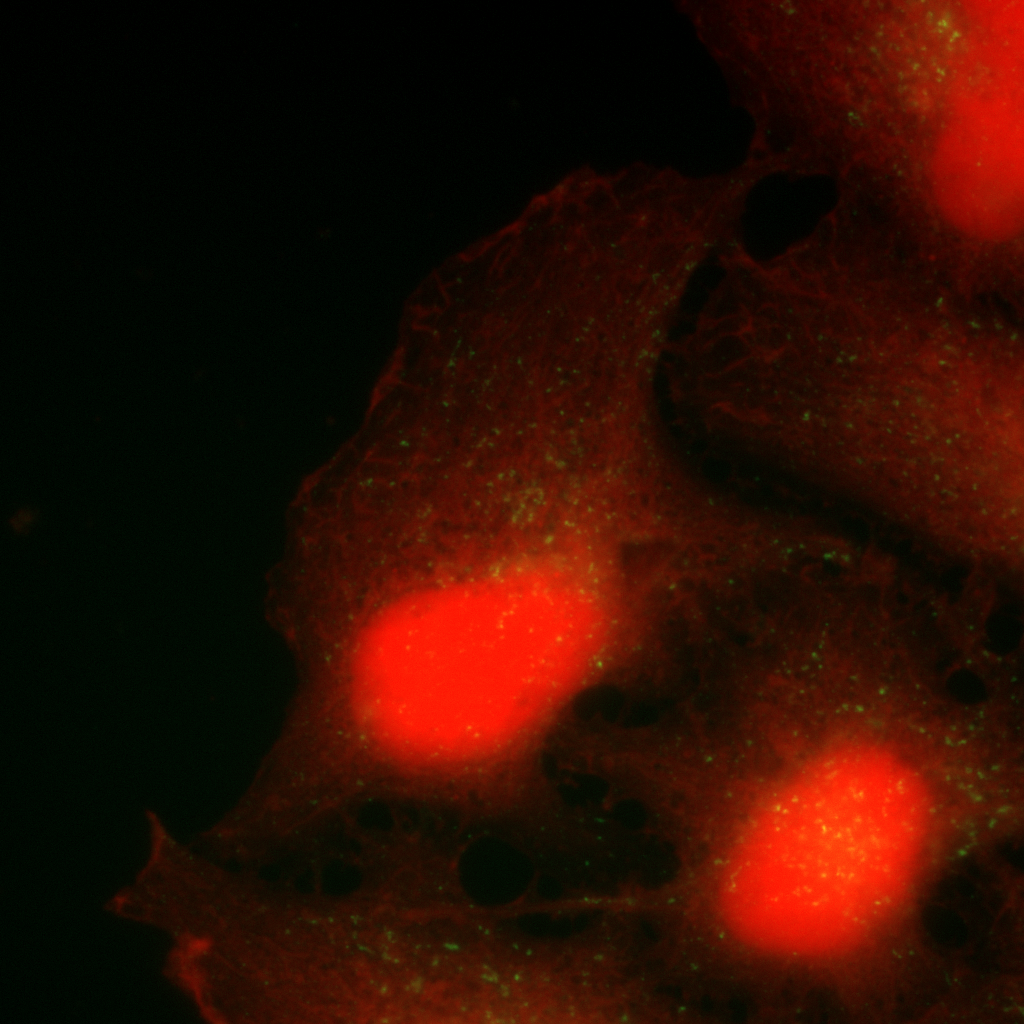

Supplement: S1 Dataset — Whole cell stain in red channel, endogenous SNX1 labelled in green channel. (ZIP) [file pone.0168294.s004.zip › Example Mock Images/Mock 23.tif]

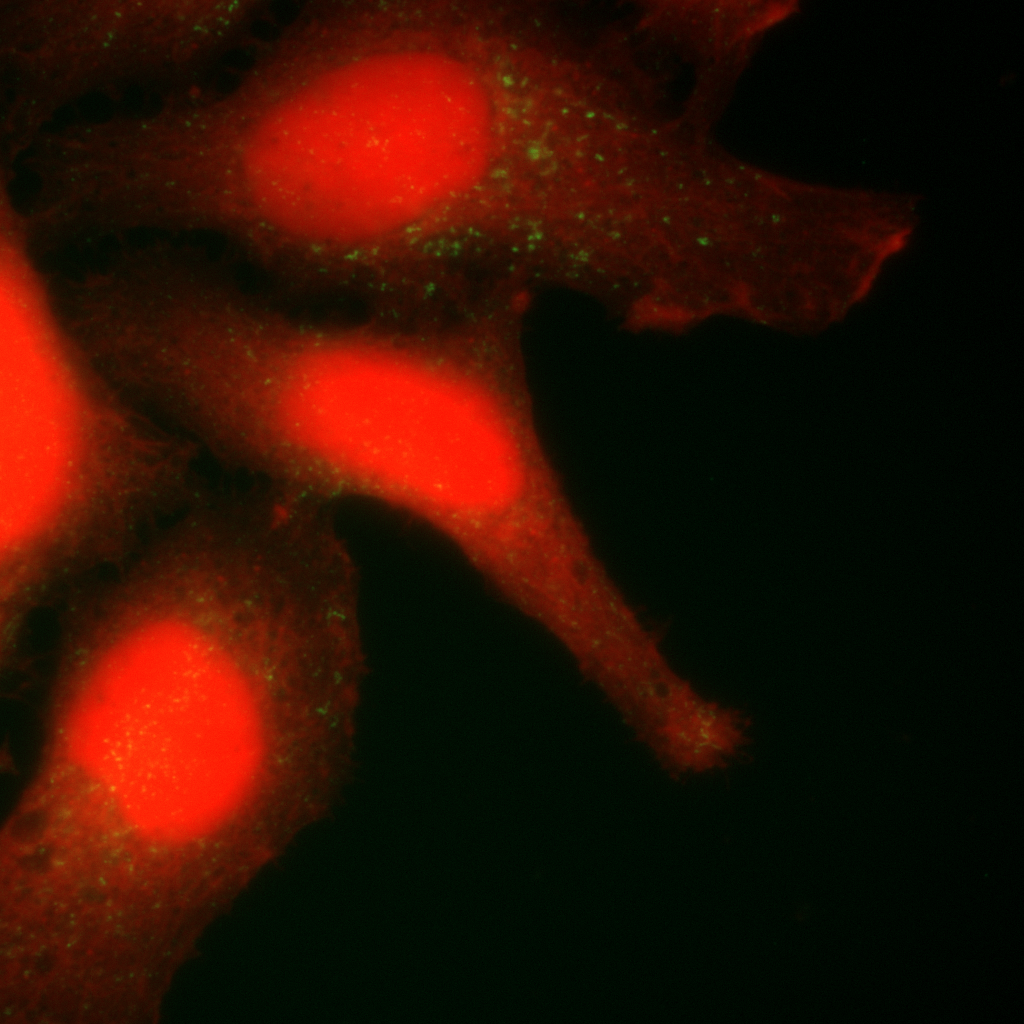

Supplement: S1 Dataset — Whole cell stain in red channel, endogenous SNX1 labelled in green channel. (ZIP) [file pone.0168294.s004.zip › Example Mock Images/Mock 24.tif]

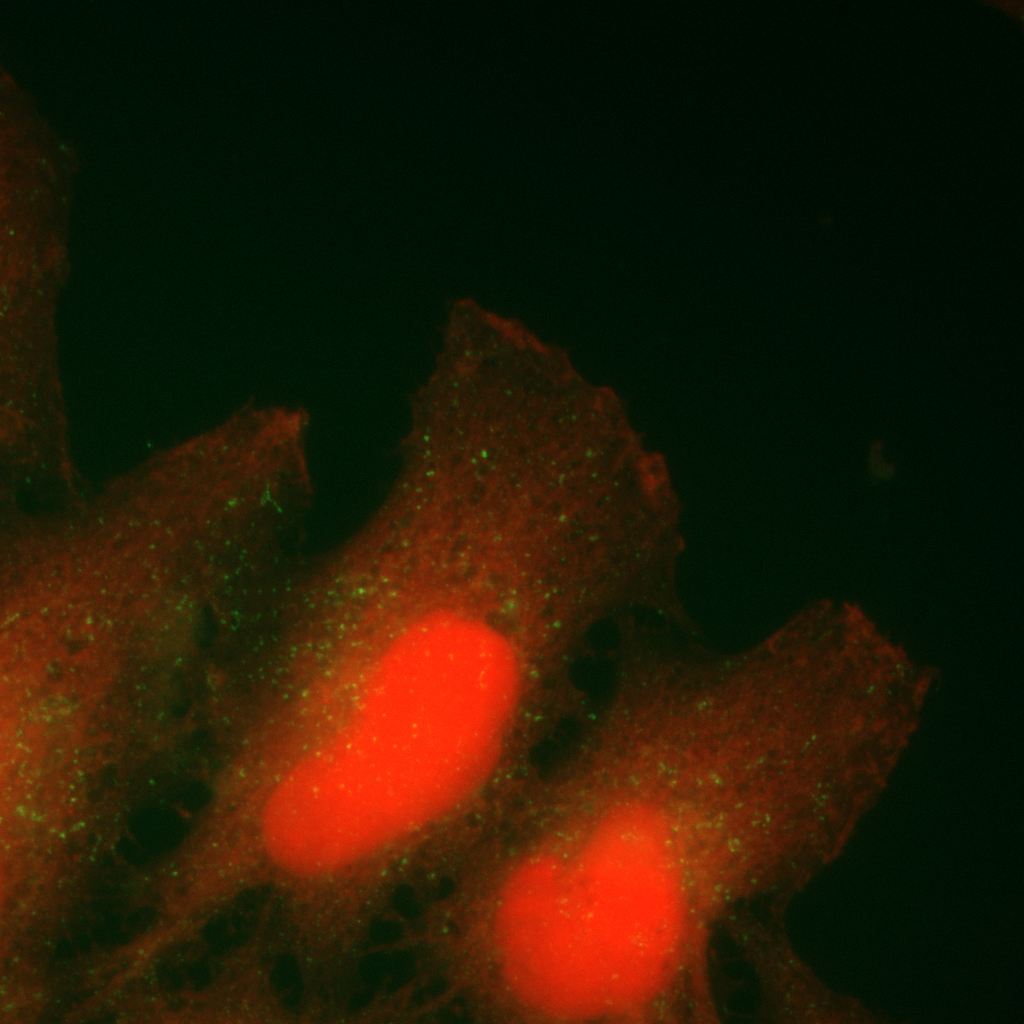

Supplement: S1 Dataset — Whole cell stain in red channel, endogenous SNX1 labelled in green channel. (ZIP) [file pone.0168294.s004.zip › Example Mock Images/Mock 25.tif]

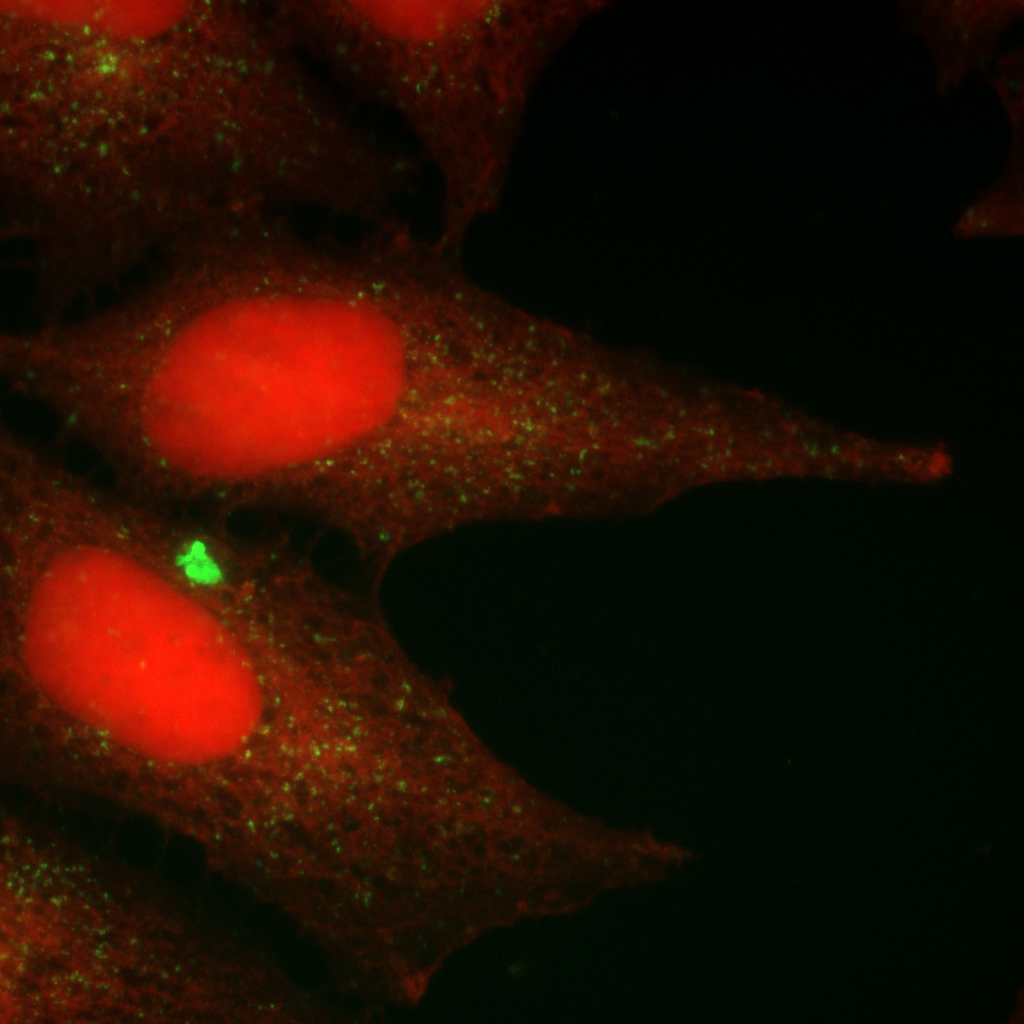

Supplement: S1 Dataset — Whole cell stain in red channel, endogenous SNX1 labelled in green channel. (ZIP) [file pone.0168294.s004.zip › Example Mock Images/Mock 26.tif]

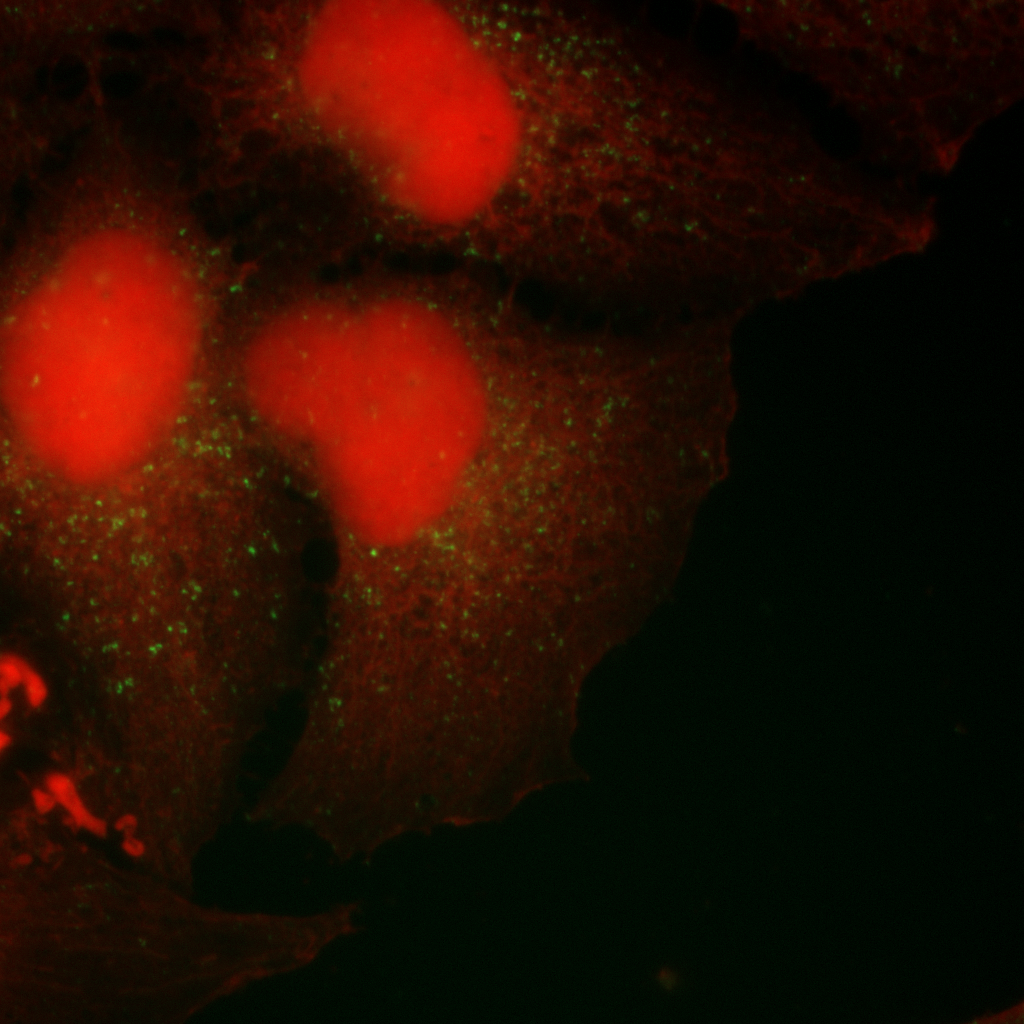

Supplement: S1 Dataset — Whole cell stain in red channel, endogenous SNX1 labelled in green channel. (ZIP) [file pone.0168294.s004.zip › Example Mock Images/Mock 27.tif]

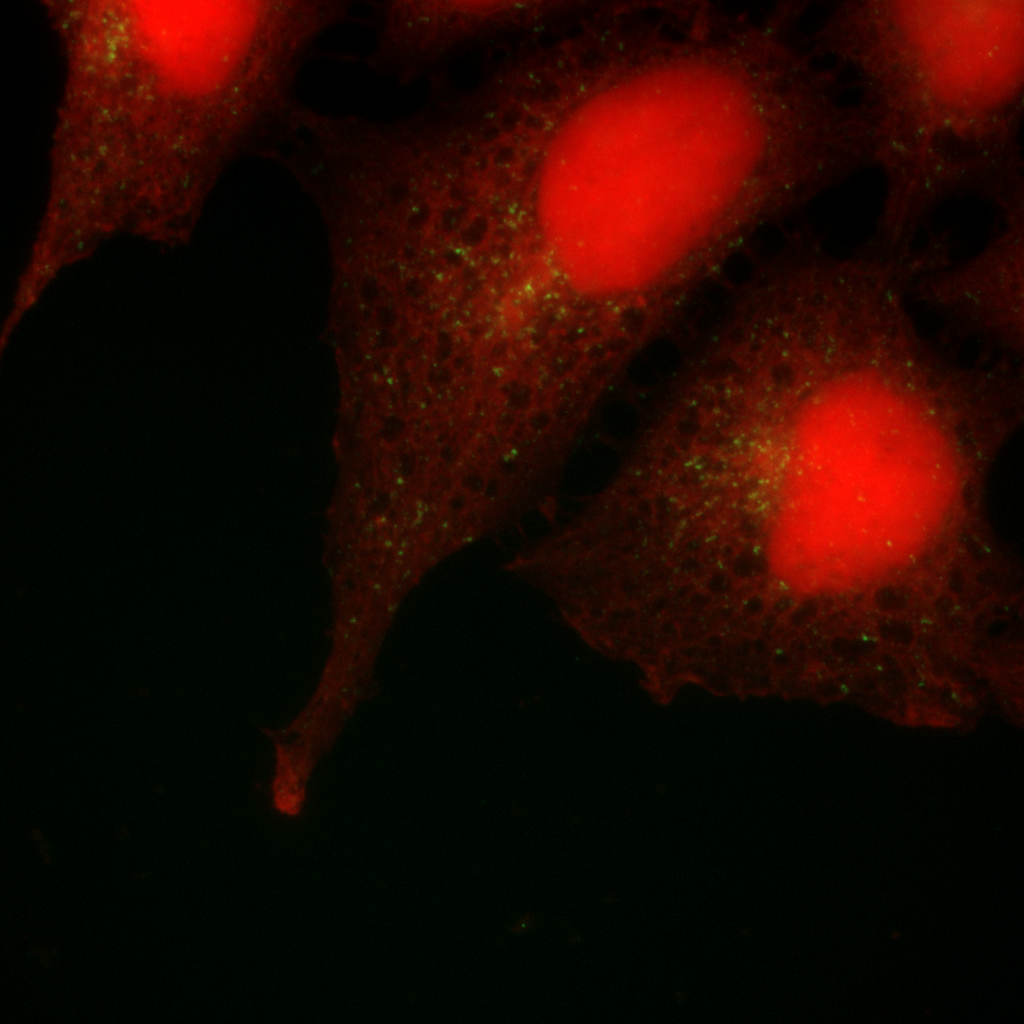

Supplement: S1 Dataset — Whole cell stain in red channel, endogenous SNX1 labelled in green channel. (ZIP) [file pone.0168294.s004.zip › Example Mock Images/Mock 28.tif]

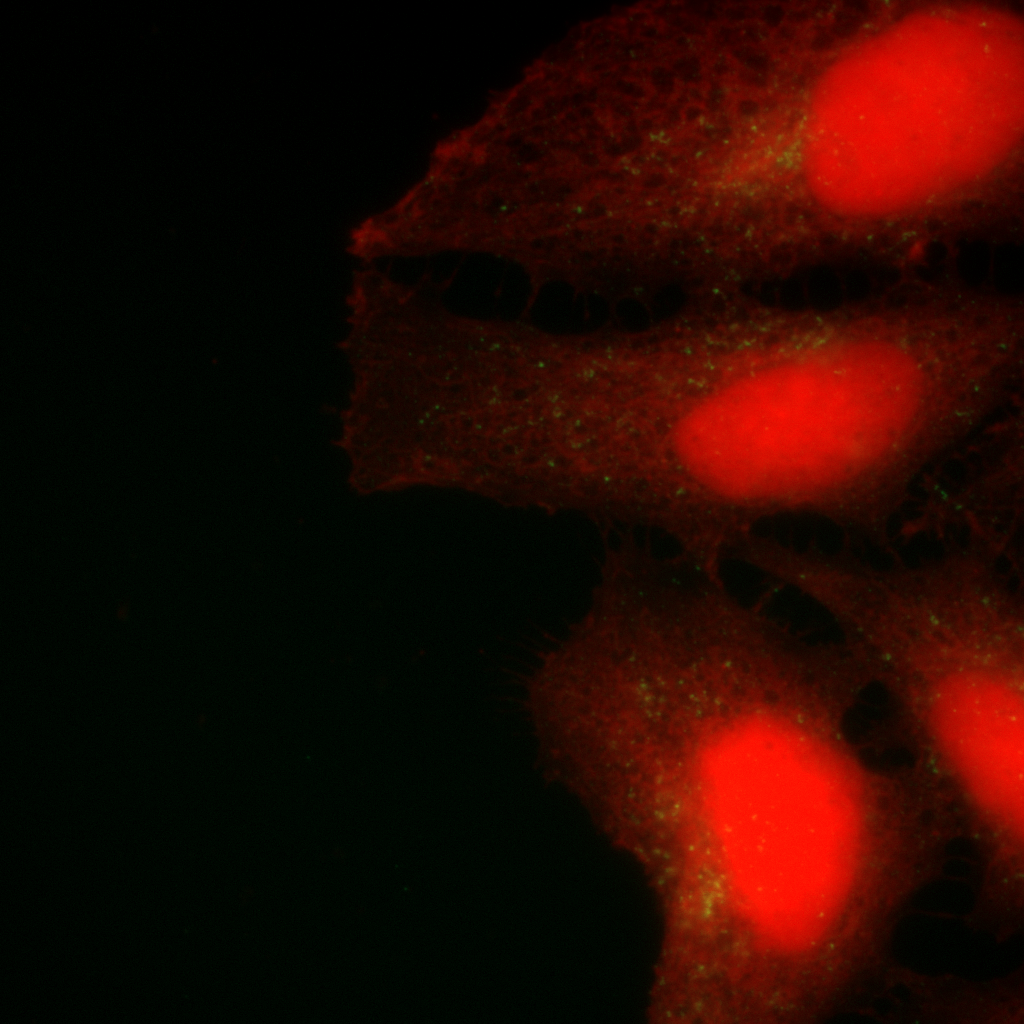

Supplement: S1 Dataset — Whole cell stain in red channel, endogenous SNX1 labelled in green channel. (ZIP) [file pone.0168294.s004.zip › Example Mock Images/Mock 29.tif]

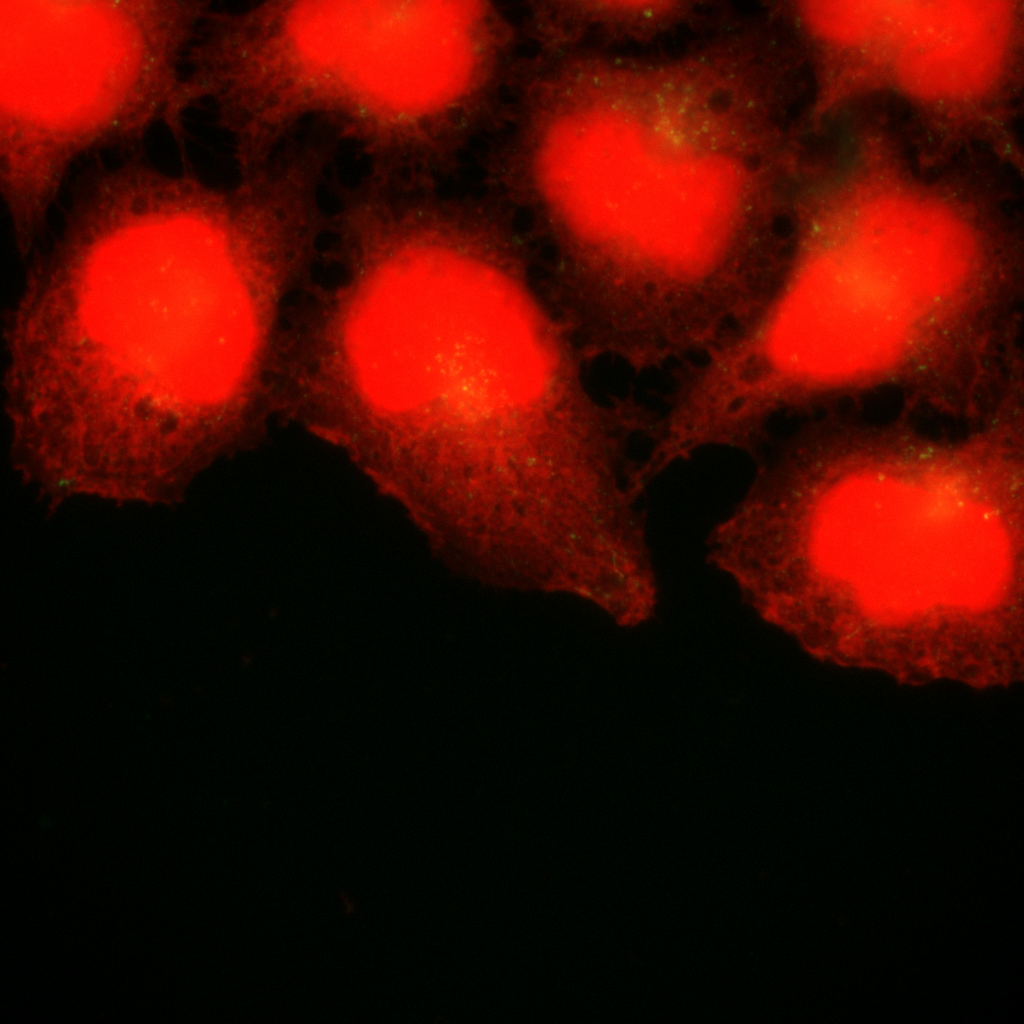

Supplement: S1 Dataset — Whole cell stain in red channel, endogenous SNX1 labelled in green channel. (ZIP) [file pone.0168294.s004.zip › Example Mock Images/Mock 3.tif]

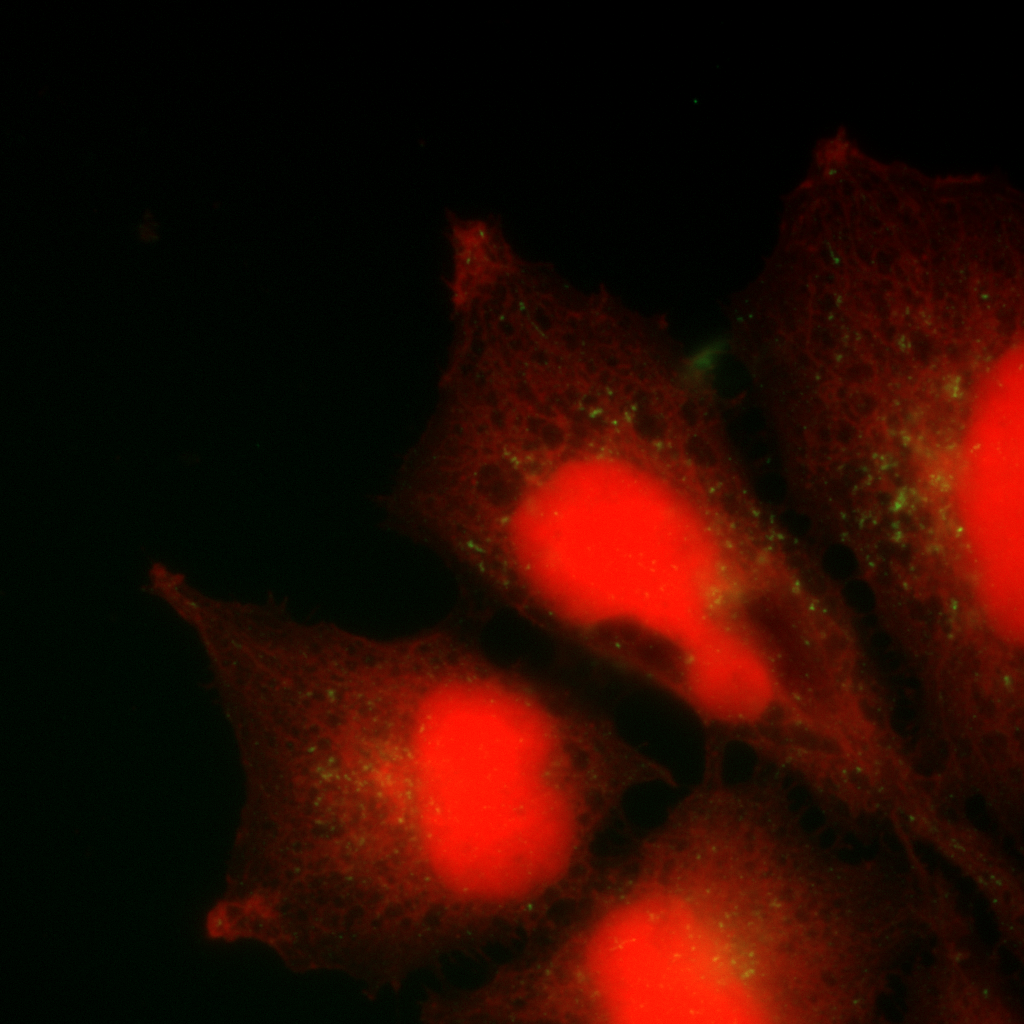

Supplement: S1 Dataset — Whole cell stain in red channel, endogenous SNX1 labelled in green channel. (ZIP) [file pone.0168294.s004.zip › Example Mock Images/Mock 30.tif]

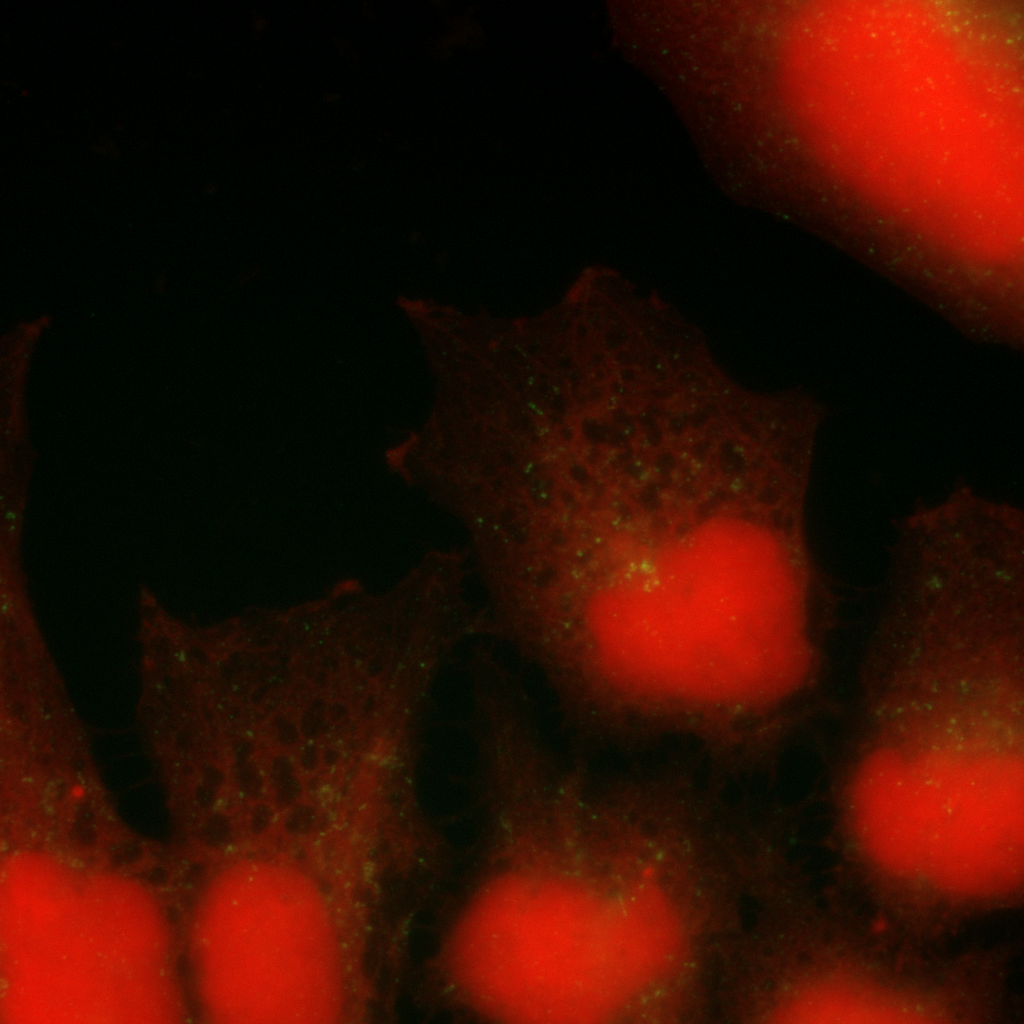

Supplement: S1 Dataset — Whole cell stain in red channel, endogenous SNX1 labelled in green channel. (ZIP) [file pone.0168294.s004.zip › Example Mock Images/Mock 31.tif]

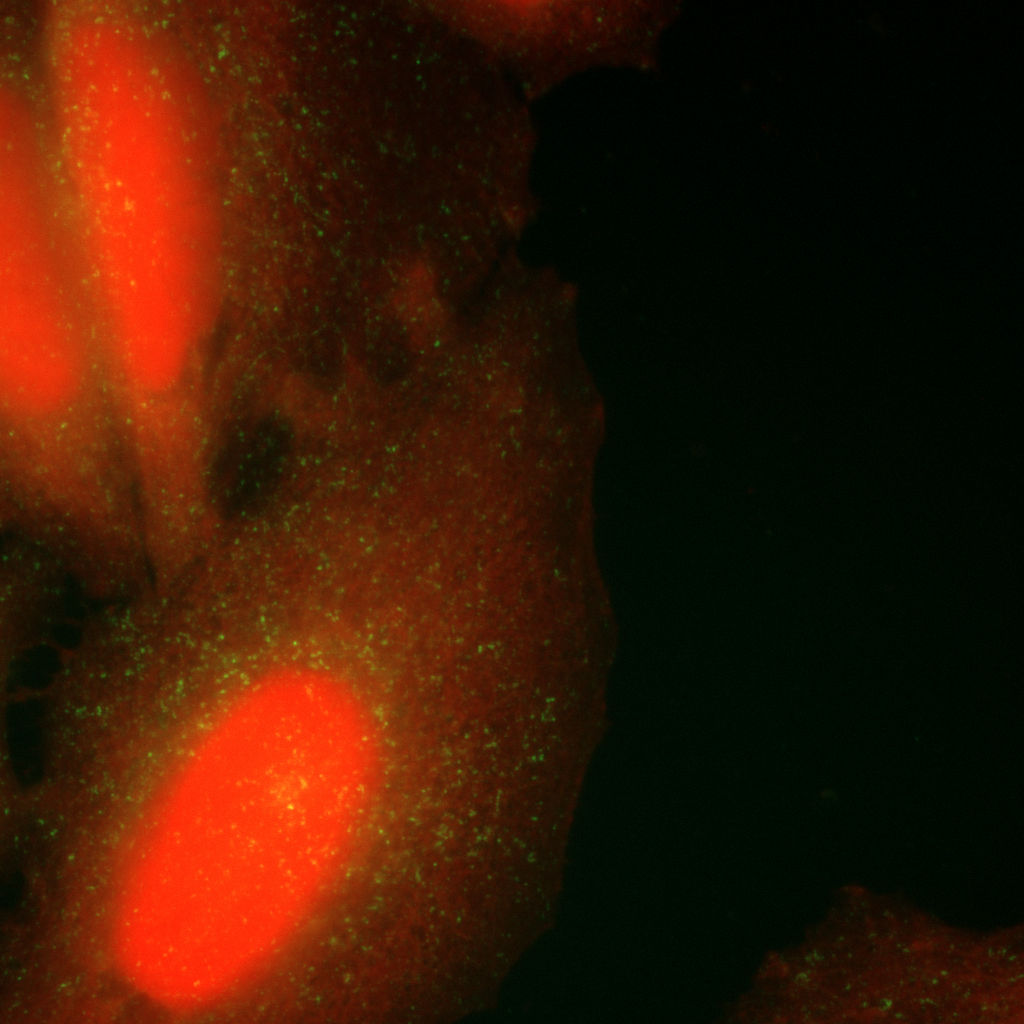

Supplement: S1 Dataset — Whole cell stain in red channel, endogenous SNX1 labelled in green channel. (ZIP) [file pone.0168294.s004.zip › Example Mock Images/Mock 32.tif]

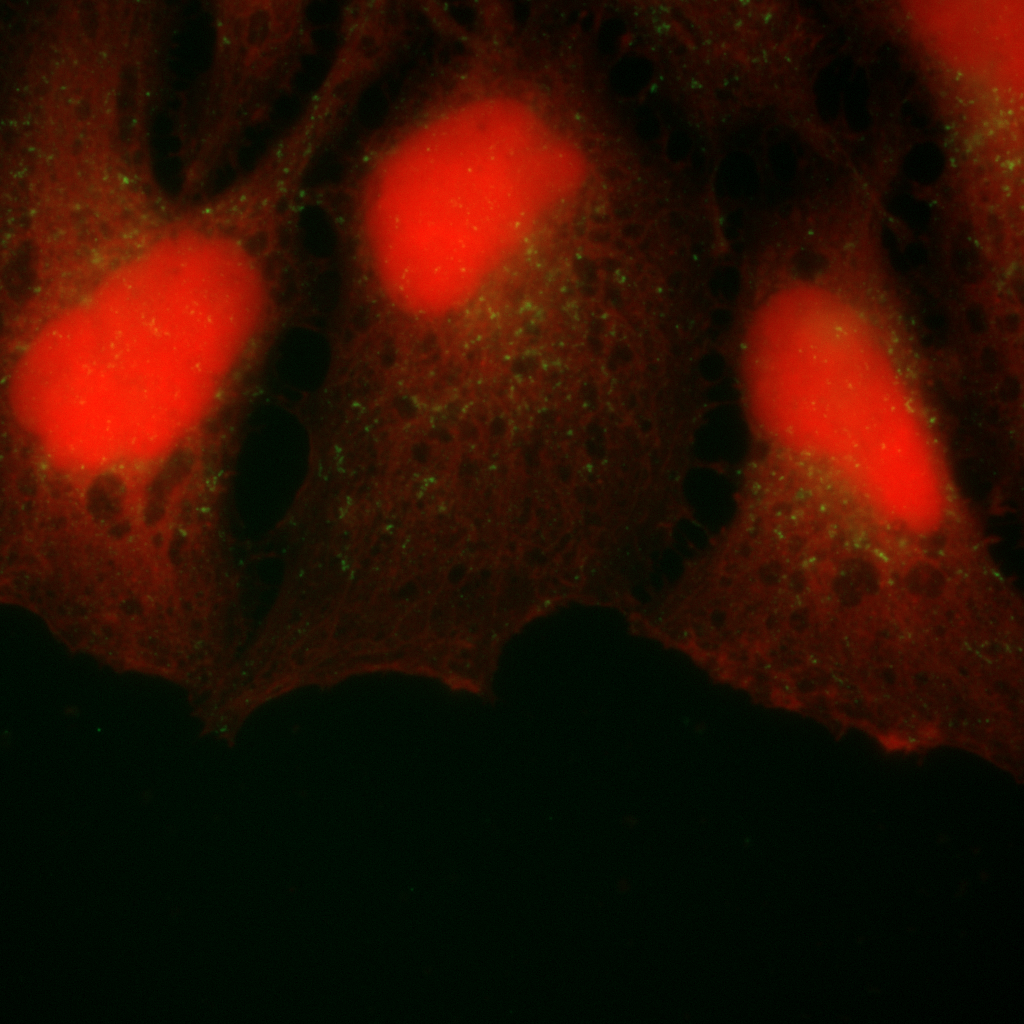

Supplement: S1 Dataset — Whole cell stain in red channel, endogenous SNX1 labelled in green channel. (ZIP) [file pone.0168294.s004.zip › Example Mock Images/Mock 33.tif]

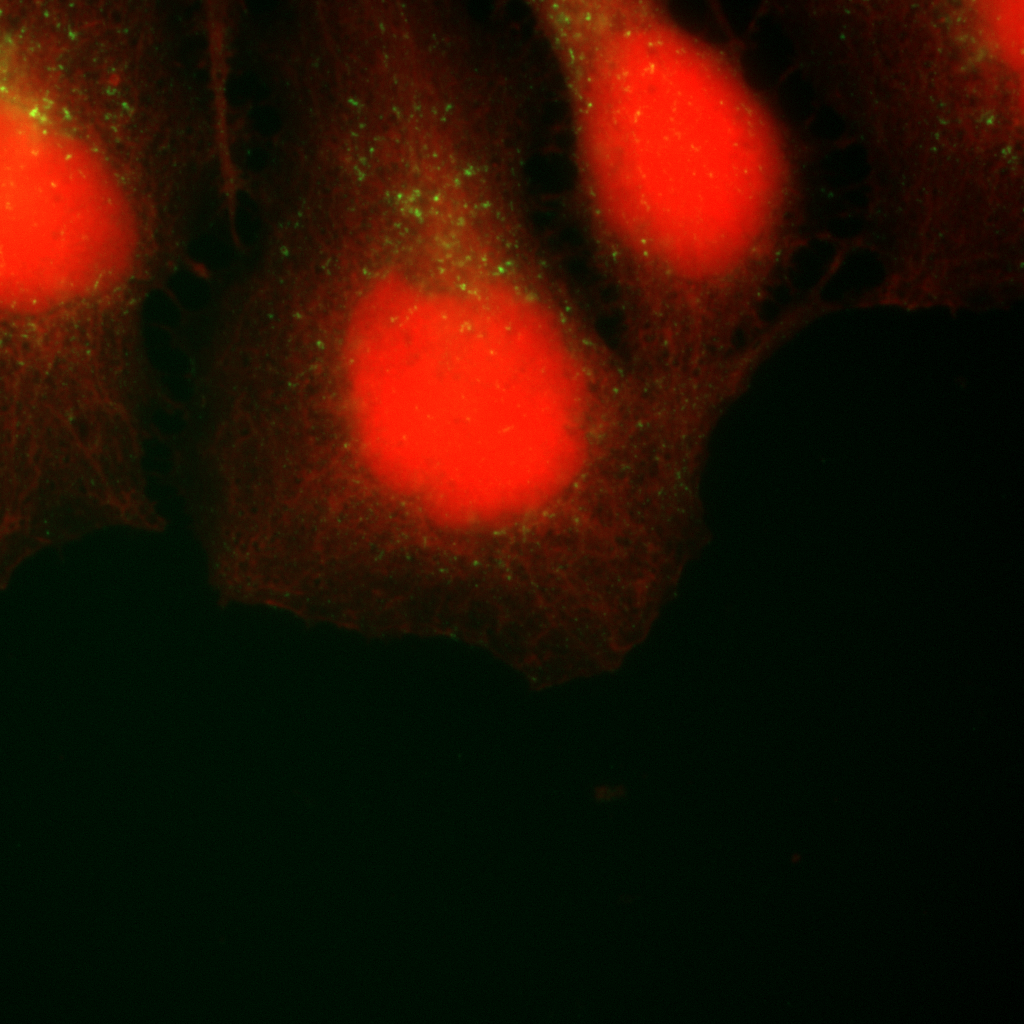

Supplement: S1 Dataset — Whole cell stain in red channel, endogenous SNX1 labelled in green channel. (ZIP) [file pone.0168294.s004.zip › Example Mock Images/Mock 34.tif]

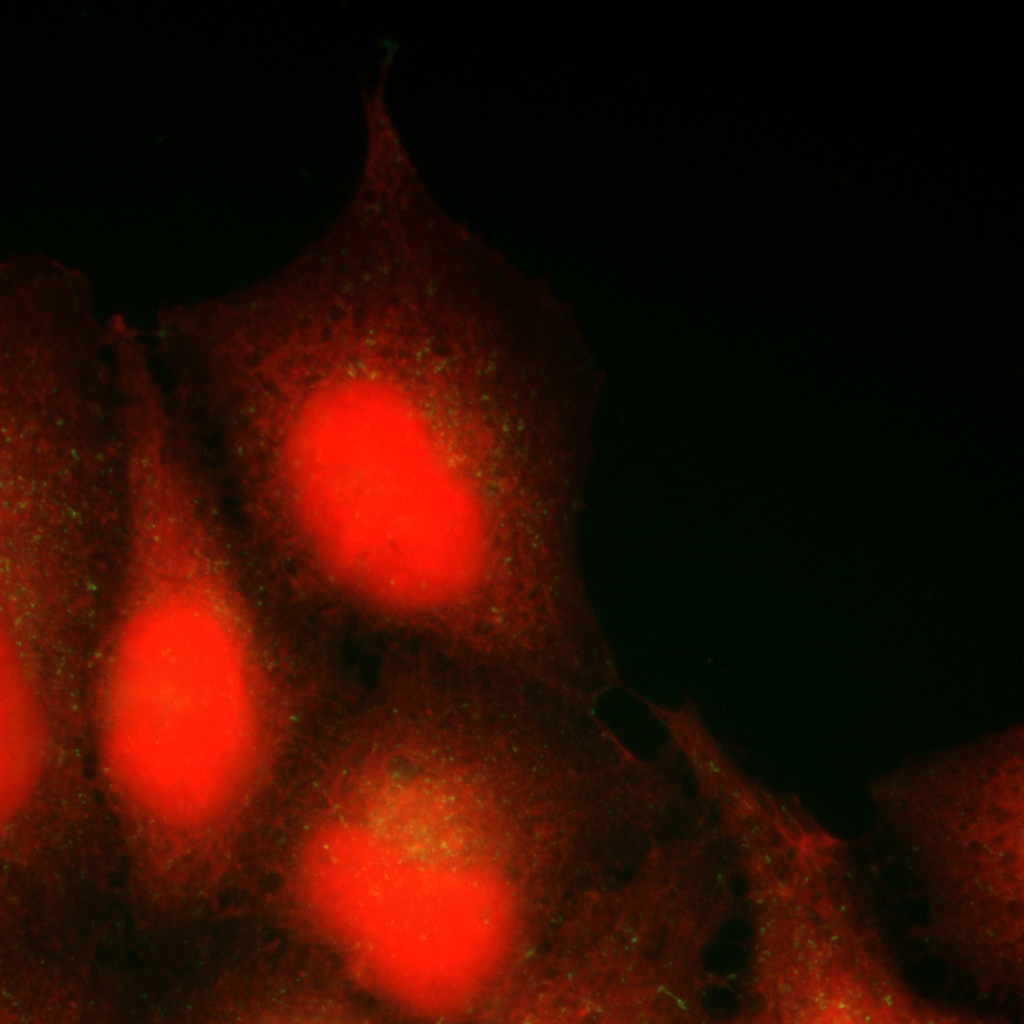

Supplement: S1 Dataset — Whole cell stain in red channel, endogenous SNX1 labelled in green channel. (ZIP) [file pone.0168294.s004.zip › Example Mock Images/Mock 35.tif]

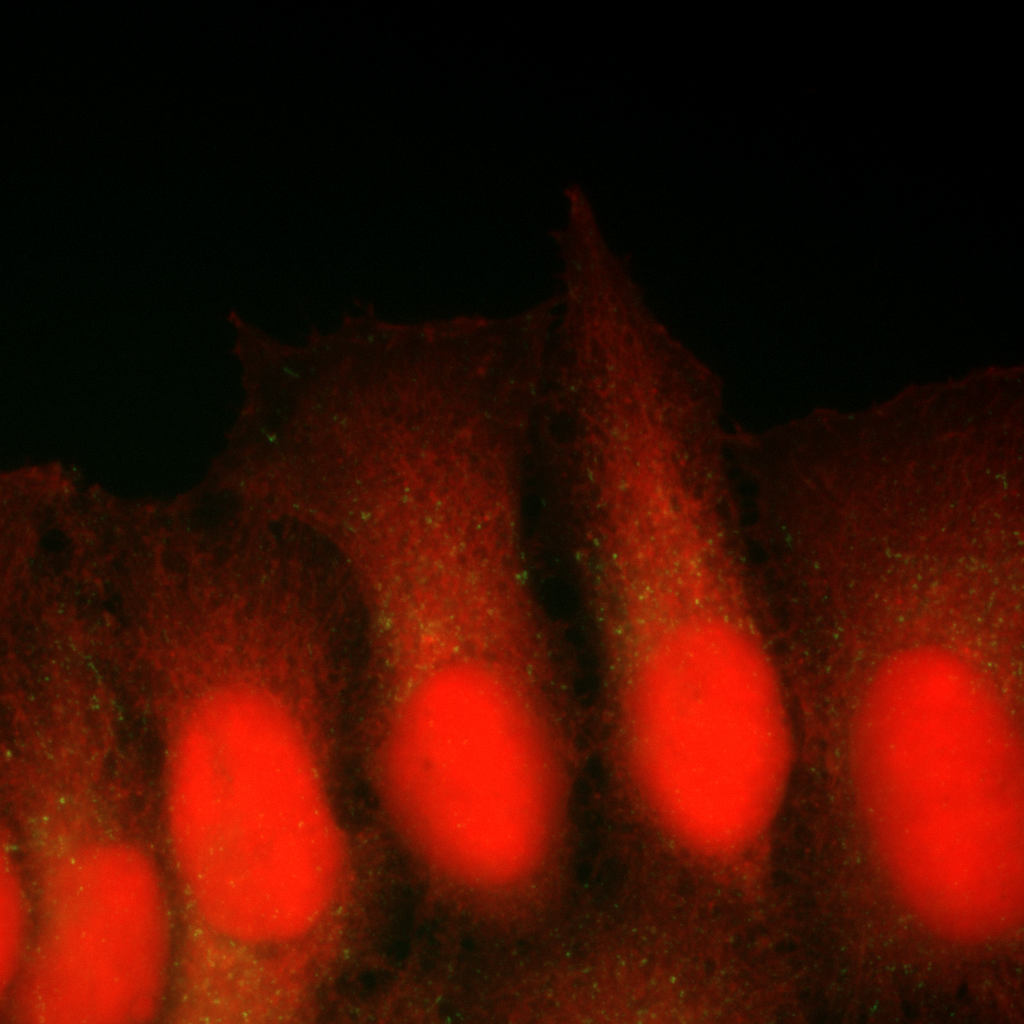

Supplement: S1 Dataset — Whole cell stain in red channel, endogenous SNX1 labelled in green channel. (ZIP) [file pone.0168294.s004.zip › Example Mock Images/Mock 36.tif]

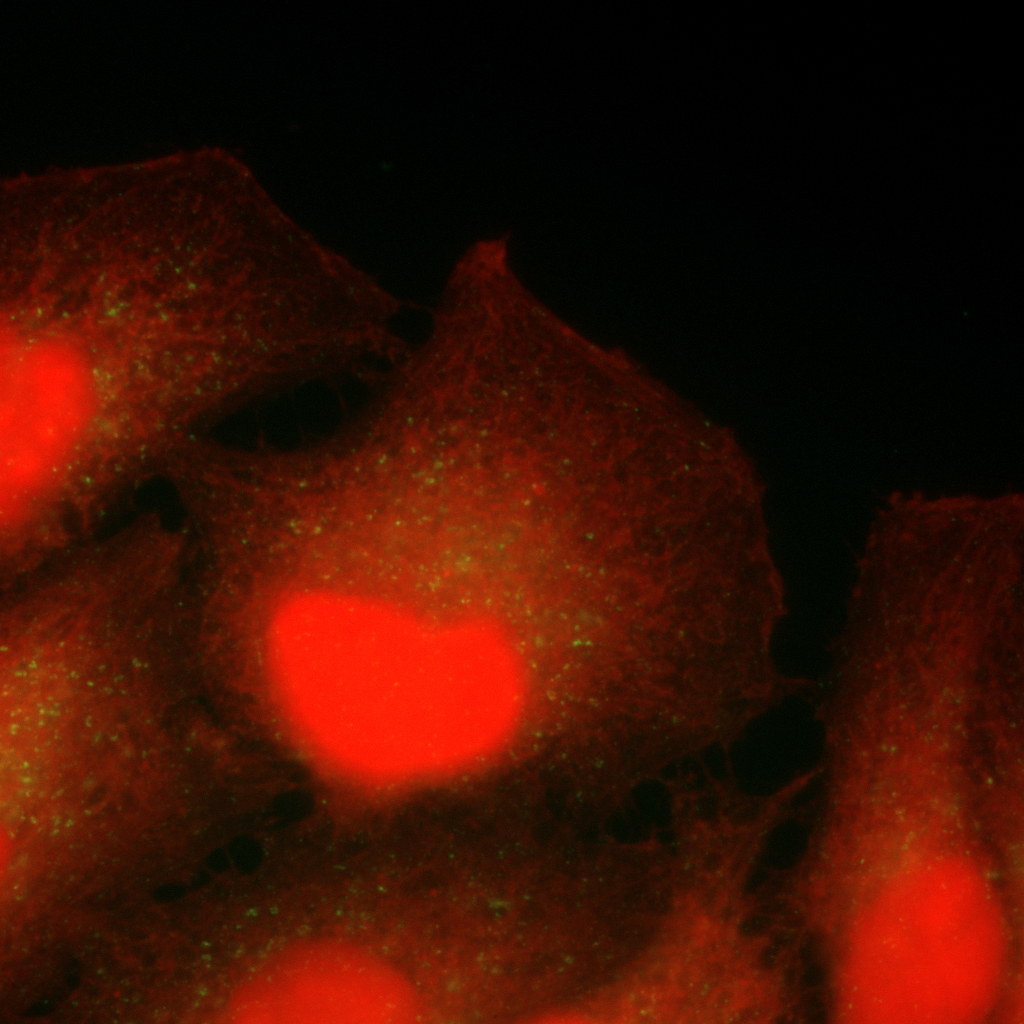

Supplement: S1 Dataset — Whole cell stain in red channel, endogenous SNX1 labelled in green channel. (ZIP) [file pone.0168294.s004.zip › Example Mock Images/Mock 37.tif]

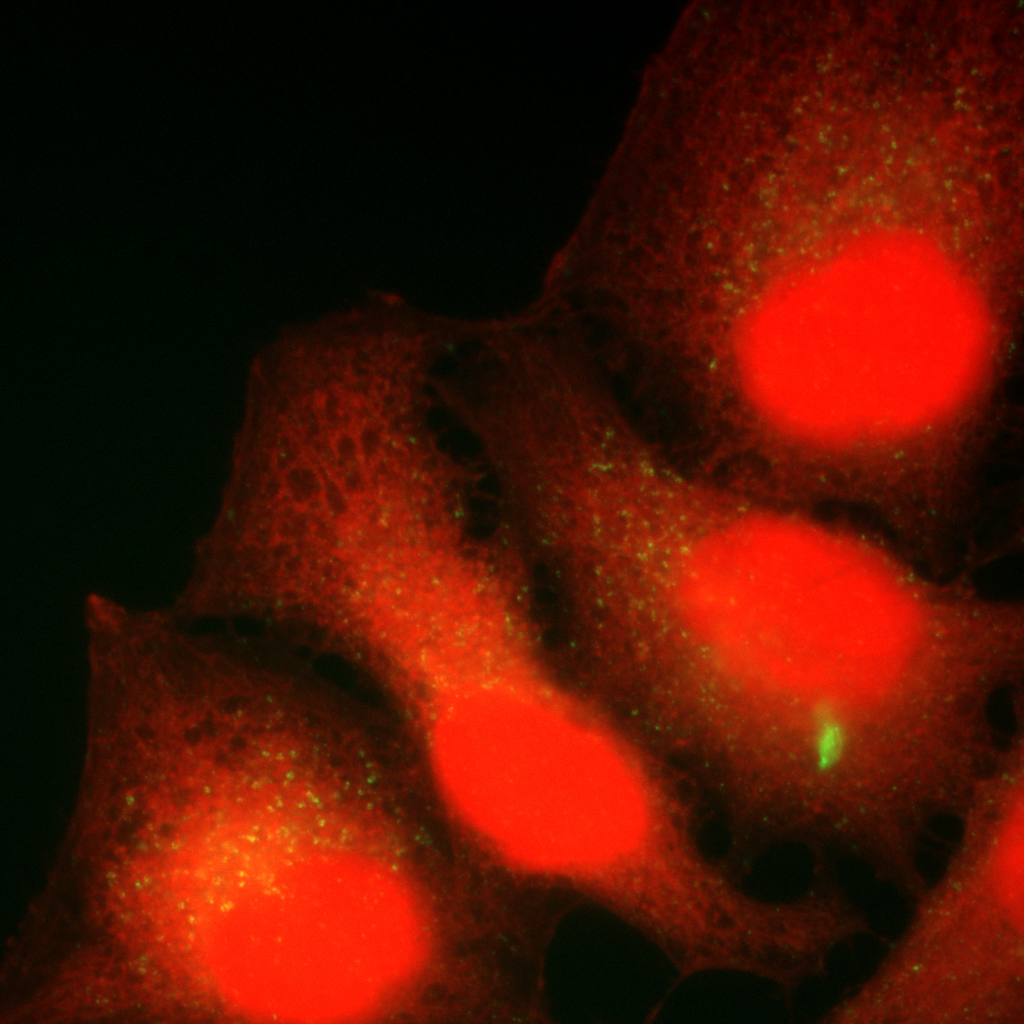

Supplement: S1 Dataset — Whole cell stain in red channel, endogenous SNX1 labelled in green channel. (ZIP) [file pone.0168294.s004.zip › Example Mock Images/Mock 38.tif]

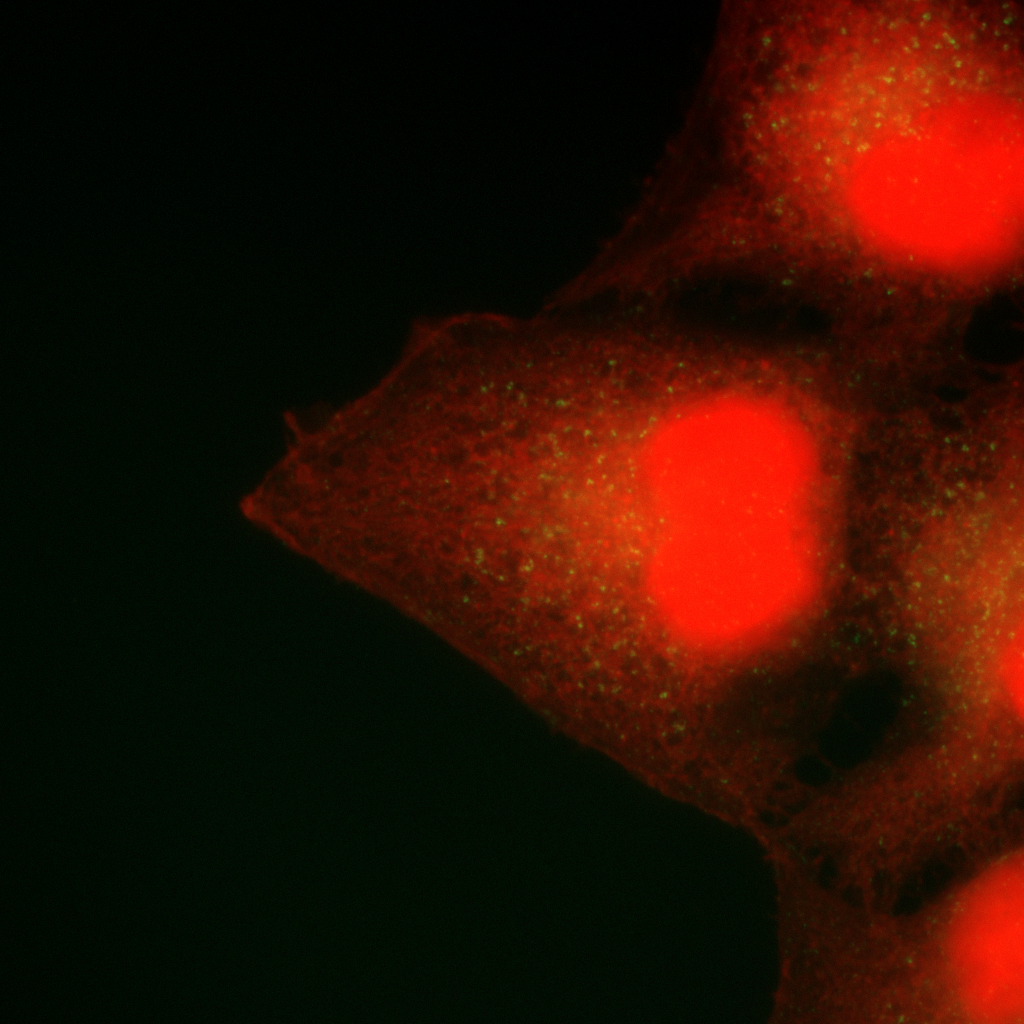

Supplement: S1 Dataset — Whole cell stain in red channel, endogenous SNX1 labelled in green channel. (ZIP) [file pone.0168294.s004.zip › Example Mock Images/Mock 39.tif]

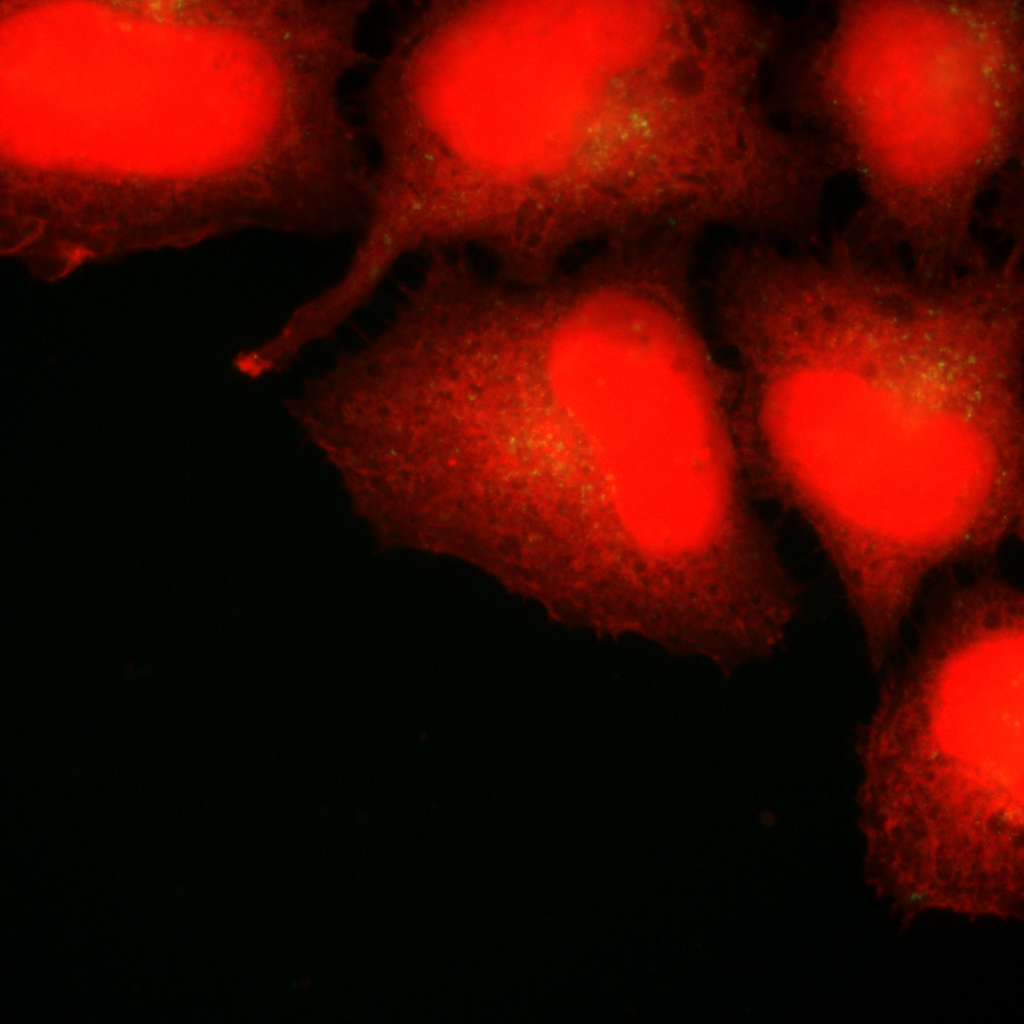

Supplement: S1 Dataset — Whole cell stain in red channel, endogenous SNX1 labelled in green channel. (ZIP) [file pone.0168294.s004.zip › Example Mock Images/Mock 4.tif]

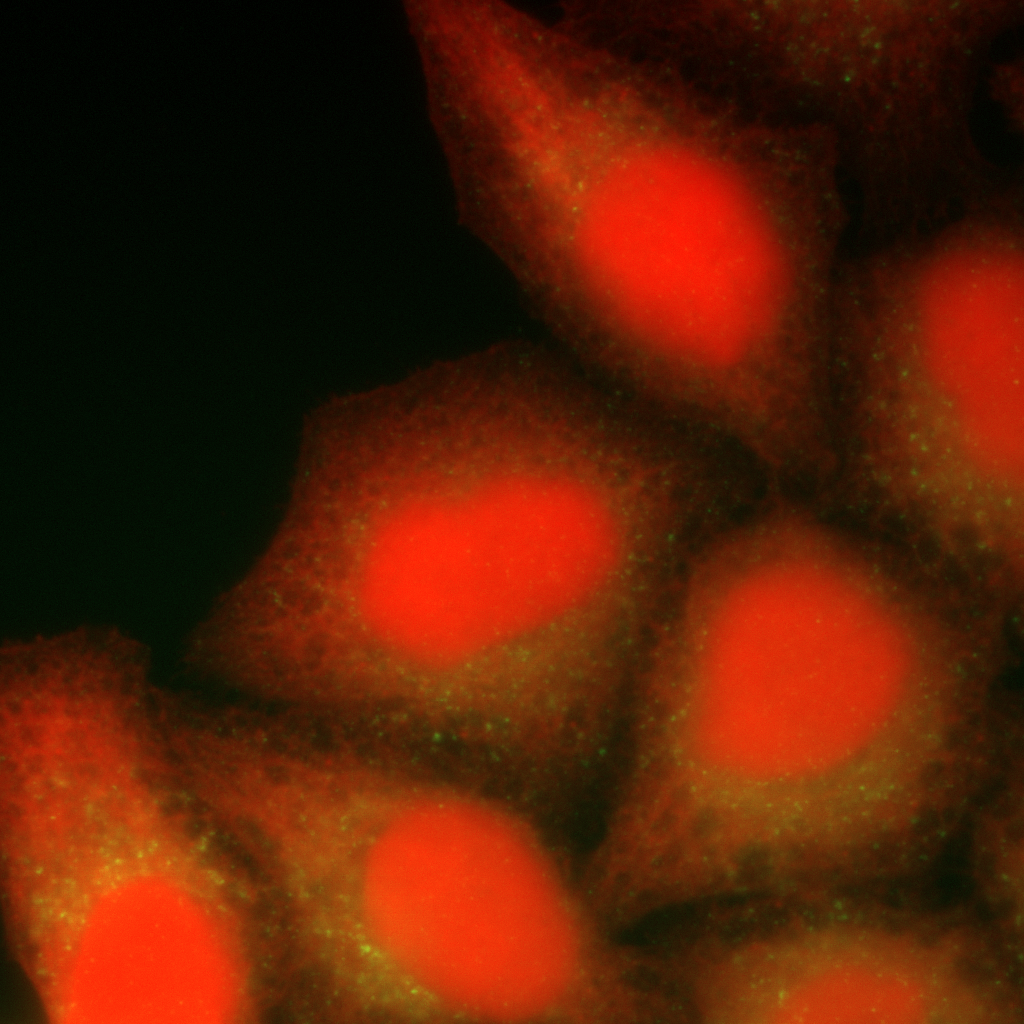

Supplement: S1 Dataset — Whole cell stain in red channel, endogenous SNX1 labelled in green channel. (ZIP) [file pone.0168294.s004.zip › Example Mock Images/Mock 40.tif]

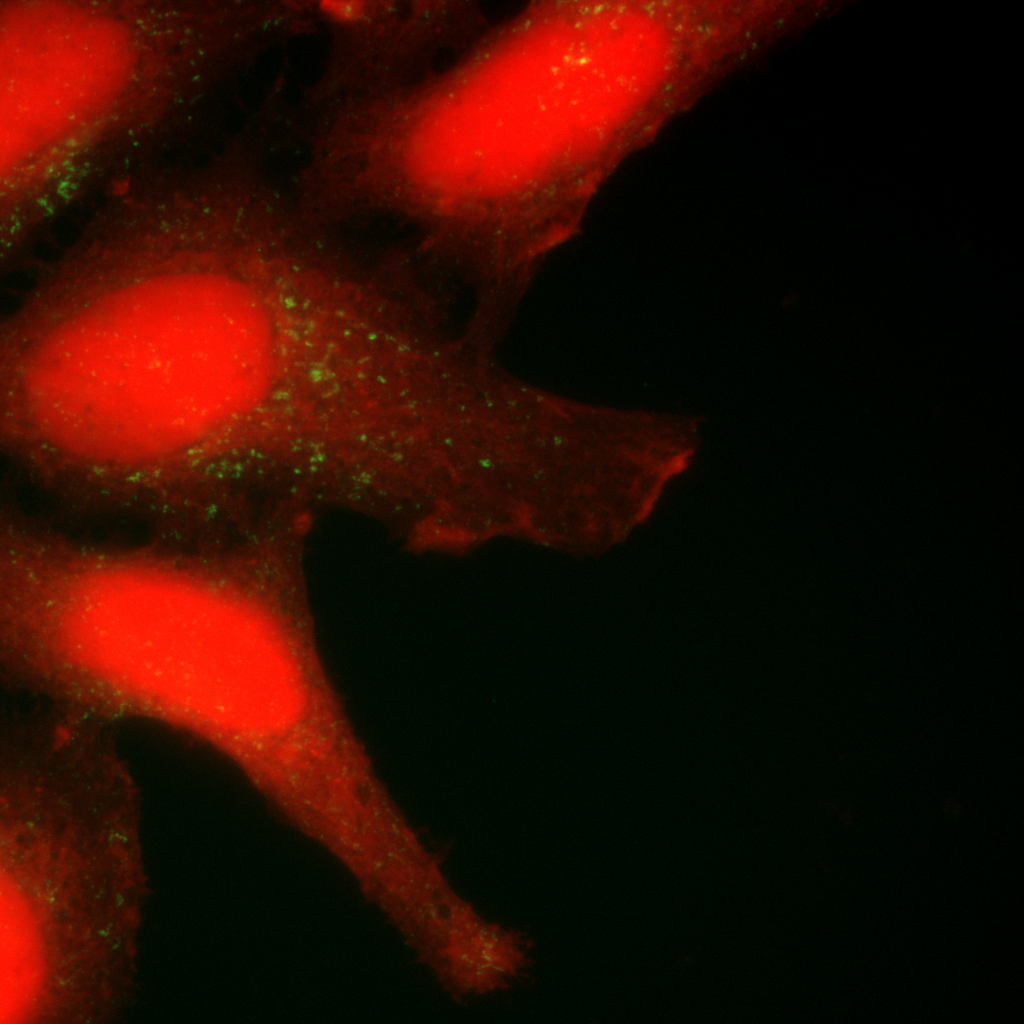

Supplement: S1 Dataset — Whole cell stain in red channel, endogenous SNX1 labelled in green channel. (ZIP) [file pone.0168294.s004.zip › Example Mock Images/Mock 41.tif]

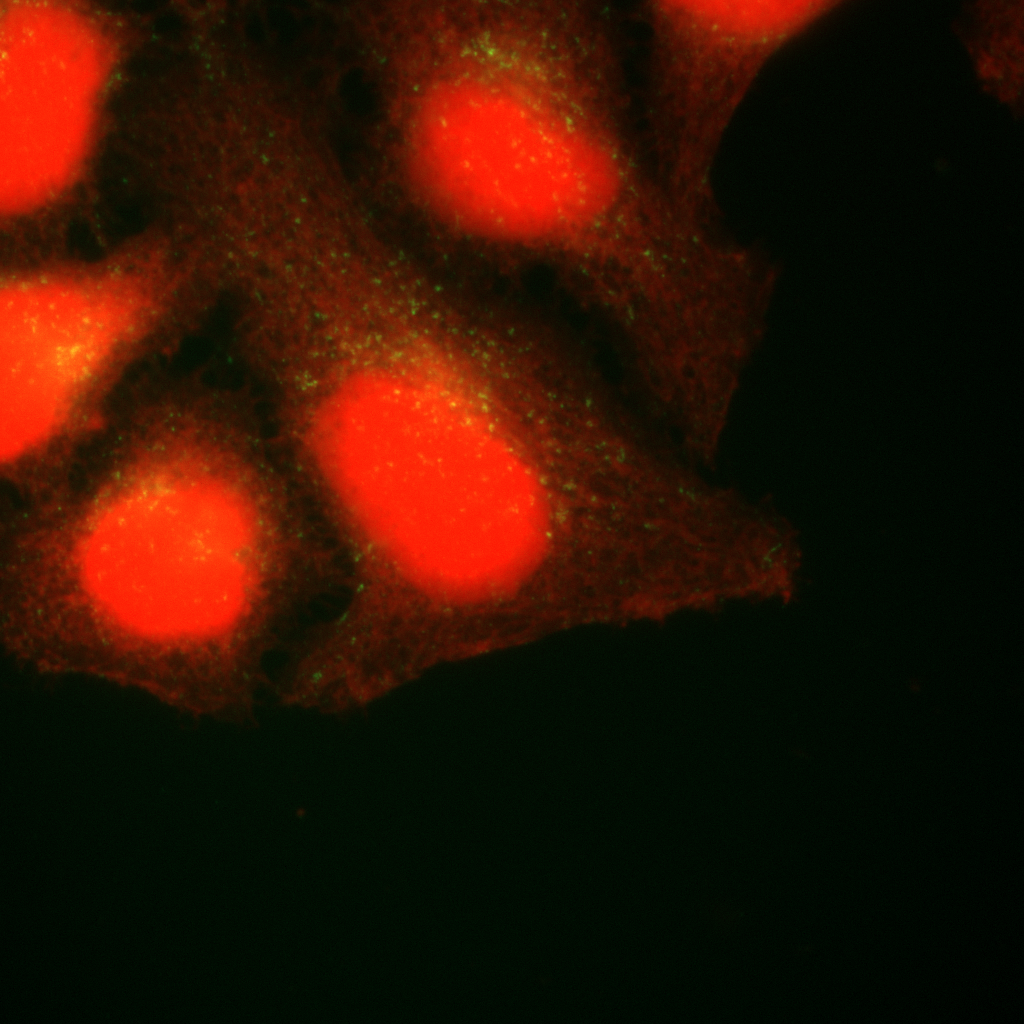

Supplement: S1 Dataset — Whole cell stain in red channel, endogenous SNX1 labelled in green channel. (ZIP) [file pone.0168294.s004.zip › Example Mock Images/Mock 42.tif]

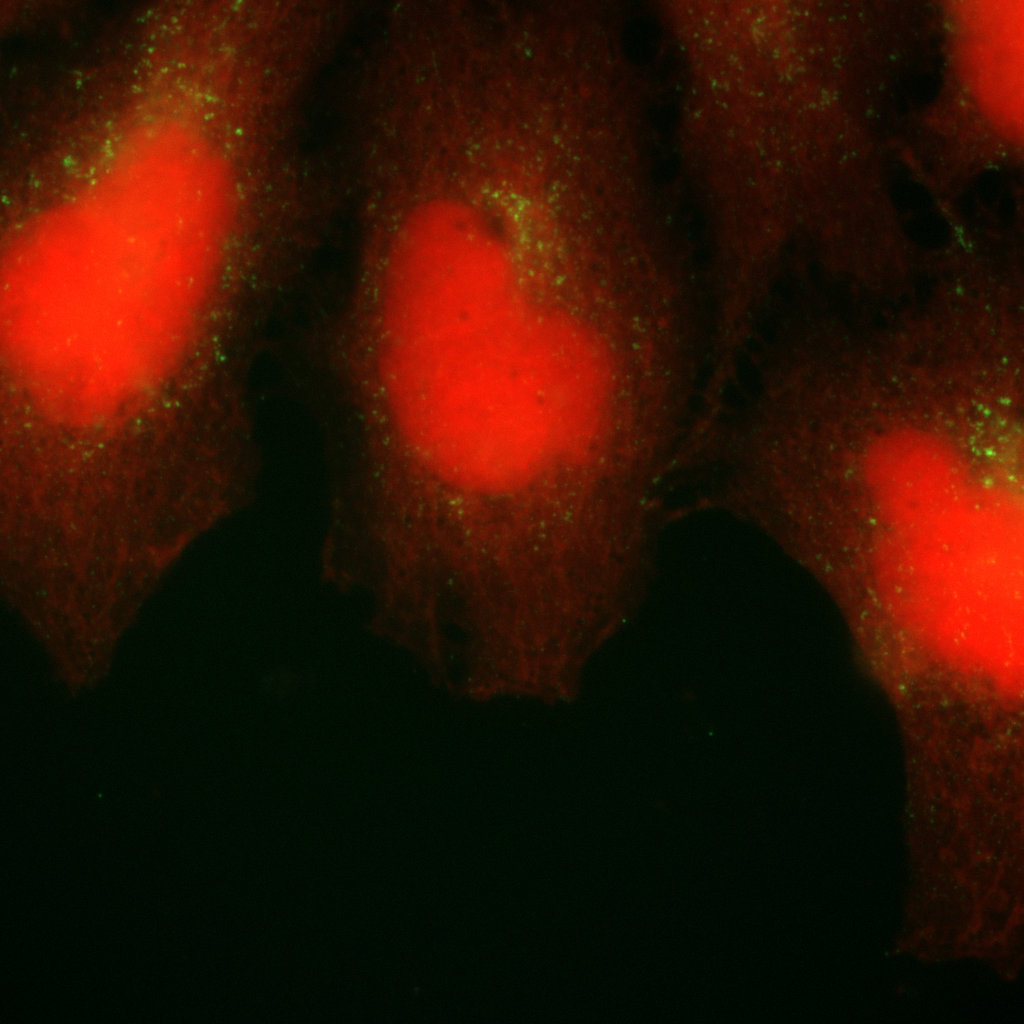

Supplement: S1 Dataset — Whole cell stain in red channel, endogenous SNX1 labelled in green channel. (ZIP) [file pone.0168294.s004.zip › Example Mock Images/Mock 43.tif]

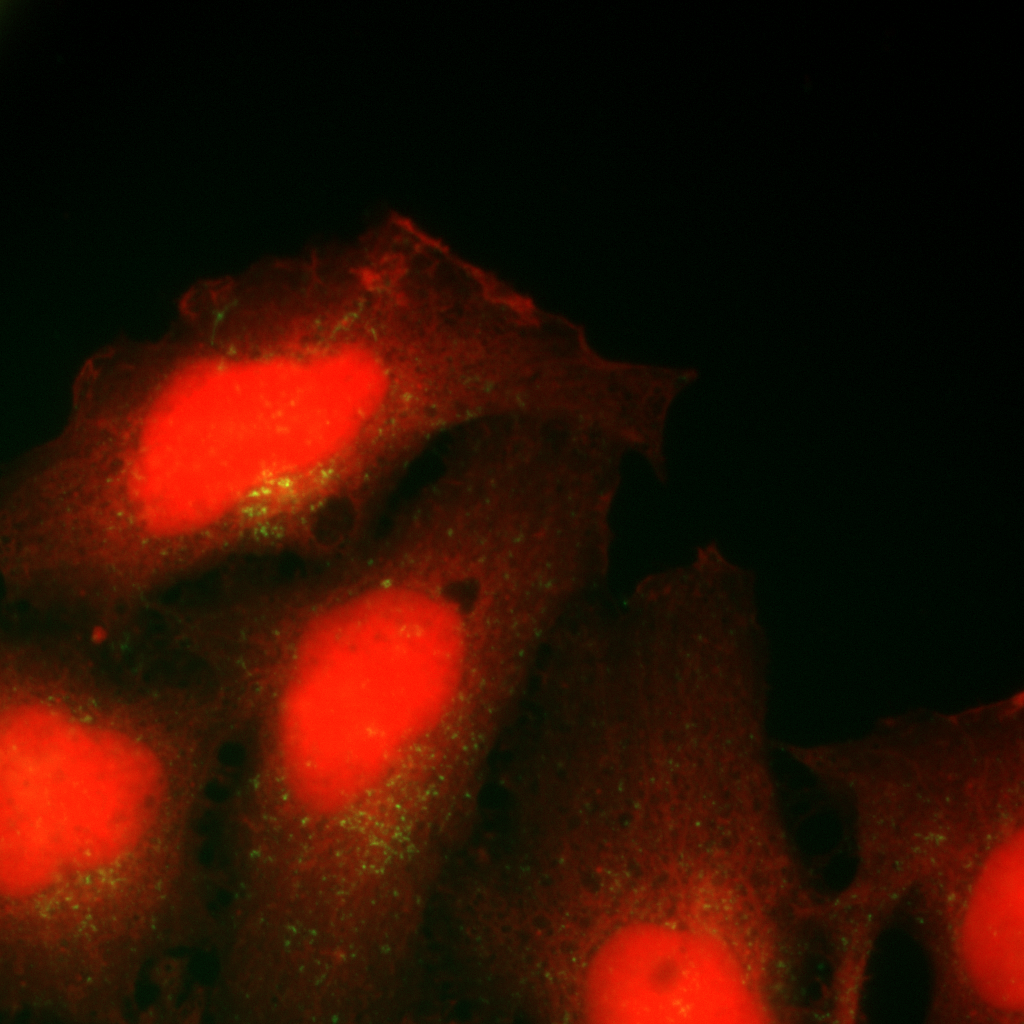

Supplement: S1 Dataset — Whole cell stain in red channel, endogenous SNX1 labelled in green channel. (ZIP) [file pone.0168294.s004.zip › Example Mock Images/Mock 44.tif]

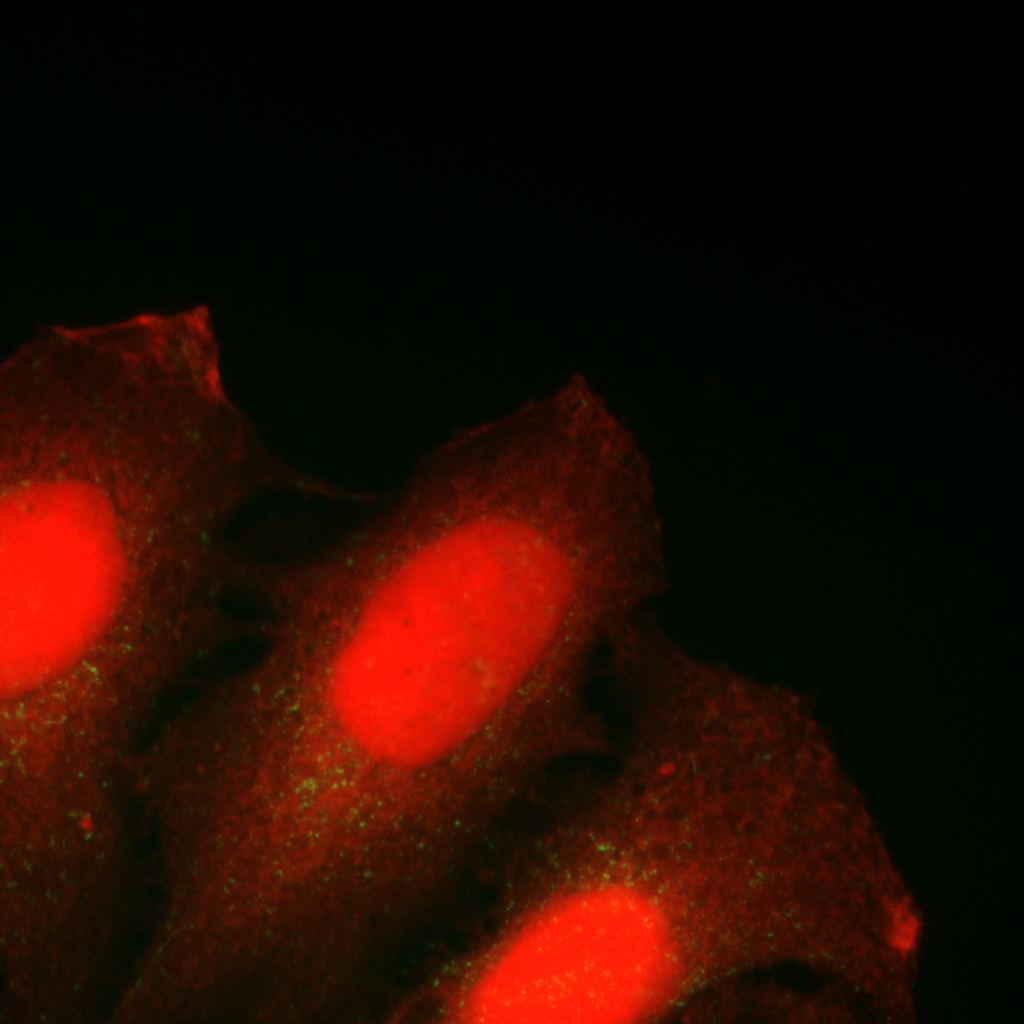

Supplement: S1 Dataset — Whole cell stain in red channel, endogenous SNX1 labelled in green channel. (ZIP) [file pone.0168294.s004.zip › Example Mock Images/Mock 45.tif]

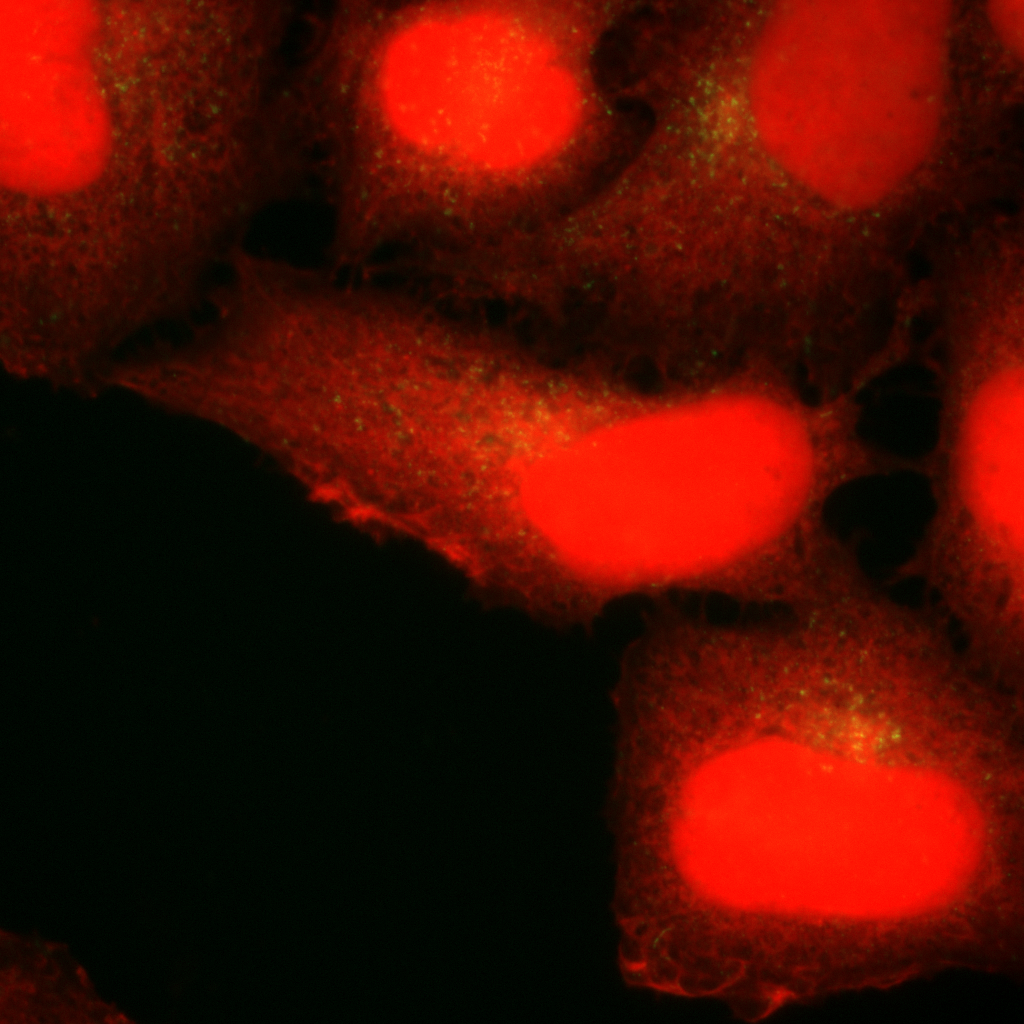

Supplement: S1 Dataset — Whole cell stain in red channel, endogenous SNX1 labelled in green channel. (ZIP) [file pone.0168294.s004.zip › Example Mock Images/Mock 5.tif]

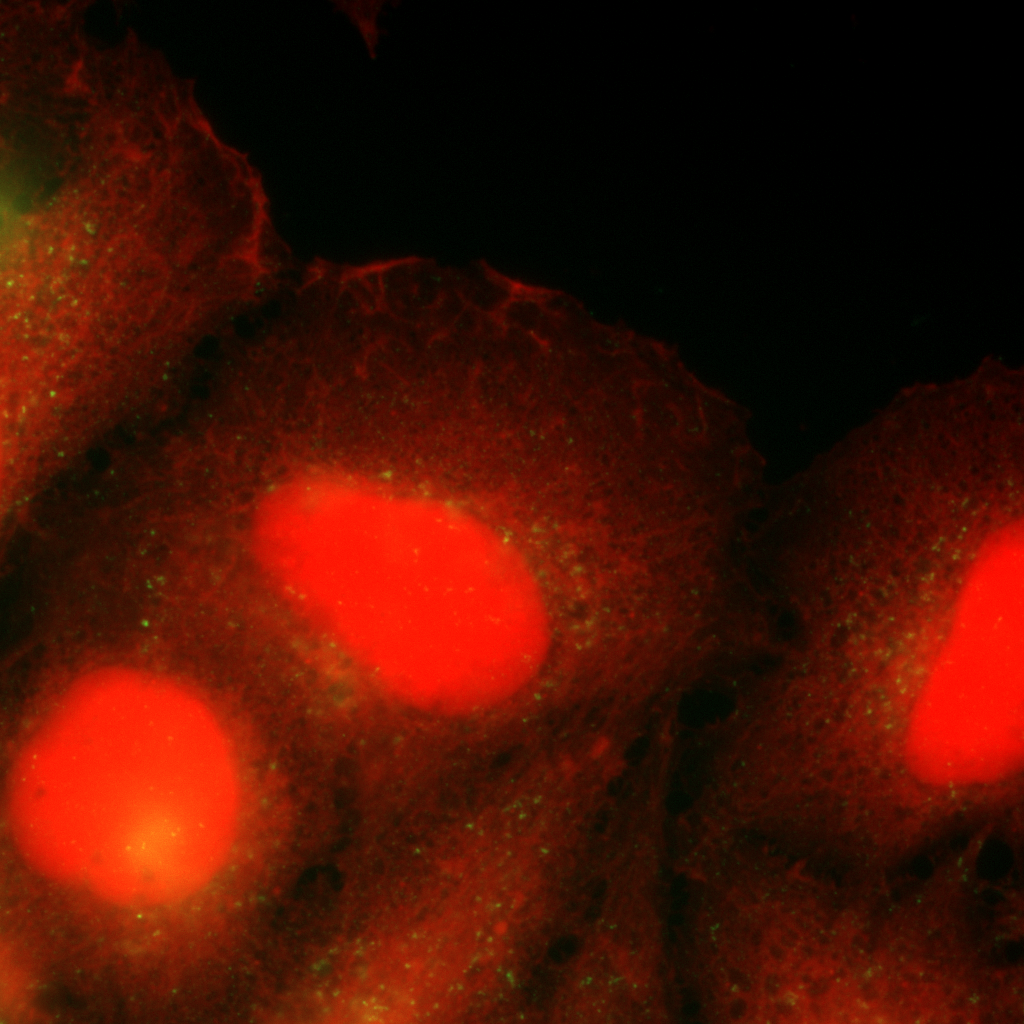

Supplement: S1 Dataset — Whole cell stain in red channel, endogenous SNX1 labelled in green channel. (ZIP) [file pone.0168294.s004.zip › Example Mock Images/Mock 6.tif]

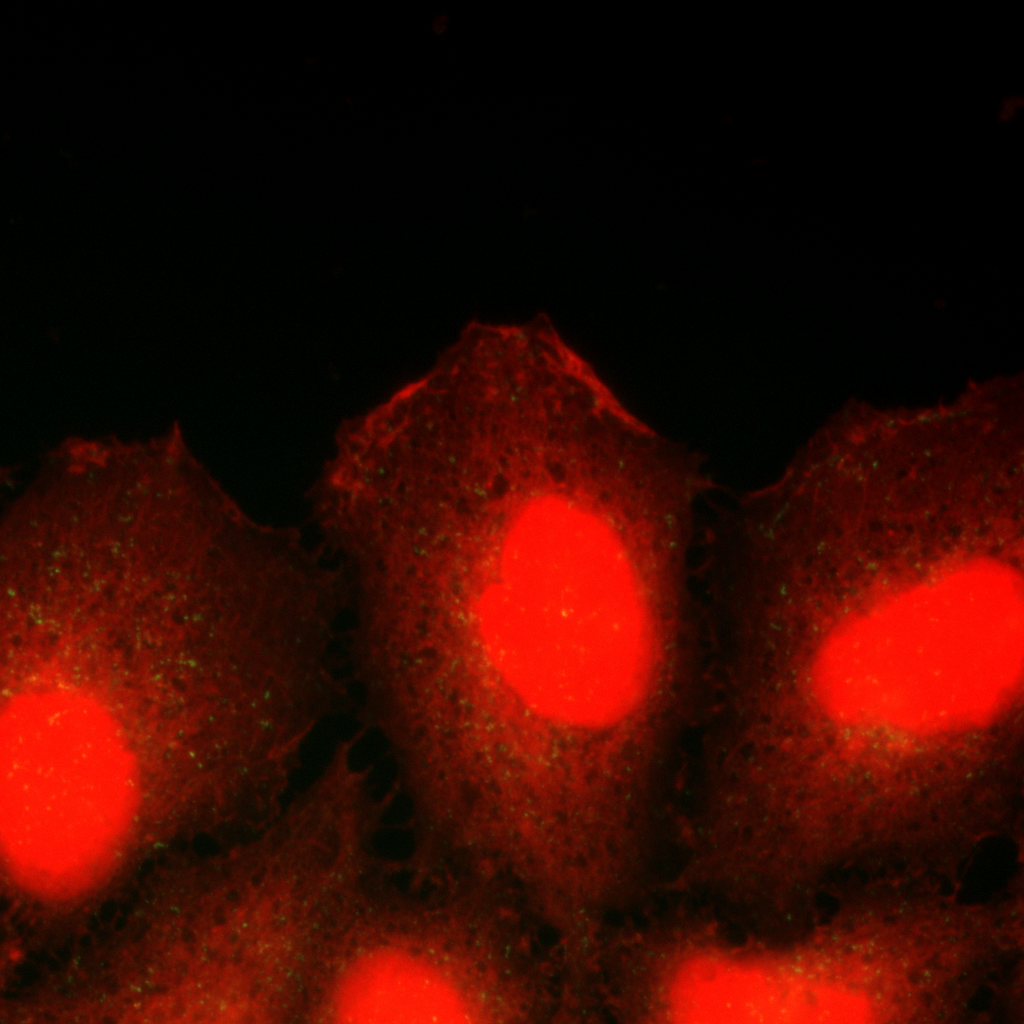

Supplement: S1 Dataset — Whole cell stain in red channel, endogenous SNX1 labelled in green channel. (ZIP) [file pone.0168294.s004.zip › Example Mock Images/Mock 7.tif]

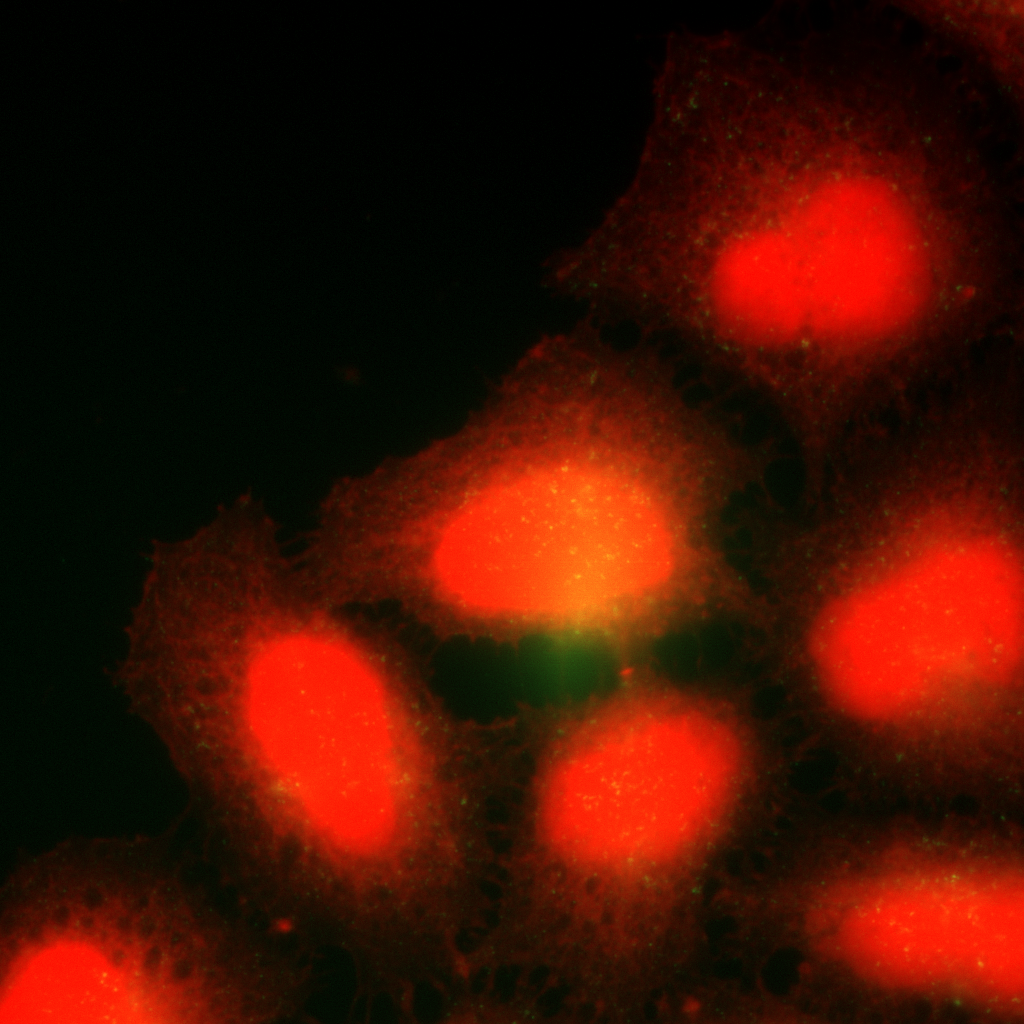

Supplement: S1 Dataset — Whole cell stain in red channel, endogenous SNX1 labelled in green channel. (ZIP) [file pone.0168294.s004.zip › Example Mock Images/Mock 8.tif]

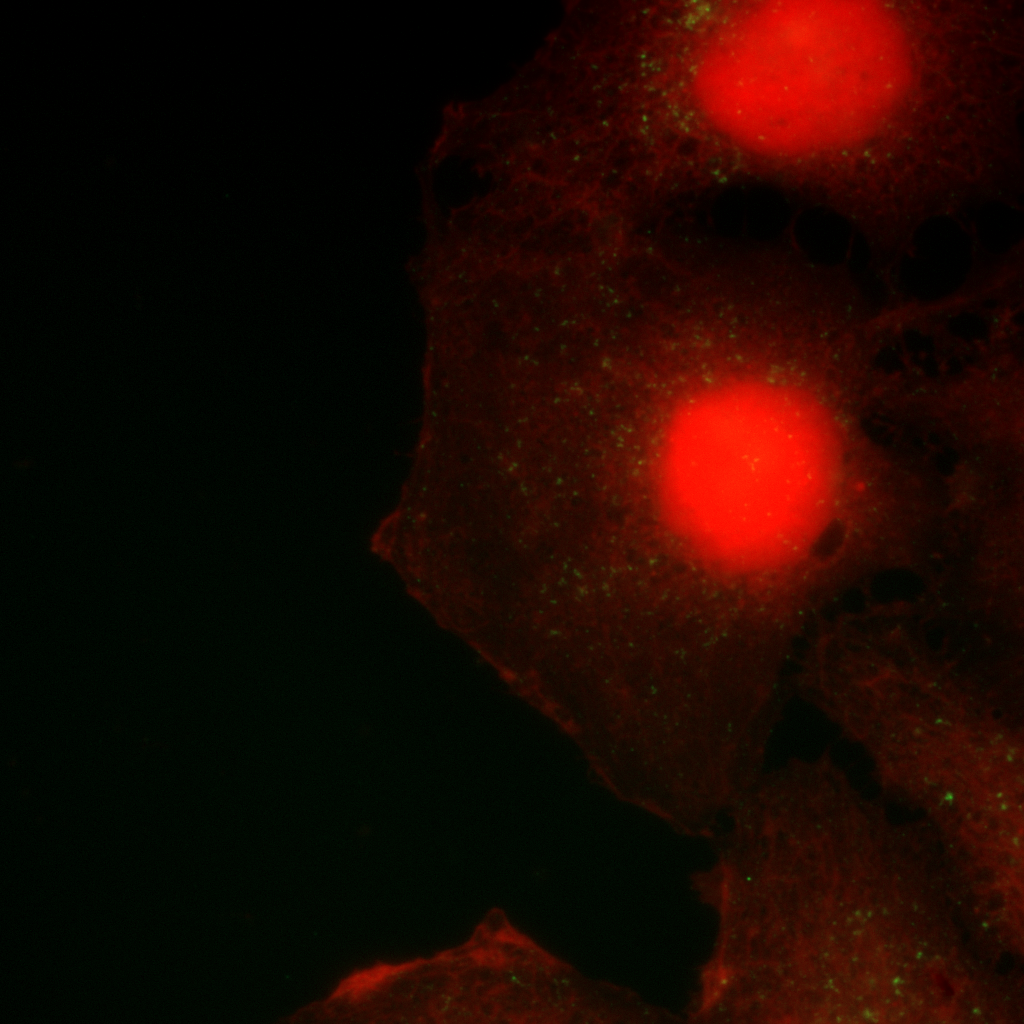

Supplement: S1 Dataset — Whole cell stain in red channel, endogenous SNX1 labelled in green channel. (ZIP) [file pone.0168294.s004.zip › Example Mock Images/Mock 9.tif]

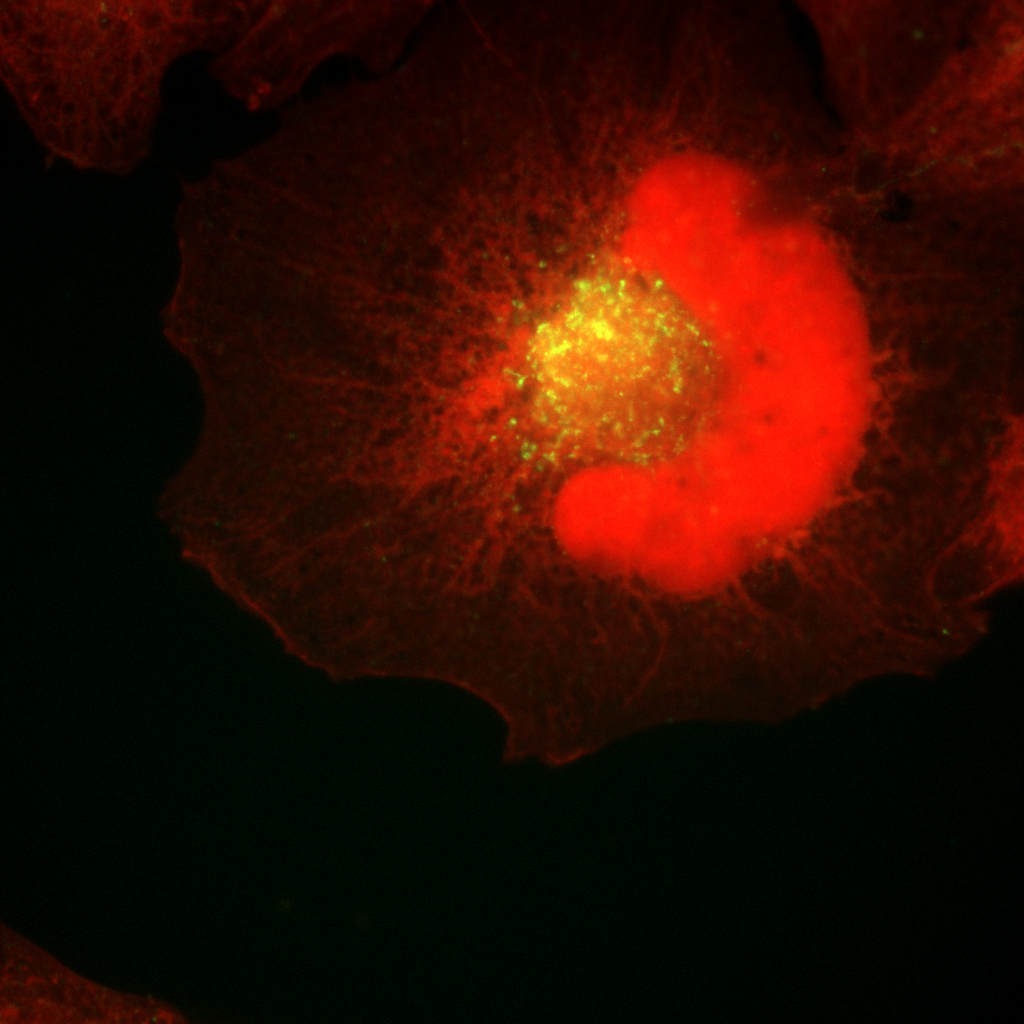

Supplement: S2 Dataset — Whole cell stain in red channel, endogenous SNX1 labelled in green channel. (ZIP) [file pone.0168294.s005.zip › Example Spastin depletion Images/Spastin knockdown 1.tif]

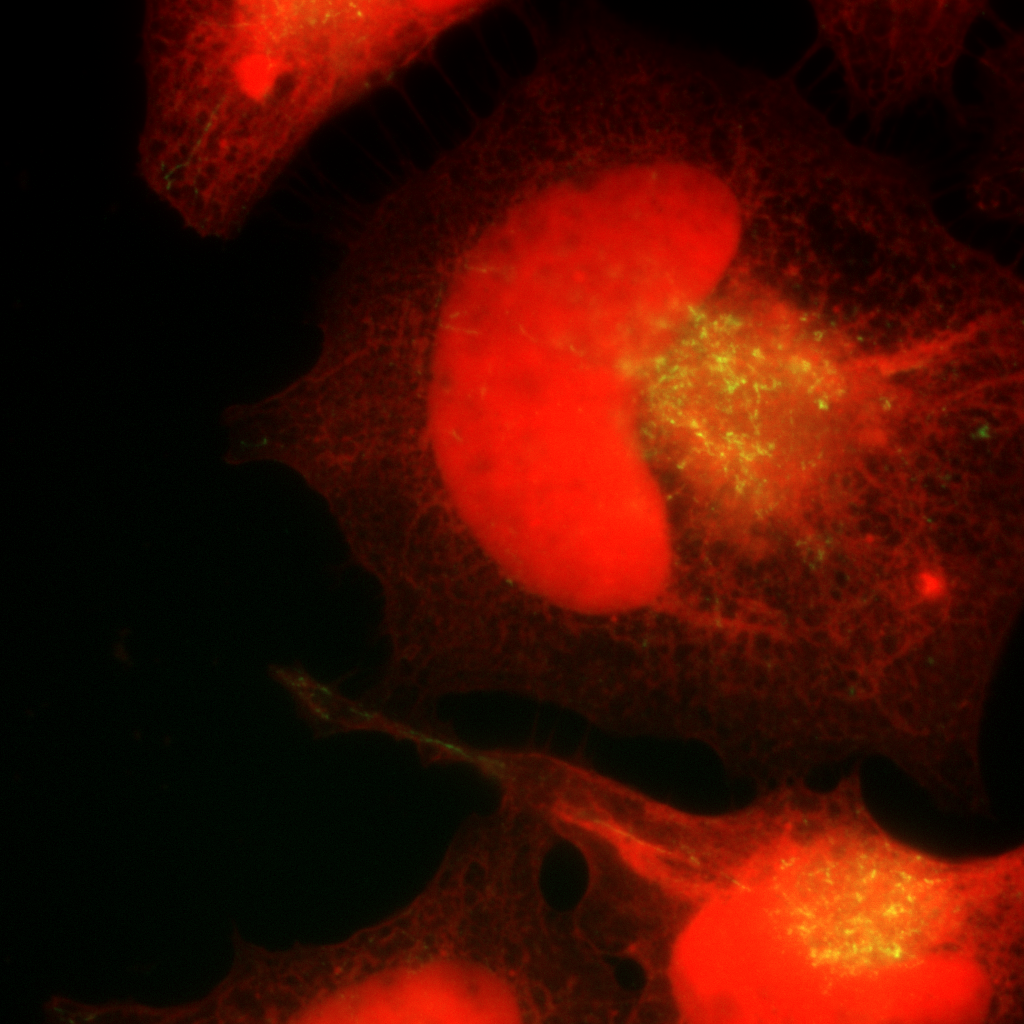

Supplement: S2 Dataset — Whole cell stain in red channel, endogenous SNX1 labelled in green channel. (ZIP) [file pone.0168294.s005.zip › Example Spastin depletion Images/Spastin knockdown 10.tif]

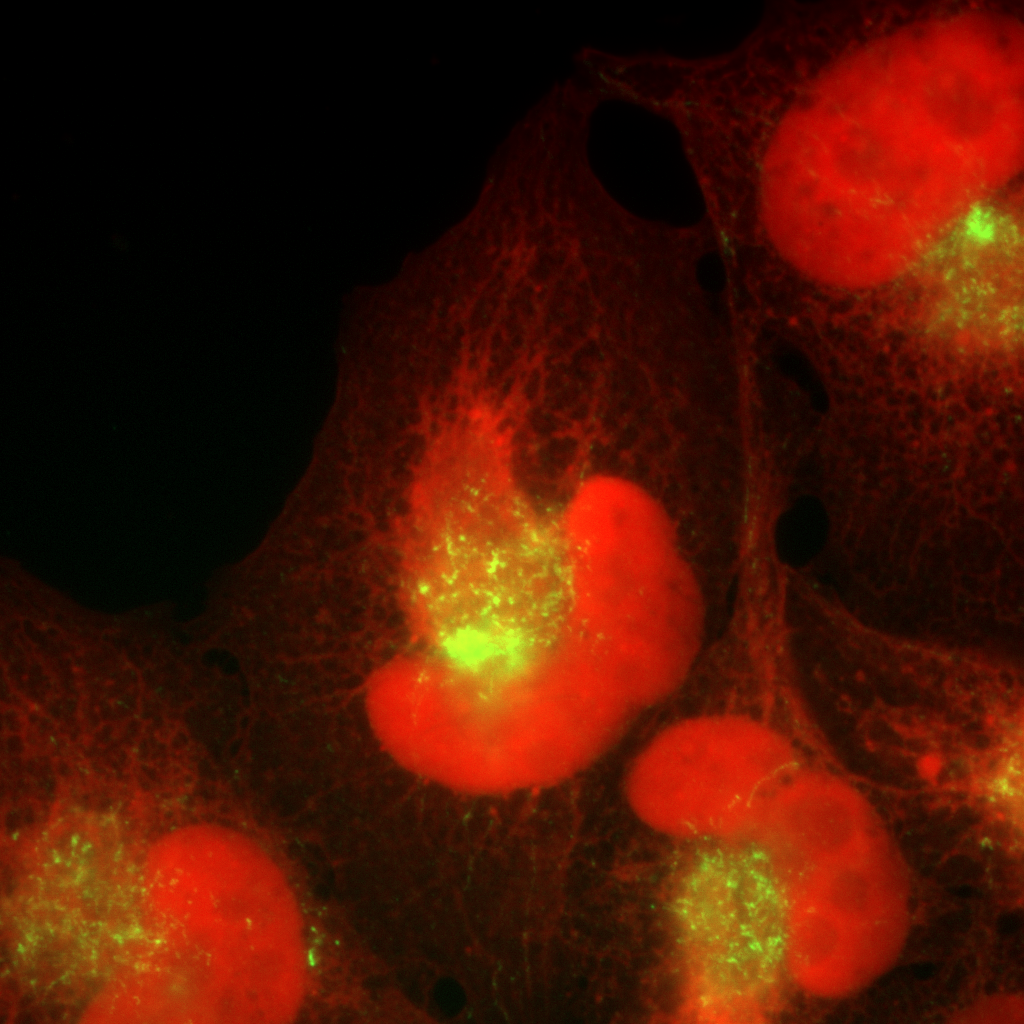

Supplement: S2 Dataset — Whole cell stain in red channel, endogenous SNX1 labelled in green channel. (ZIP) [file pone.0168294.s005.zip › Example Spastin depletion Images/Spastin knockdown 11.tif]

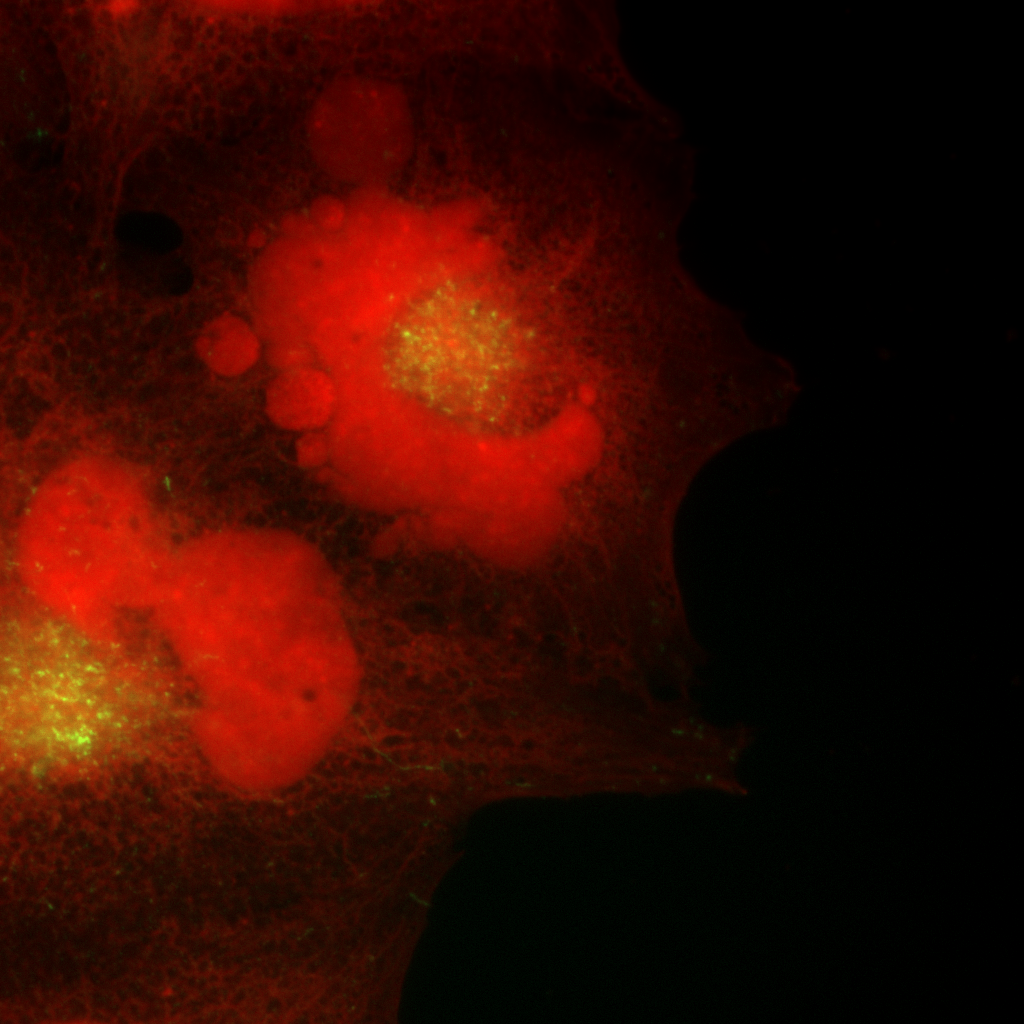

Supplement: S2 Dataset — Whole cell stain in red channel, endogenous SNX1 labelled in green channel. (ZIP) [file pone.0168294.s005.zip › Example Spastin depletion Images/Spastin knockdown 12.tif]

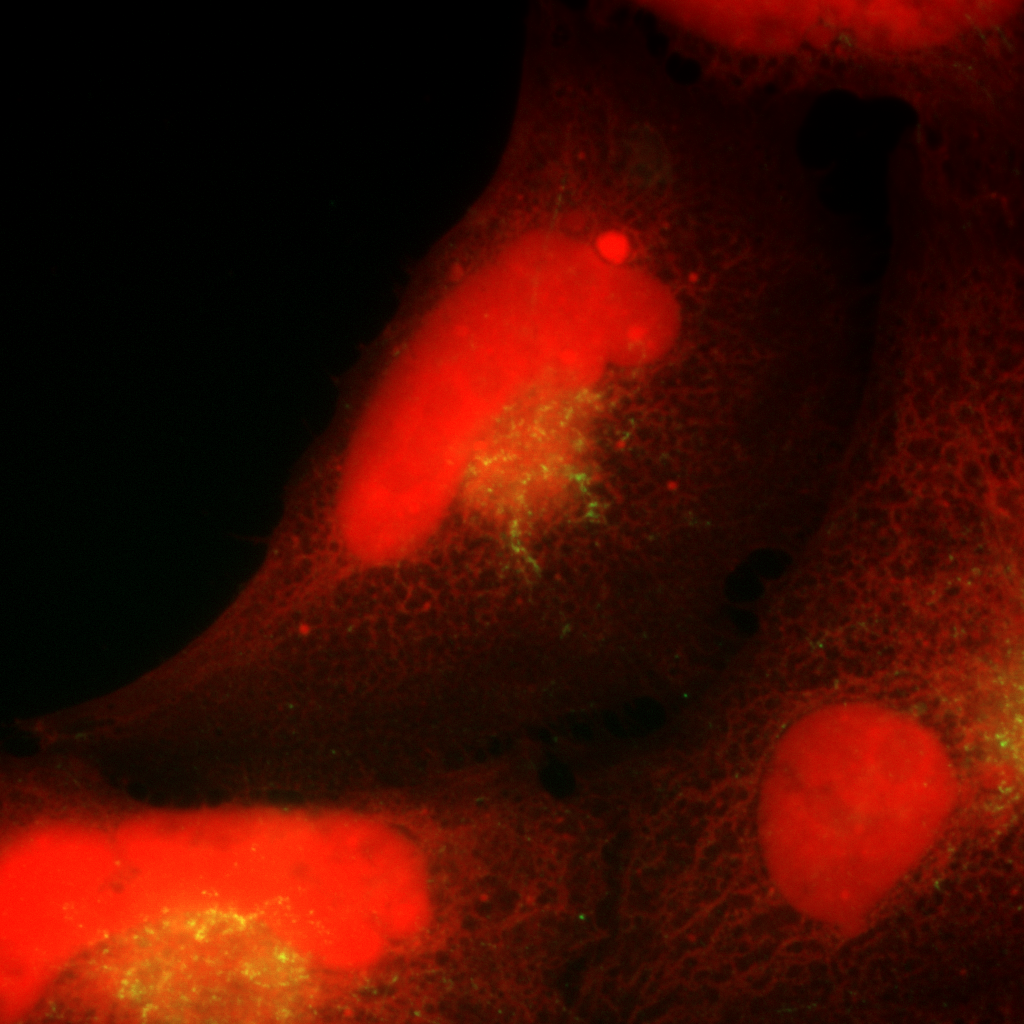

Supplement: S2 Dataset — Whole cell stain in red channel, endogenous SNX1 labelled in green channel. (ZIP) [file pone.0168294.s005.zip › Example Spastin depletion Images/Spastin knockdown 13.tif]

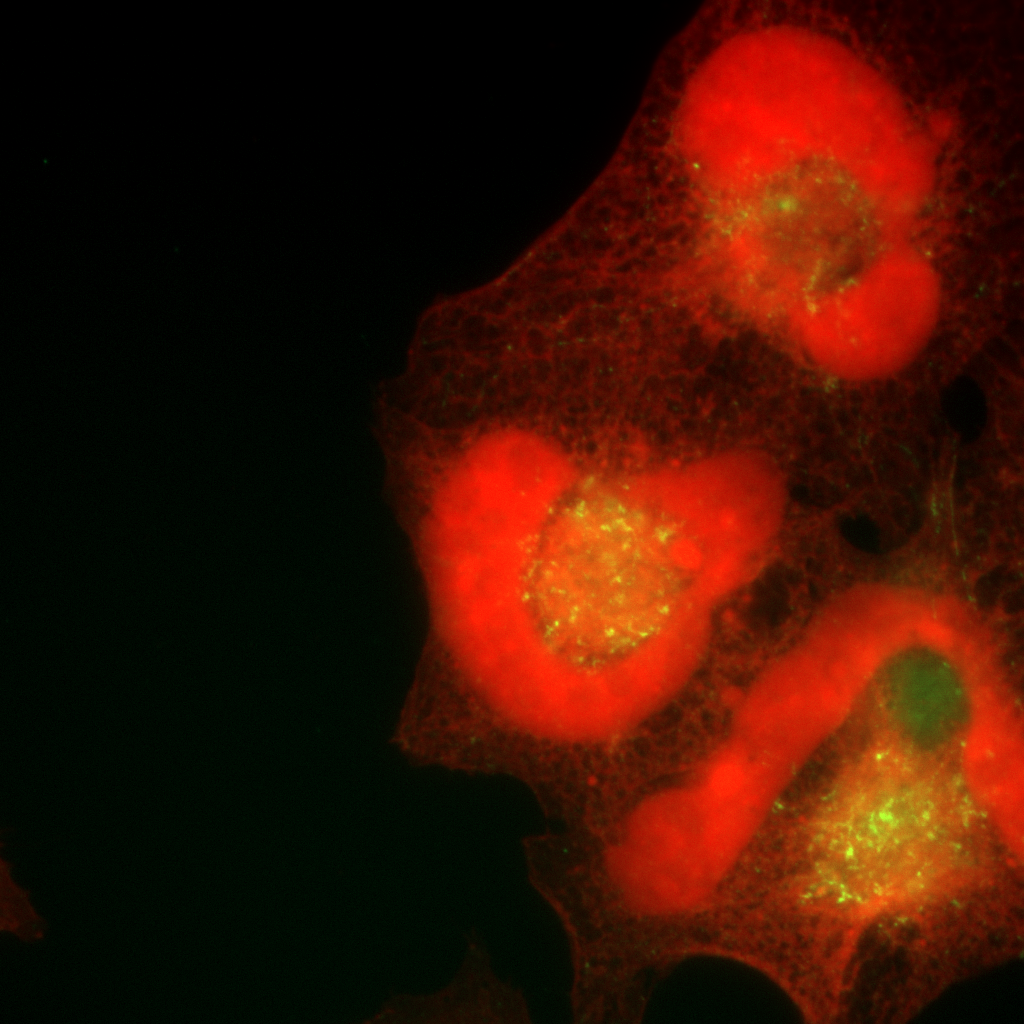

Supplement: S2 Dataset — Whole cell stain in red channel, endogenous SNX1 labelled in green channel. (ZIP) [file pone.0168294.s005.zip › Example Spastin depletion Images/Spastin knockdown 14.tif]

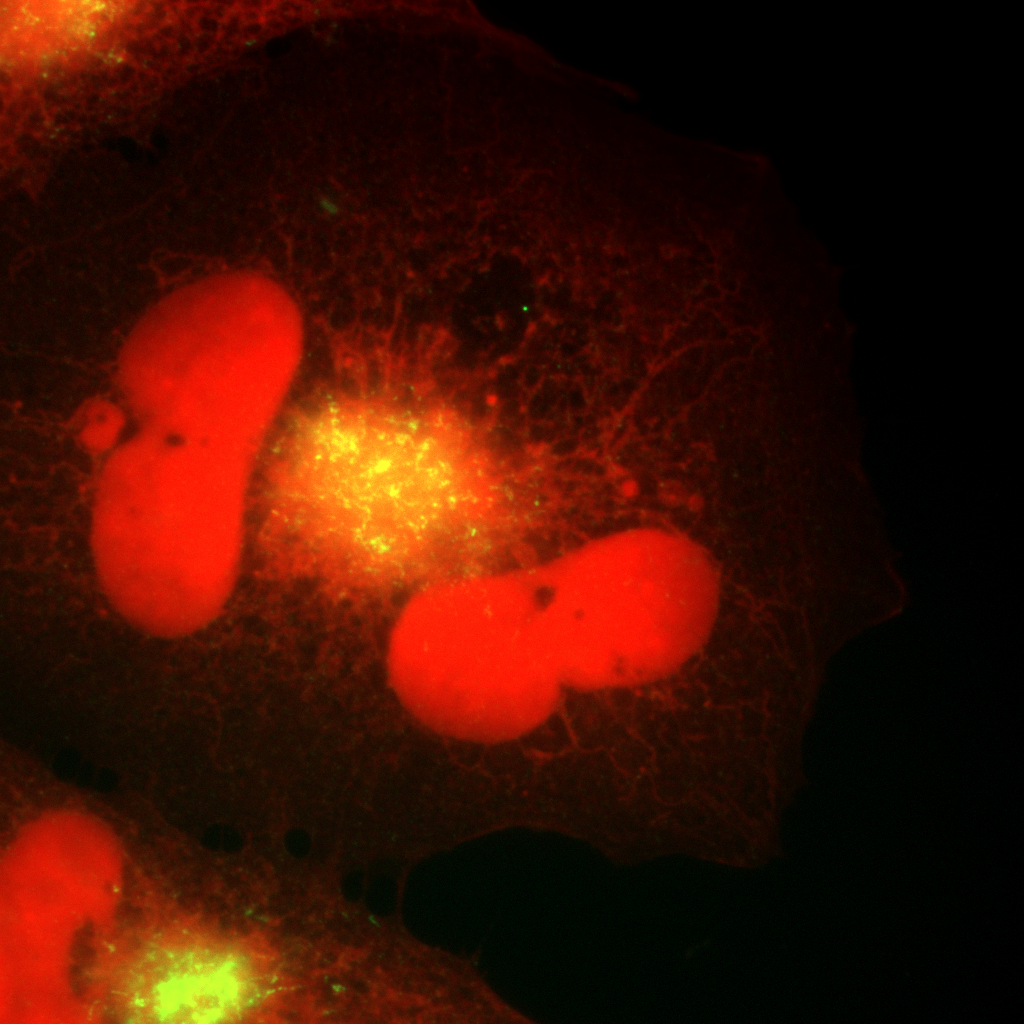

Supplement: S2 Dataset — Whole cell stain in red channel, endogenous SNX1 labelled in green channel. (ZIP) [file pone.0168294.s005.zip › Example Spastin depletion Images/Spastin knockdown 15.tif]

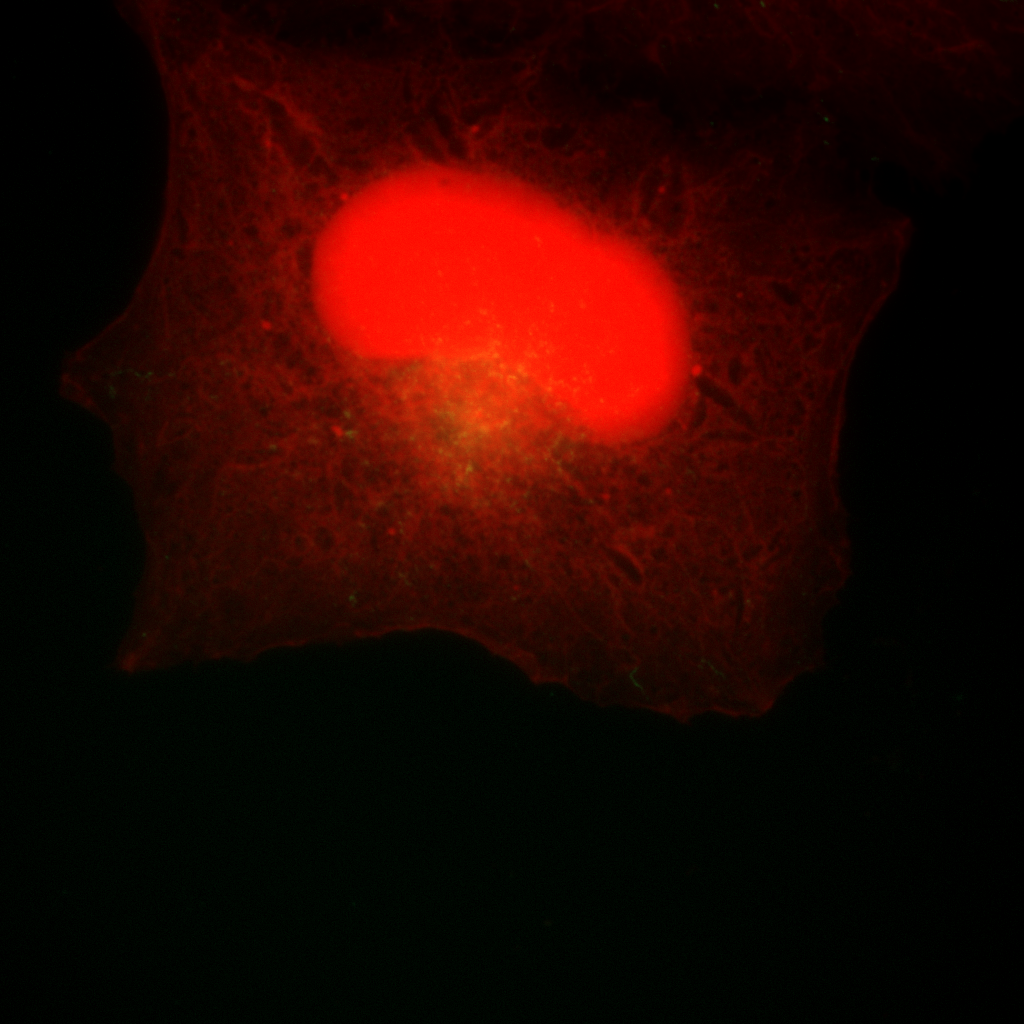

Supplement: S2 Dataset — Whole cell stain in red channel, endogenous SNX1 labelled in green channel. (ZIP) [file pone.0168294.s005.zip › Example Spastin depletion Images/Spastin knockdown 16.tif]

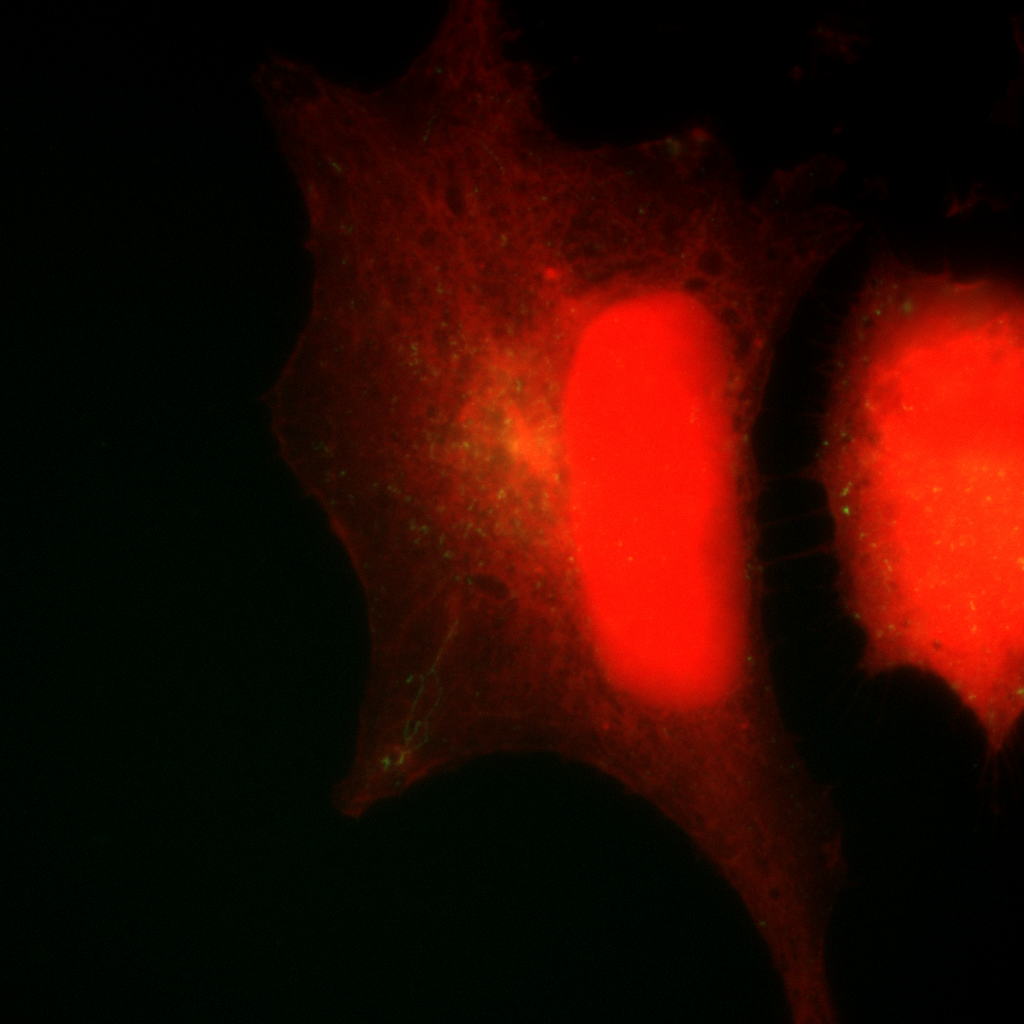

Supplement: S2 Dataset — Whole cell stain in red channel, endogenous SNX1 labelled in green channel. (ZIP) [file pone.0168294.s005.zip › Example Spastin depletion Images/Spastin knockdown 17.tif]

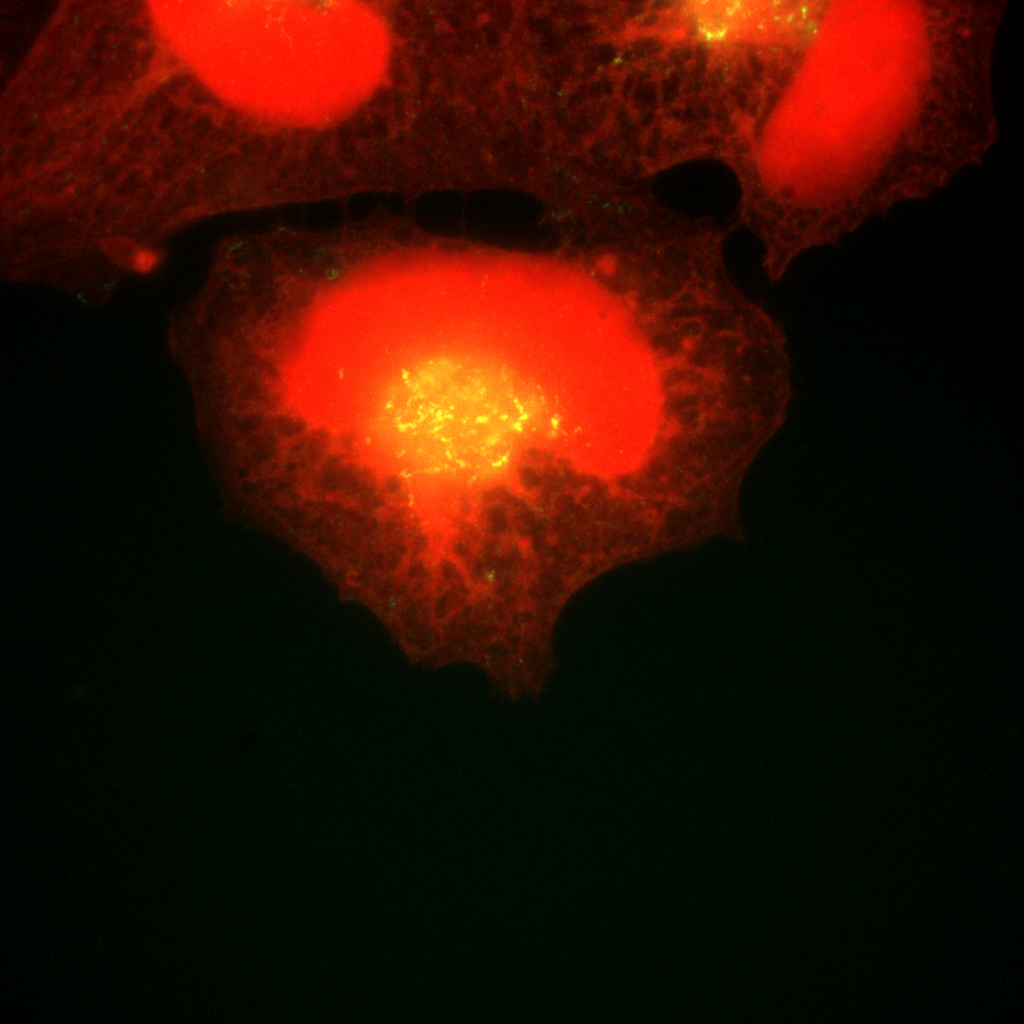

Supplement: S2 Dataset — Whole cell stain in red channel, endogenous SNX1 labelled in green channel. (ZIP) [file pone.0168294.s005.zip › Example Spastin depletion Images/Spastin knockdown 18.tif]

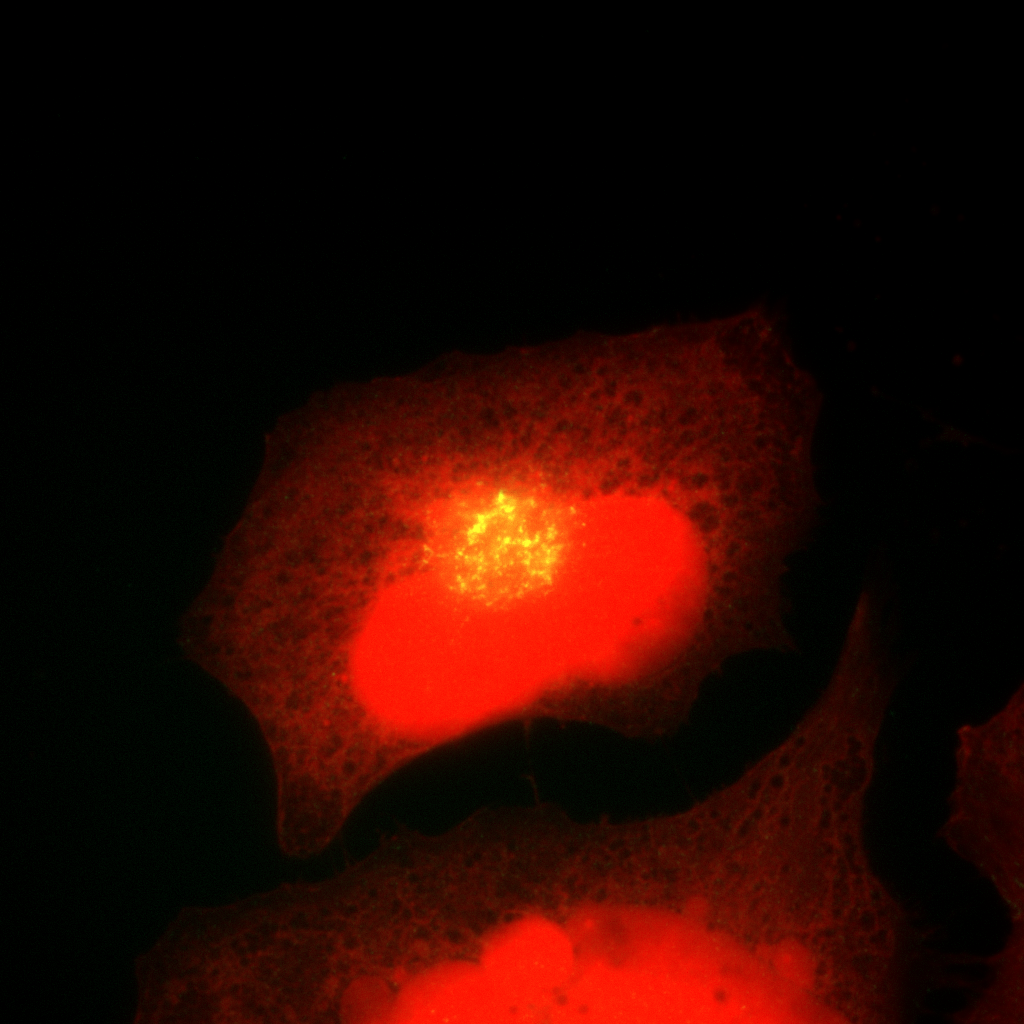

Supplement: S2 Dataset — Whole cell stain in red channel, endogenous SNX1 labelled in green channel. (ZIP) [file pone.0168294.s005.zip › Example Spastin depletion Images/Spastin knockdown 19.tif]

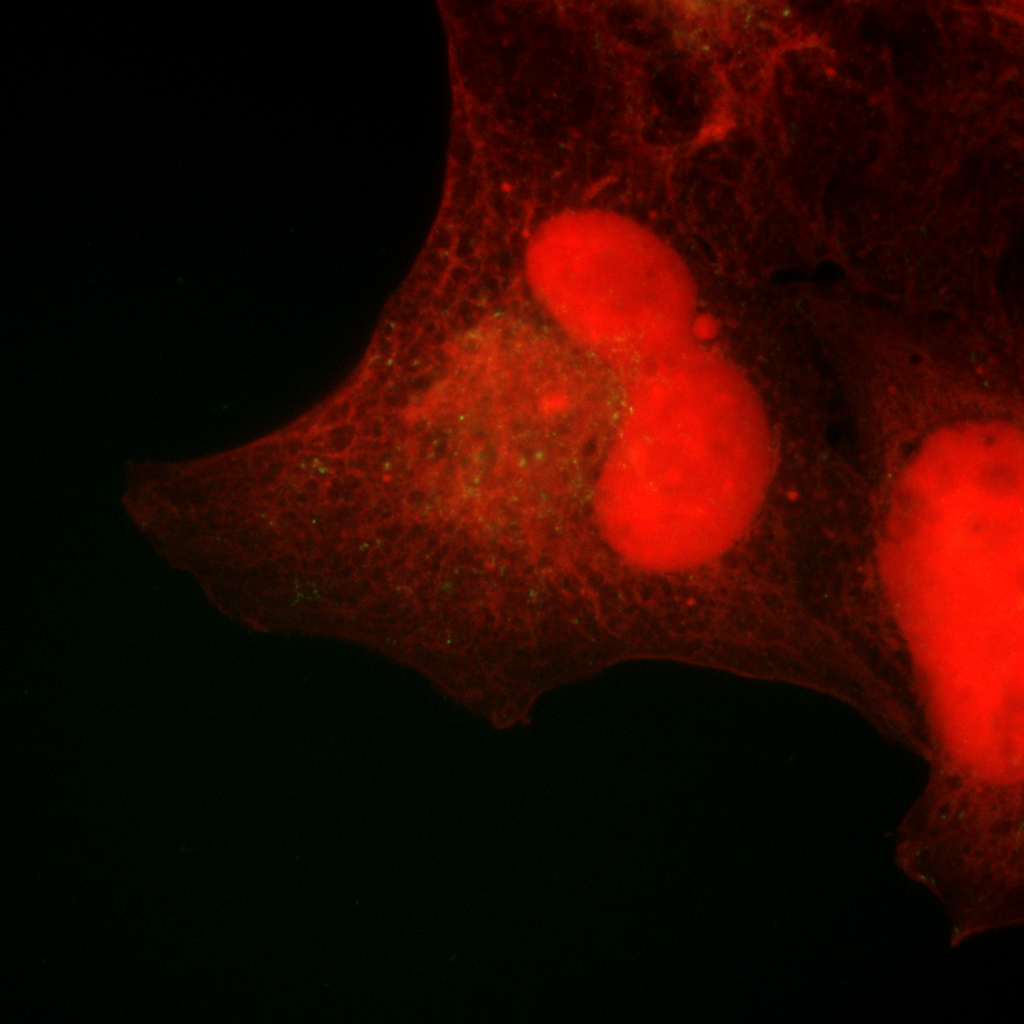

Supplement: S2 Dataset — Whole cell stain in red channel, endogenous SNX1 labelled in green channel. (ZIP) [file pone.0168294.s005.zip › Example Spastin depletion Images/Spastin knockdown 2.tif]

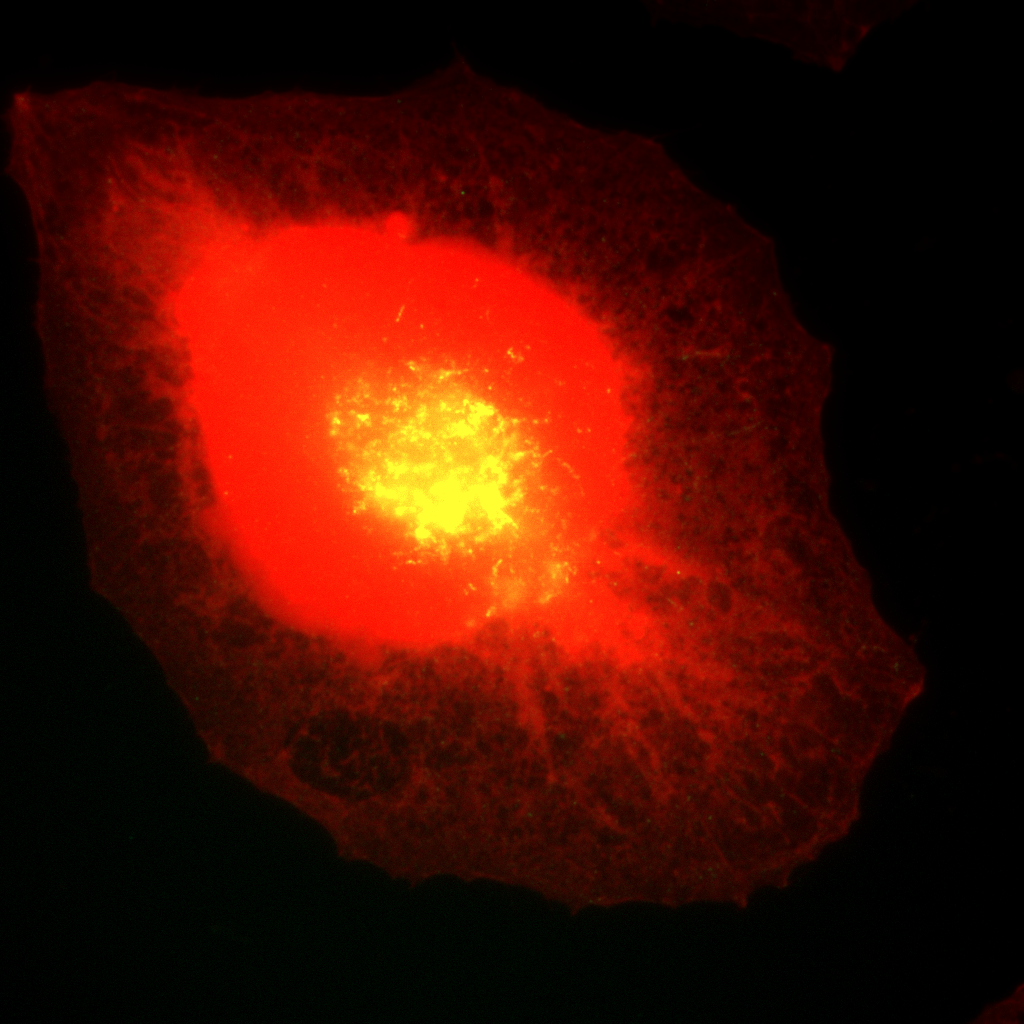

Supplement: S2 Dataset — Whole cell stain in red channel, endogenous SNX1 labelled in green channel. (ZIP) [file pone.0168294.s005.zip › Example Spastin depletion Images/Spastin knockdown 20.tif]

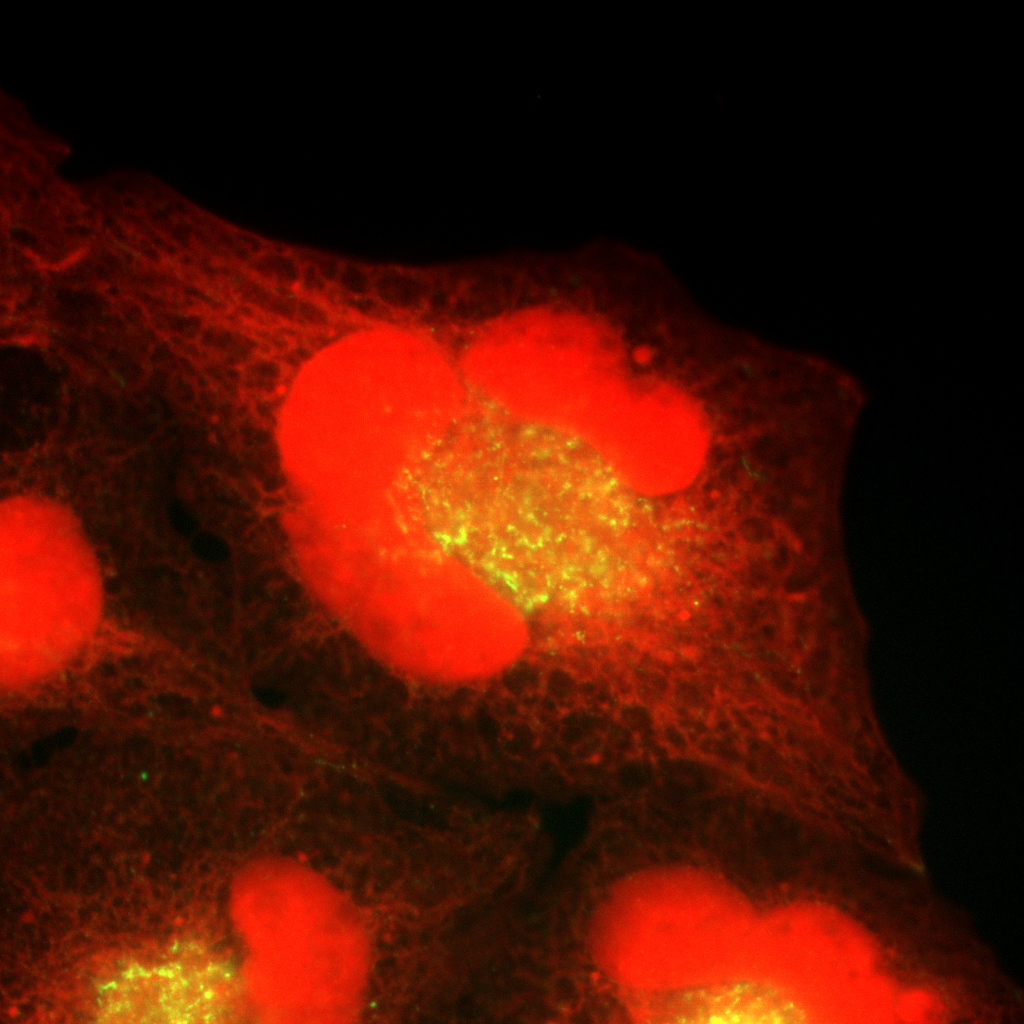

Supplement: S2 Dataset — Whole cell stain in red channel, endogenous SNX1 labelled in green channel. (ZIP) [file pone.0168294.s005.zip › Example Spastin depletion Images/Spastin knockdown 21.tif]

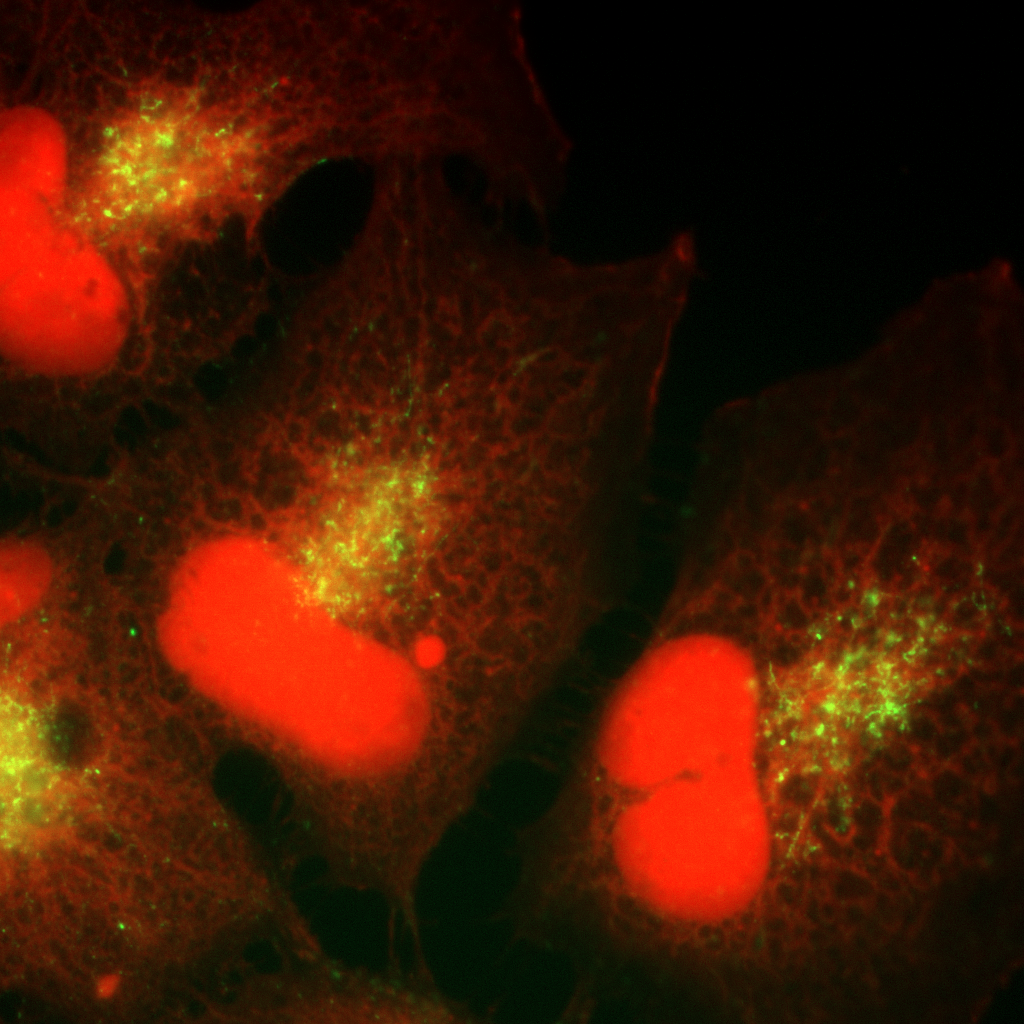

Supplement: S2 Dataset — Whole cell stain in red channel, endogenous SNX1 labelled in green channel. (ZIP) [file pone.0168294.s005.zip › Example Spastin depletion Images/Spastin knockdown 22.tif]

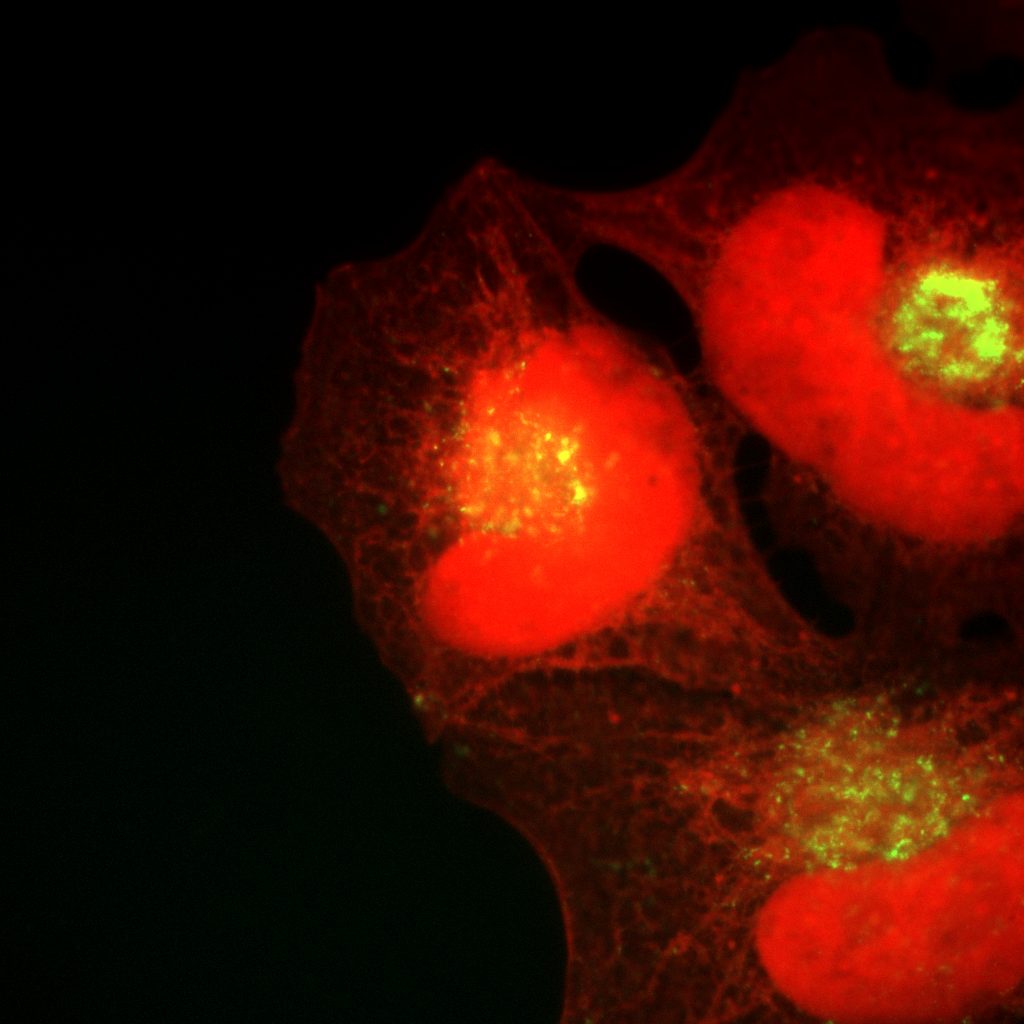

Supplement: S2 Dataset — Whole cell stain in red channel, endogenous SNX1 labelled in green channel. (ZIP) [file pone.0168294.s005.zip › Example Spastin depletion Images/Spastin knockdown 23.tif]

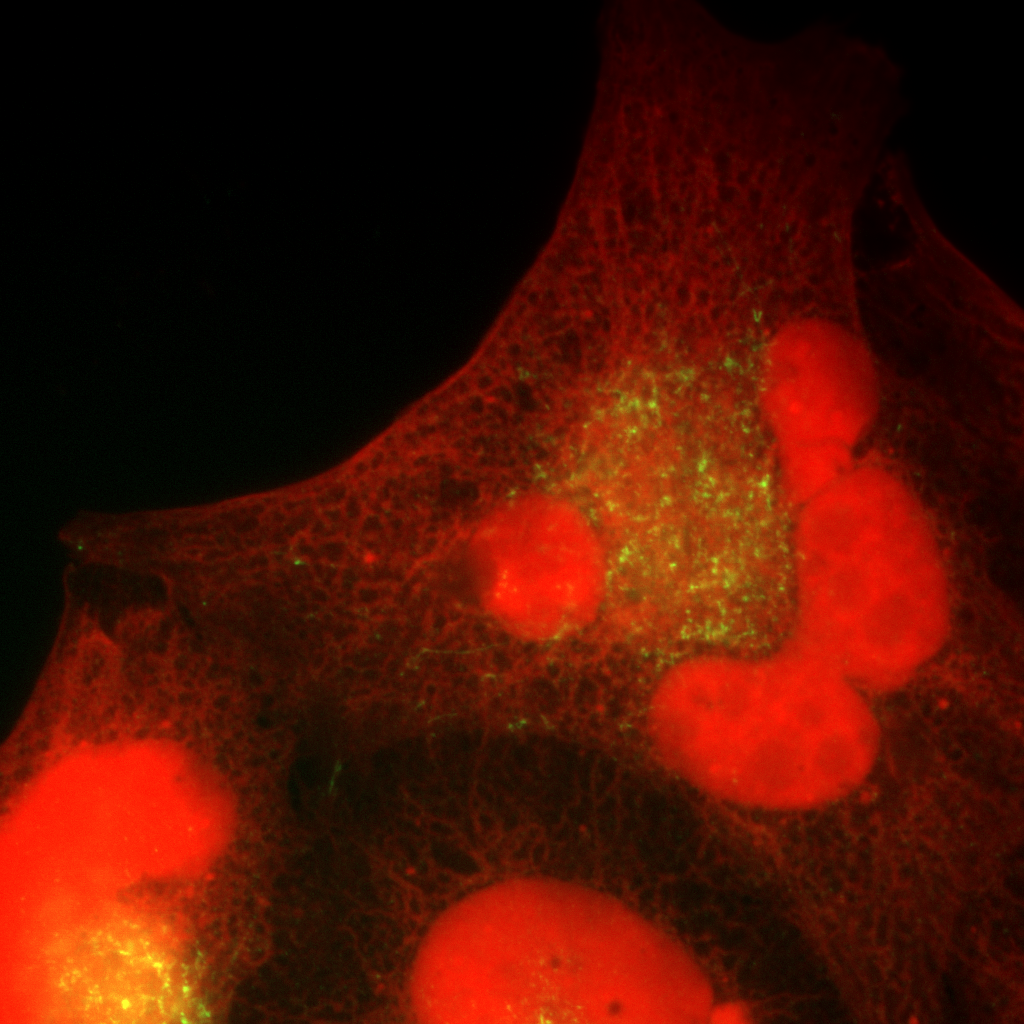

Supplement: S2 Dataset — Whole cell stain in red channel, endogenous SNX1 labelled in green channel. (ZIP) [file pone.0168294.s005.zip › Example Spastin depletion Images/Spastin knockdown 24.tif]

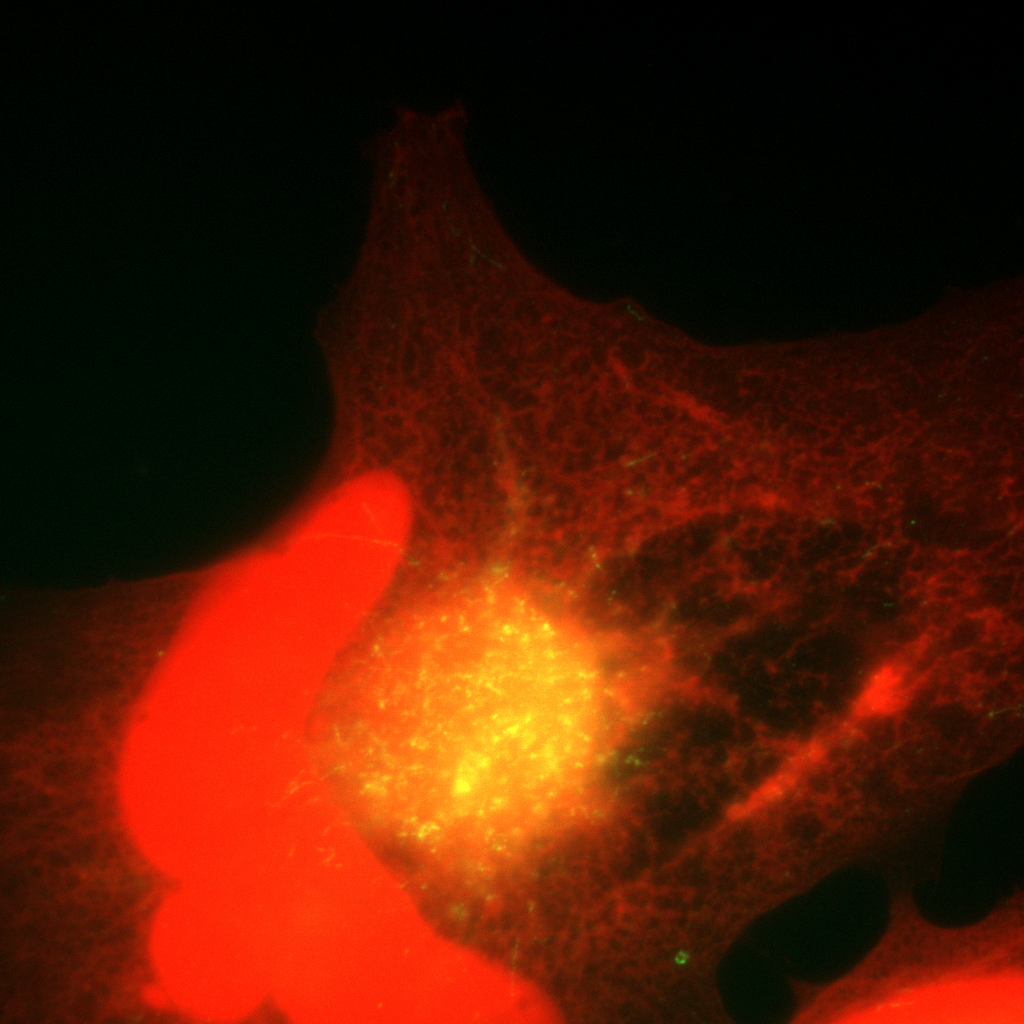

Supplement: S2 Dataset — Whole cell stain in red channel, endogenous SNX1 labelled in green channel. (ZIP) [file pone.0168294.s005.zip › Example Spastin depletion Images/Spastin knockdown 25.tif]

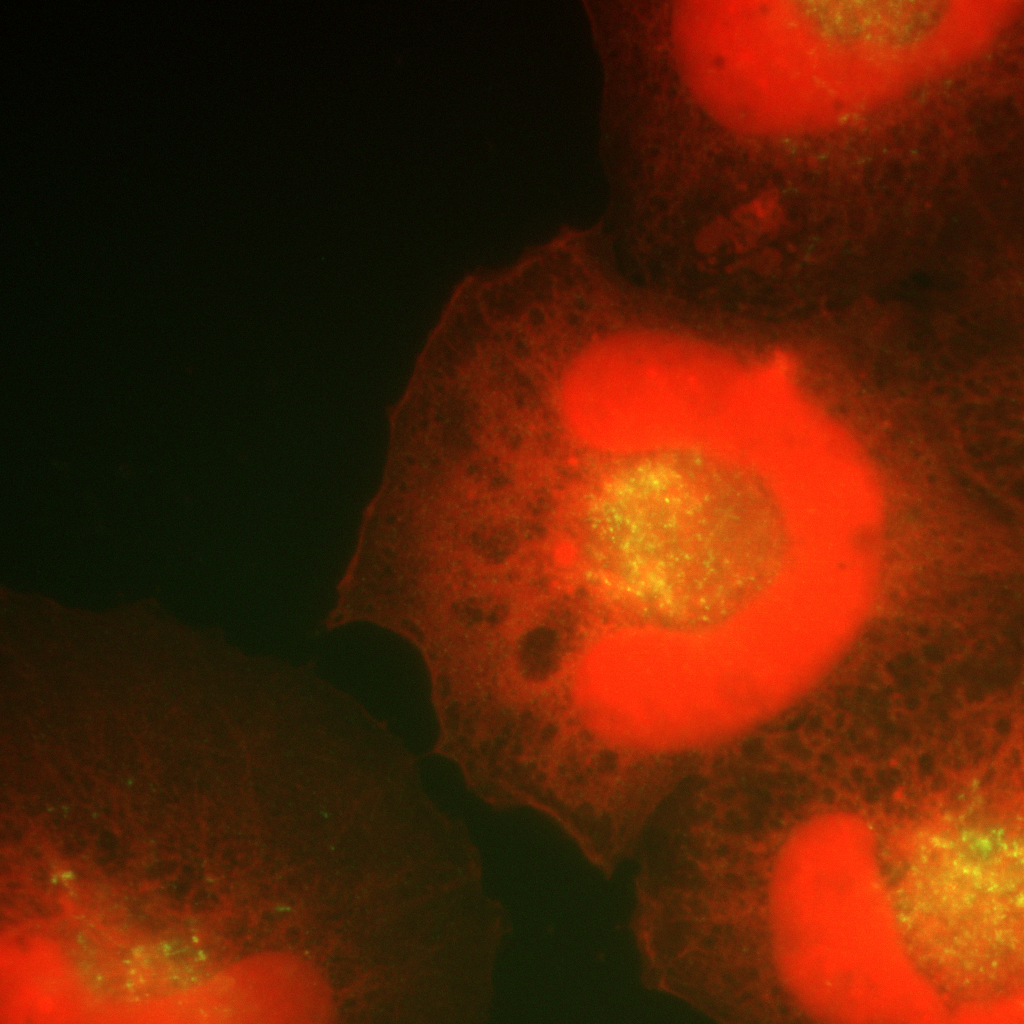

Supplement: S2 Dataset — Whole cell stain in red channel, endogenous SNX1 labelled in green channel. (ZIP) [file pone.0168294.s005.zip › Example Spastin depletion Images/Spastin knockdown 26.tif]

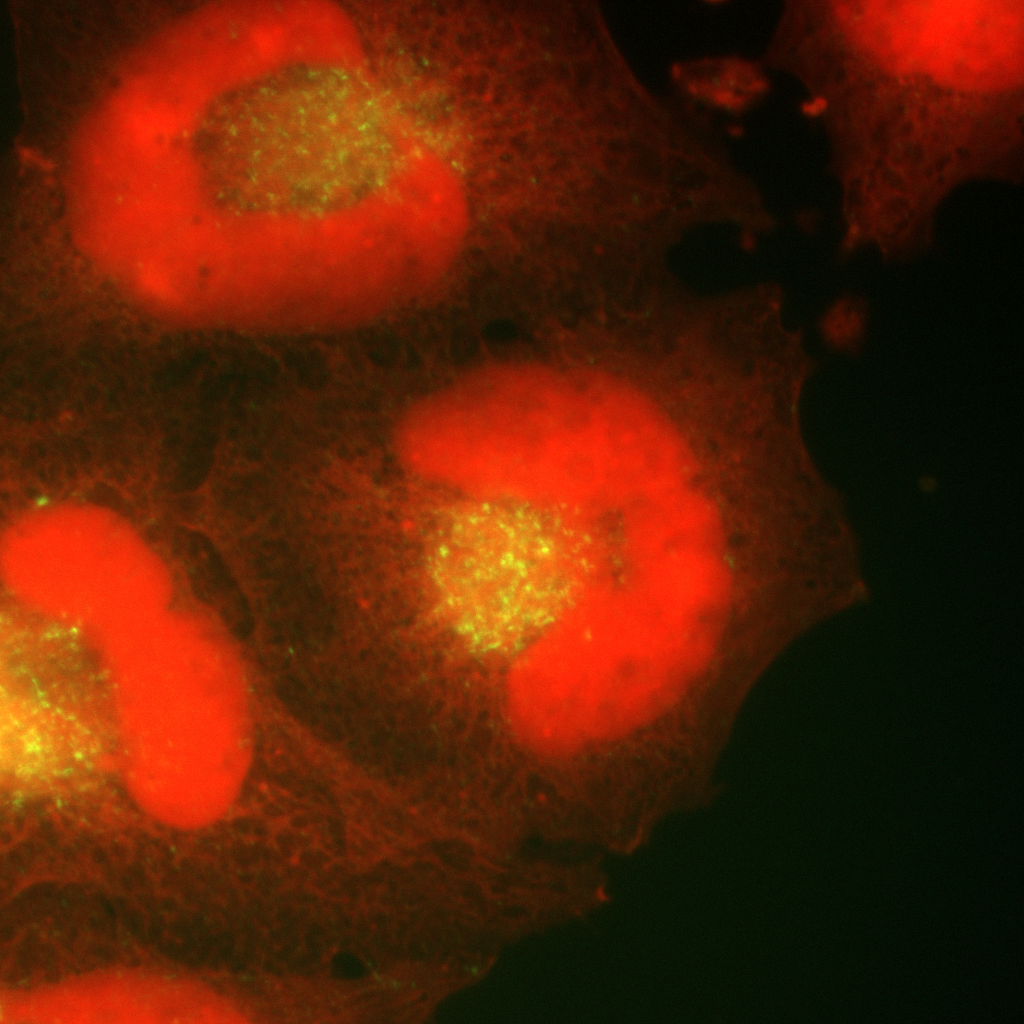

Supplement: S2 Dataset — Whole cell stain in red channel, endogenous SNX1 labelled in green channel. (ZIP) [file pone.0168294.s005.zip › Example Spastin depletion Images/Spastin knockdown 27.tif]

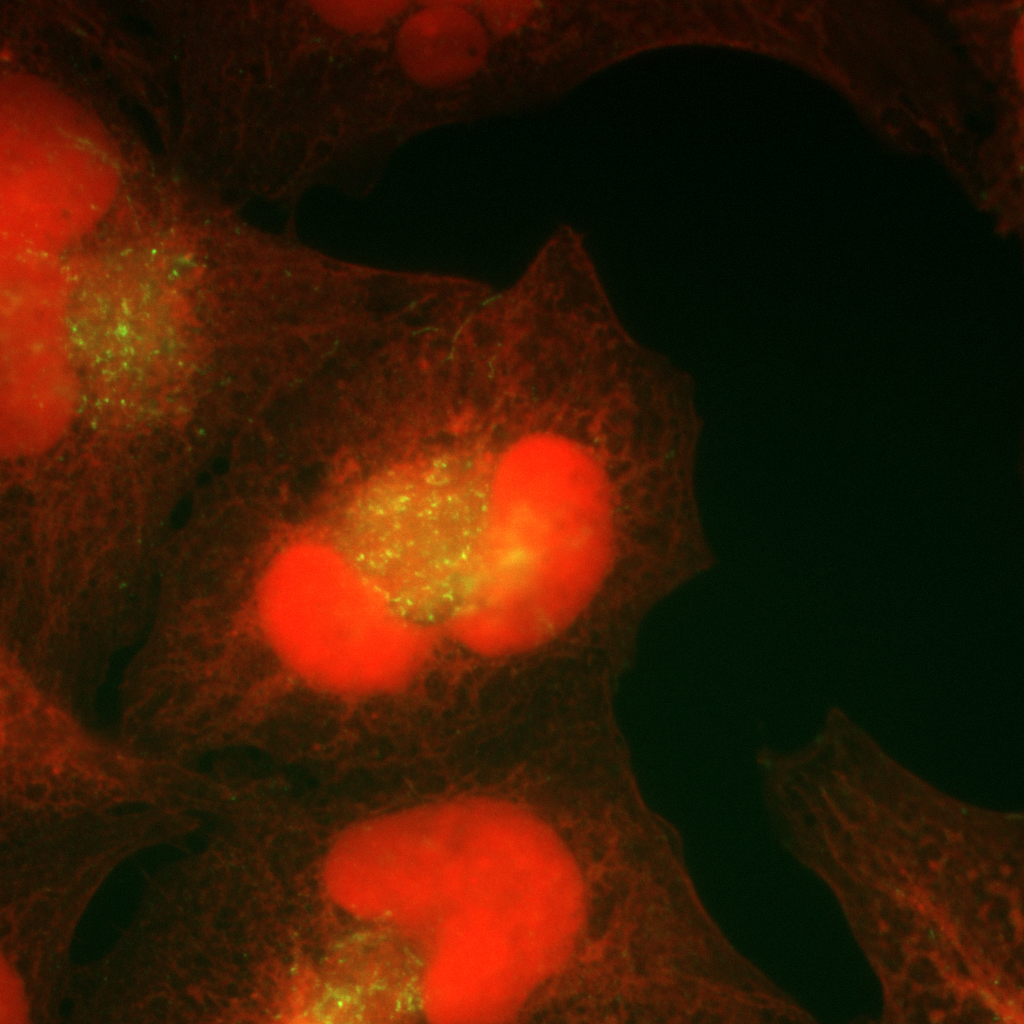

Supplement: S2 Dataset — Whole cell stain in red channel, endogenous SNX1 labelled in green channel. (ZIP) [file pone.0168294.s005.zip › Example Spastin depletion Images/Spastin knockdown 28.tif]

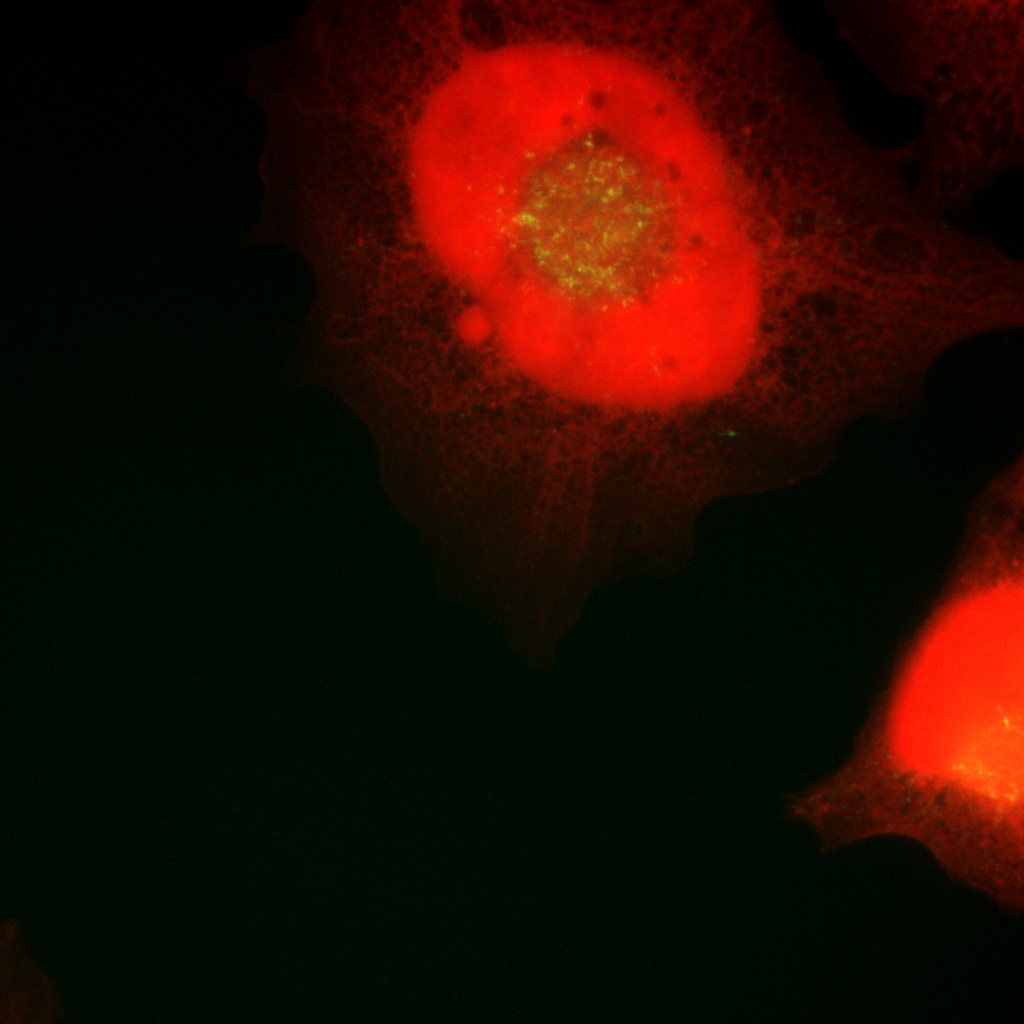

Supplement: S2 Dataset — Whole cell stain in red channel, endogenous SNX1 labelled in green channel. (ZIP) [file pone.0168294.s005.zip › Example Spastin depletion Images/Spastin knockdown 29.tif]

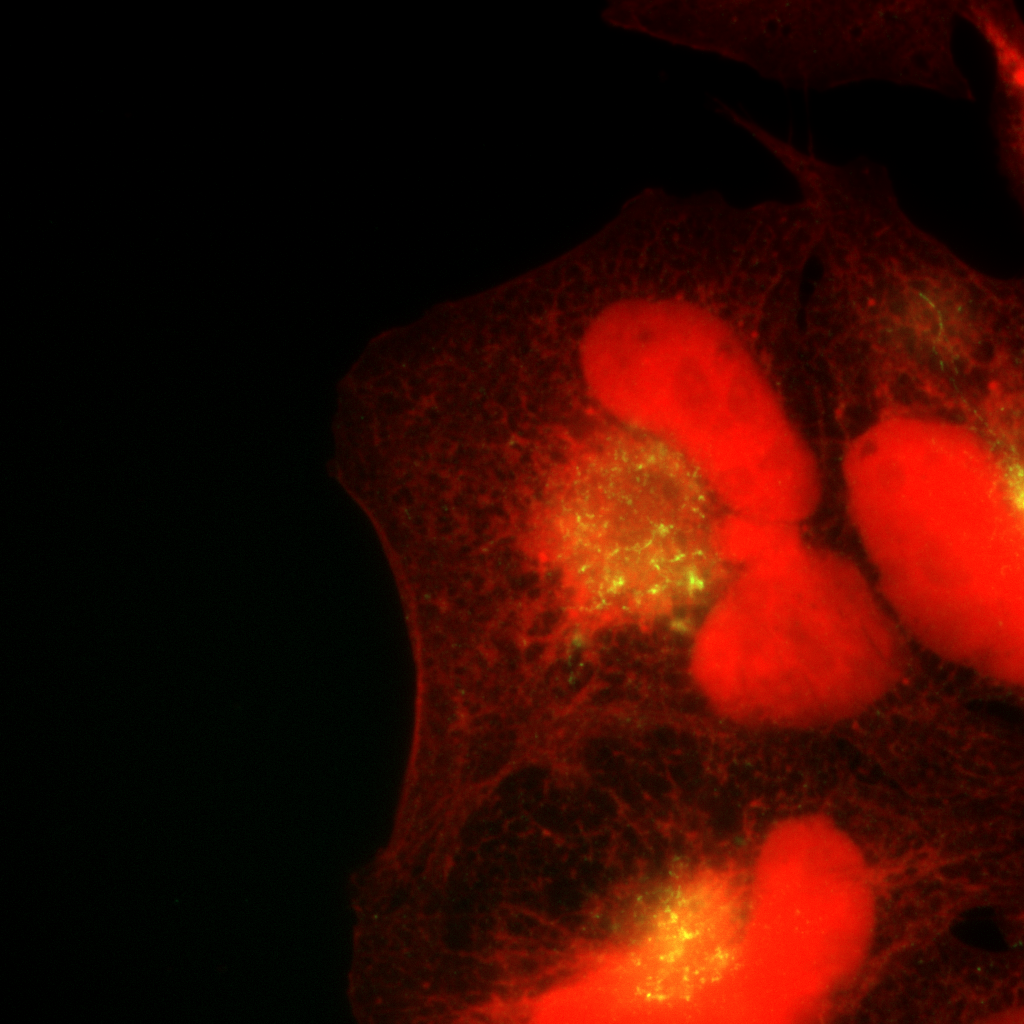

Supplement: S2 Dataset — Whole cell stain in red channel, endogenous SNX1 labelled in green channel. (ZIP) [file pone.0168294.s005.zip › Example Spastin depletion Images/Spastin knockdown 3.tif]

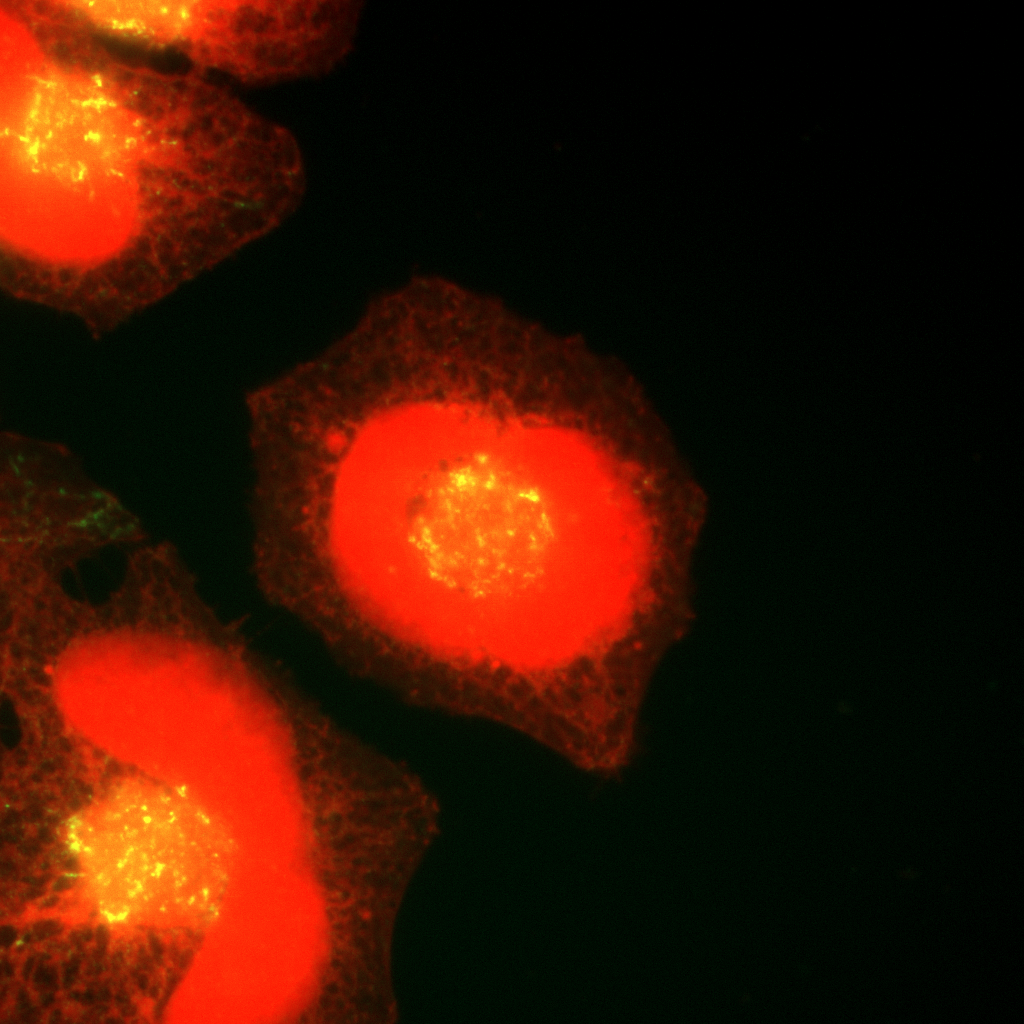

Supplement: S2 Dataset — Whole cell stain in red channel, endogenous SNX1 labelled in green channel. (ZIP) [file pone.0168294.s005.zip › Example Spastin depletion Images/Spastin knockdown 30.tif]

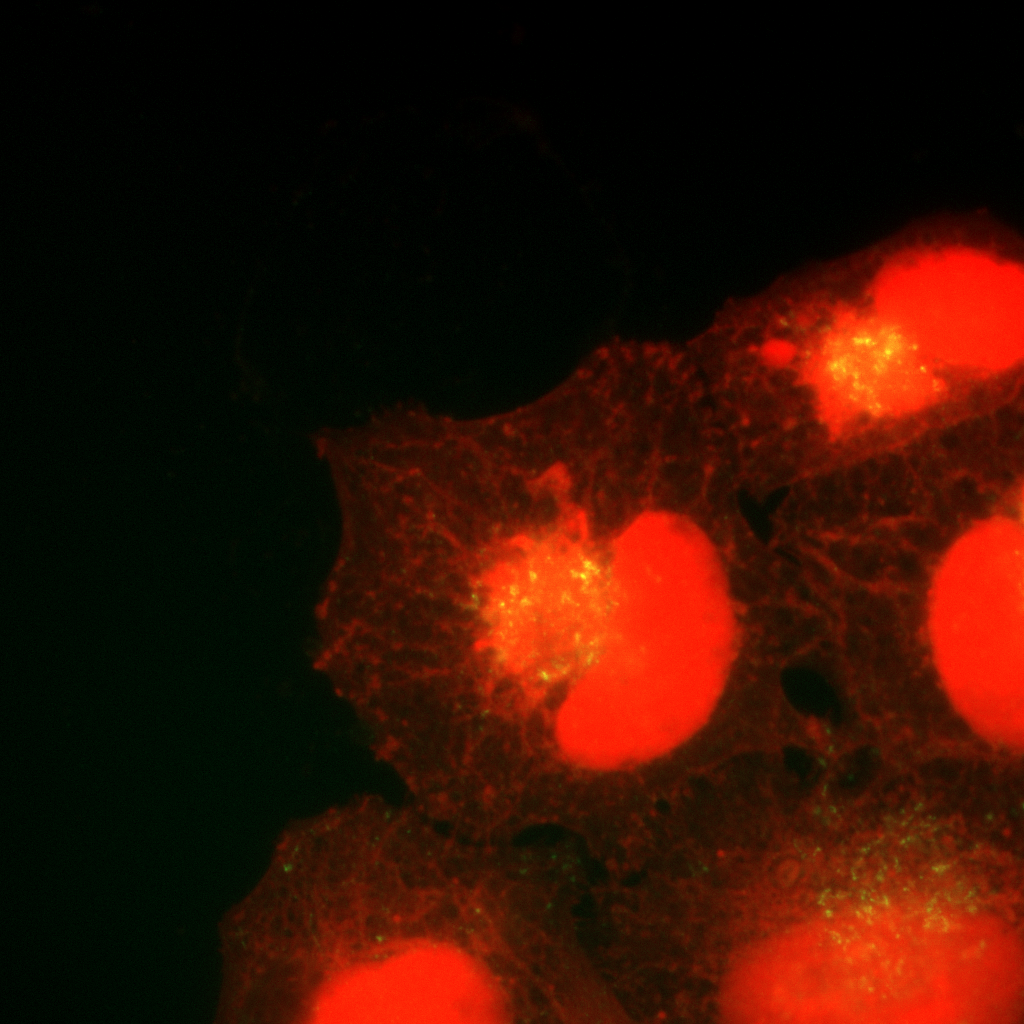

Supplement: S2 Dataset — Whole cell stain in red channel, endogenous SNX1 labelled in green channel. (ZIP) [file pone.0168294.s005.zip › Example Spastin depletion Images/Spastin knockdown 31.tif]

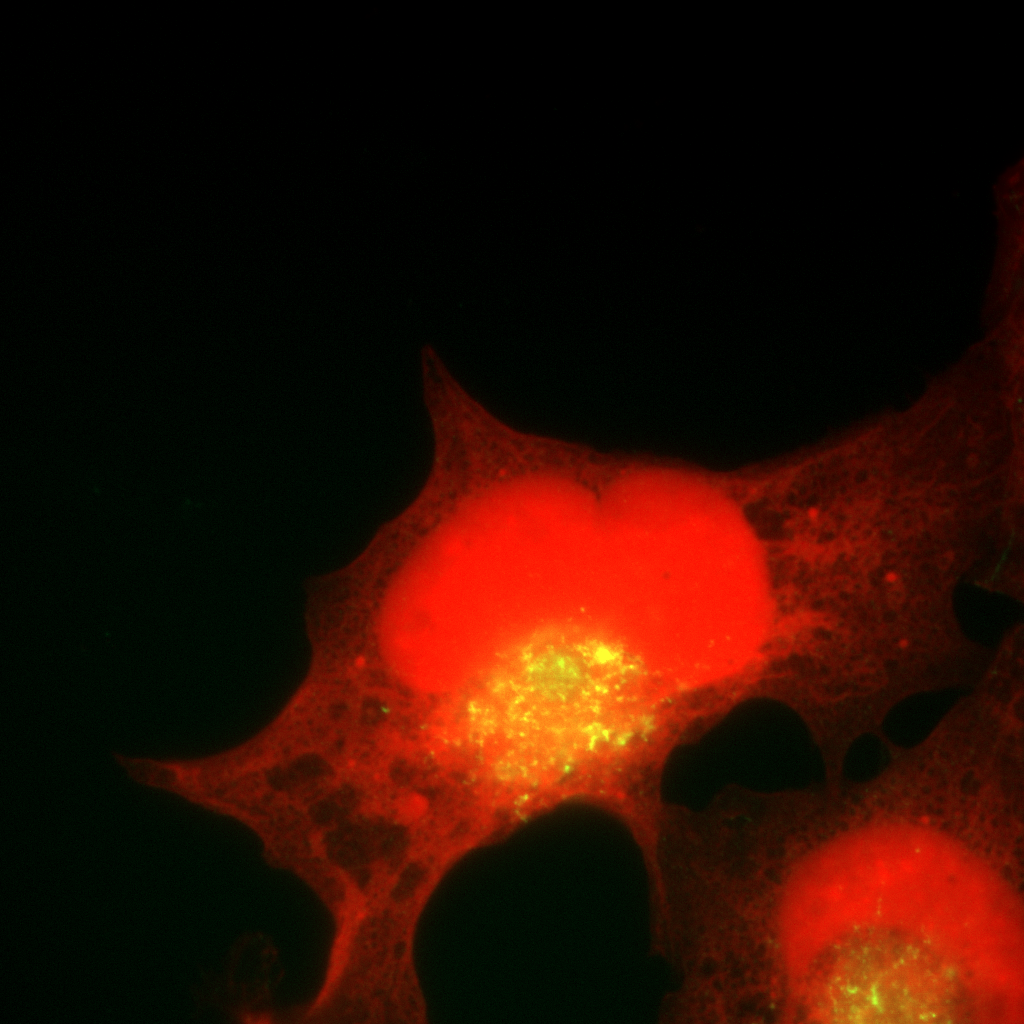

Supplement: S2 Dataset — Whole cell stain in red channel, endogenous SNX1 labelled in green channel. (ZIP) [file pone.0168294.s005.zip › Example Spastin depletion Images/Spastin knockdown 32.tif]

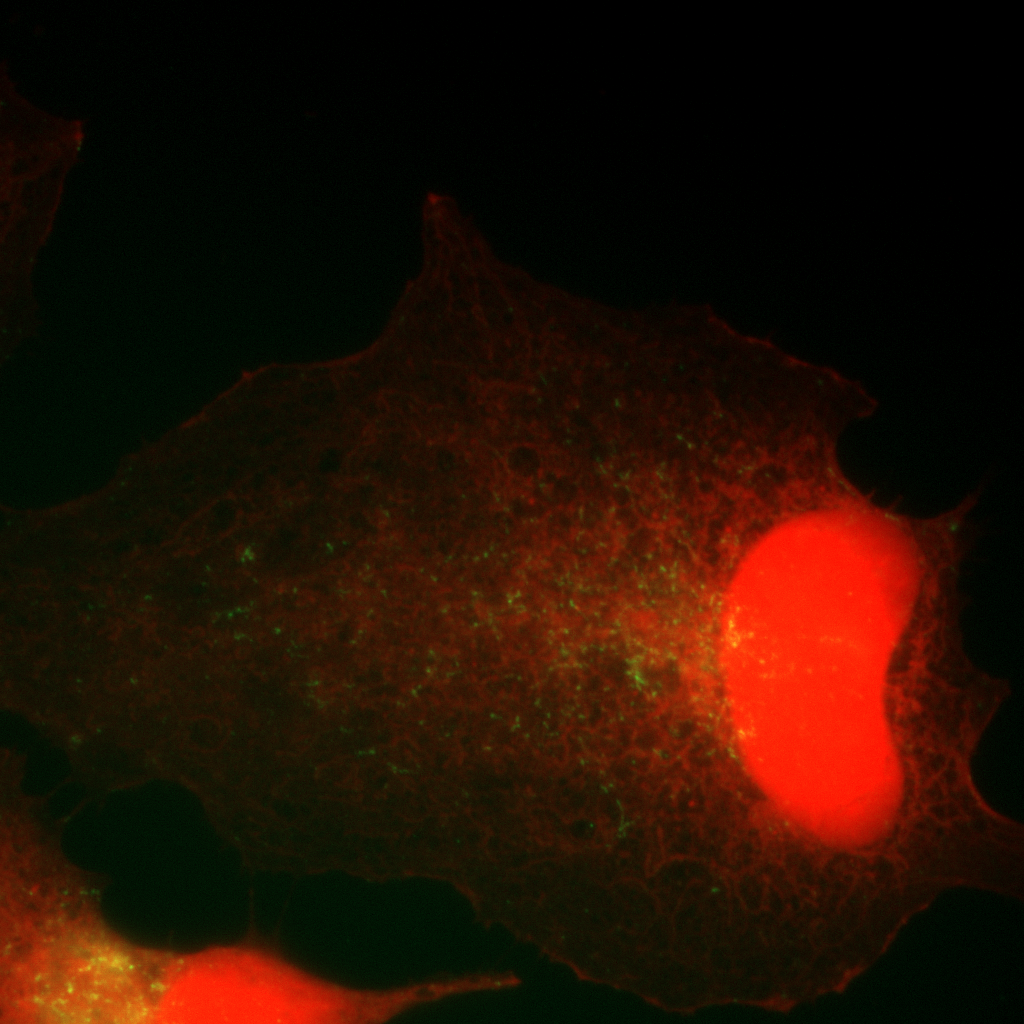

Supplement: S2 Dataset — Whole cell stain in red channel, endogenous SNX1 labelled in green channel. (ZIP) [file pone.0168294.s005.zip › Example Spastin depletion Images/Spastin knockdown 33.tif]

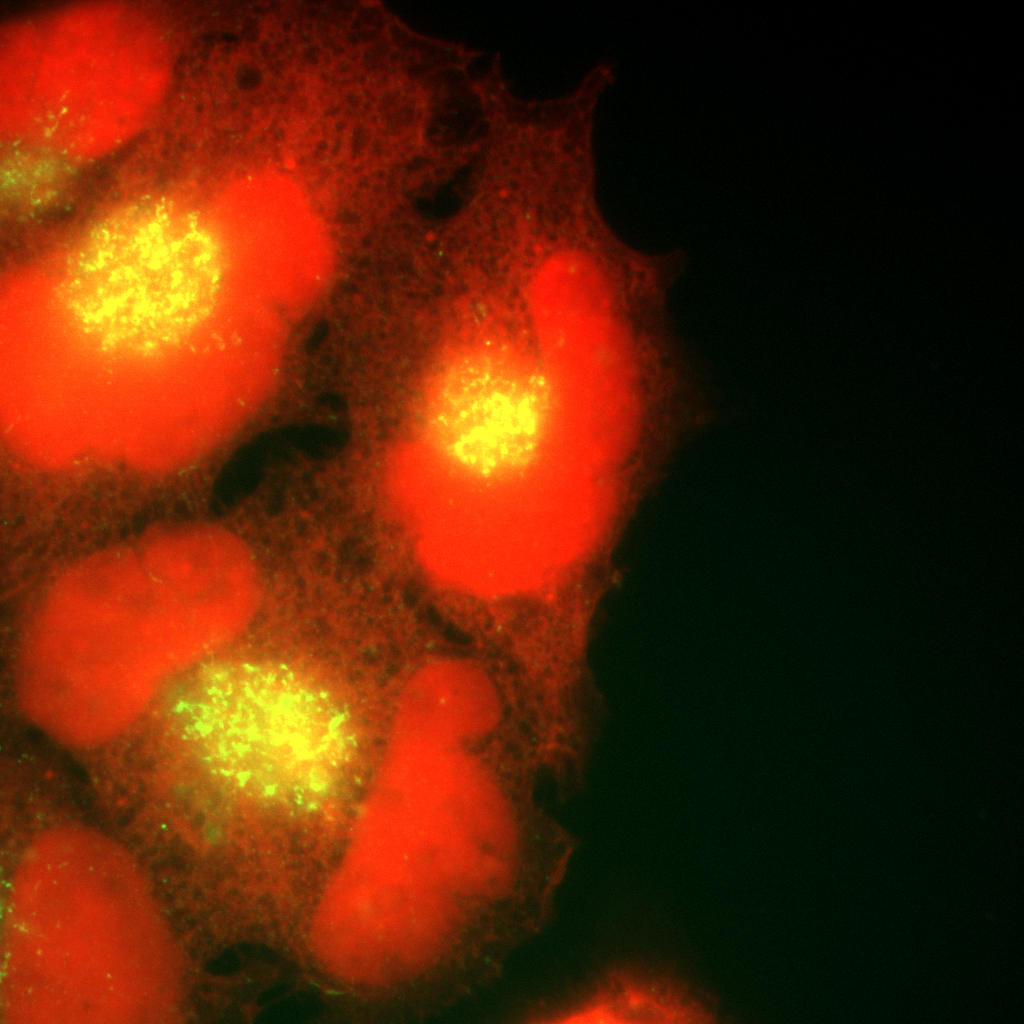

Supplement: S2 Dataset — Whole cell stain in red channel, endogenous SNX1 labelled in green channel. (ZIP) [file pone.0168294.s005.zip › Example Spastin depletion Images/Spastin knockdown 34.tif]

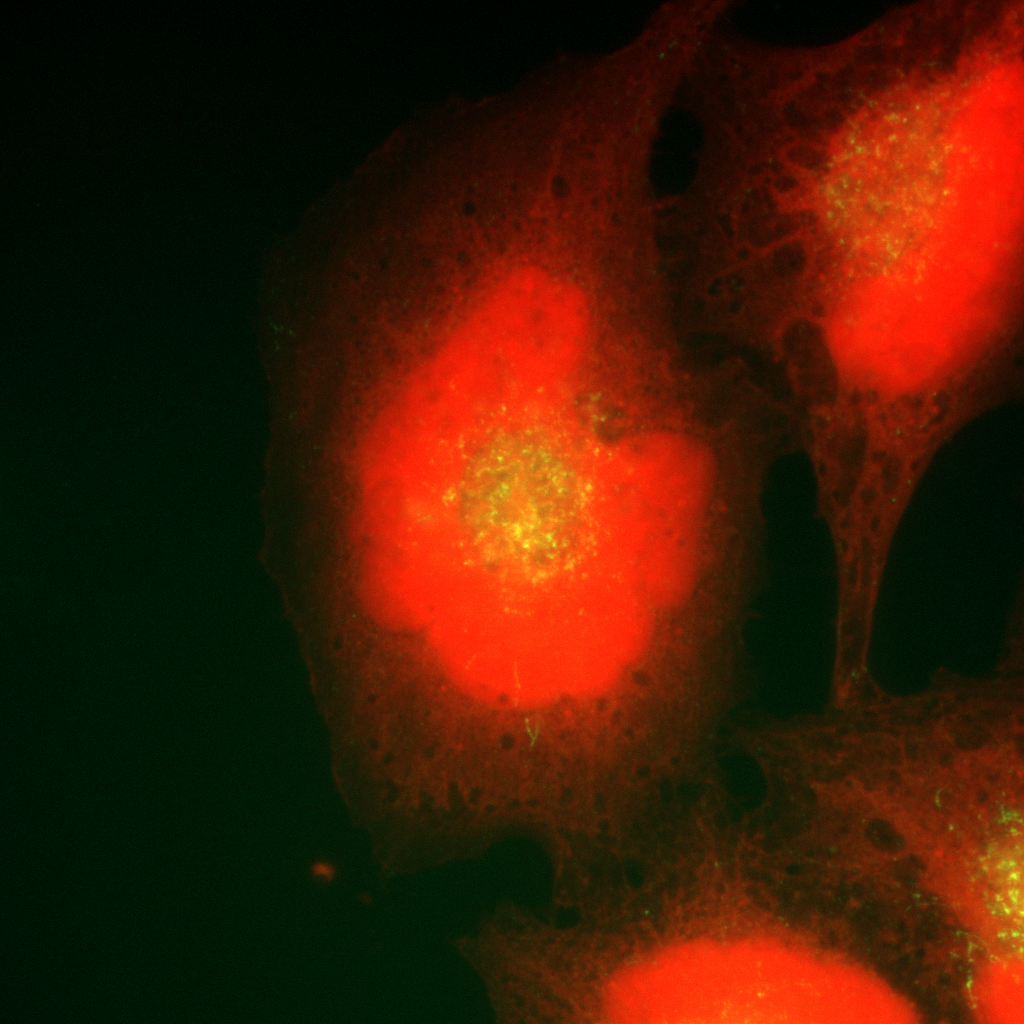

Supplement: S2 Dataset — Whole cell stain in red channel, endogenous SNX1 labelled in green channel. (ZIP) [file pone.0168294.s005.zip › Example Spastin depletion Images/Spastin knockdown 35.tif]

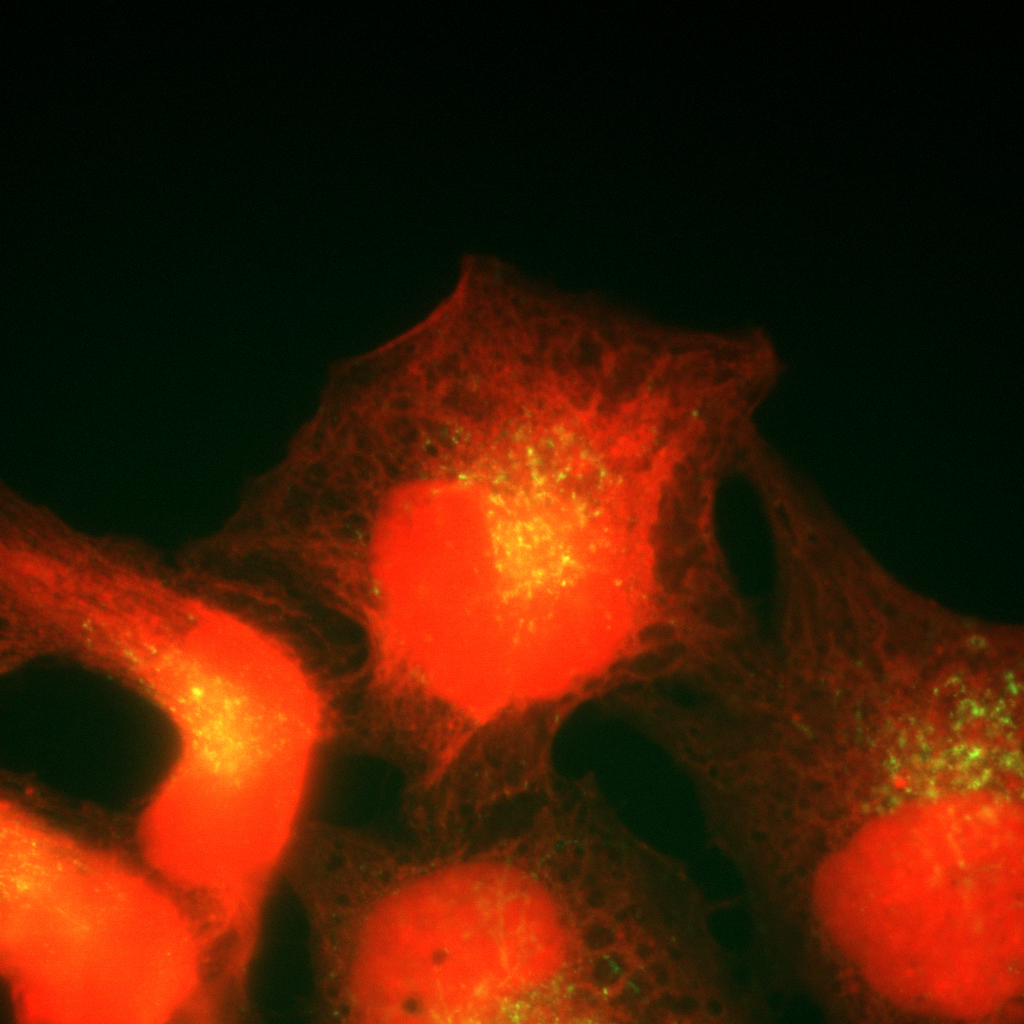

Supplement: S2 Dataset — Whole cell stain in red channel, endogenous SNX1 labelled in green channel. (ZIP) [file pone.0168294.s005.zip › Example Spastin depletion Images/Spastin knockdown 36.tif]

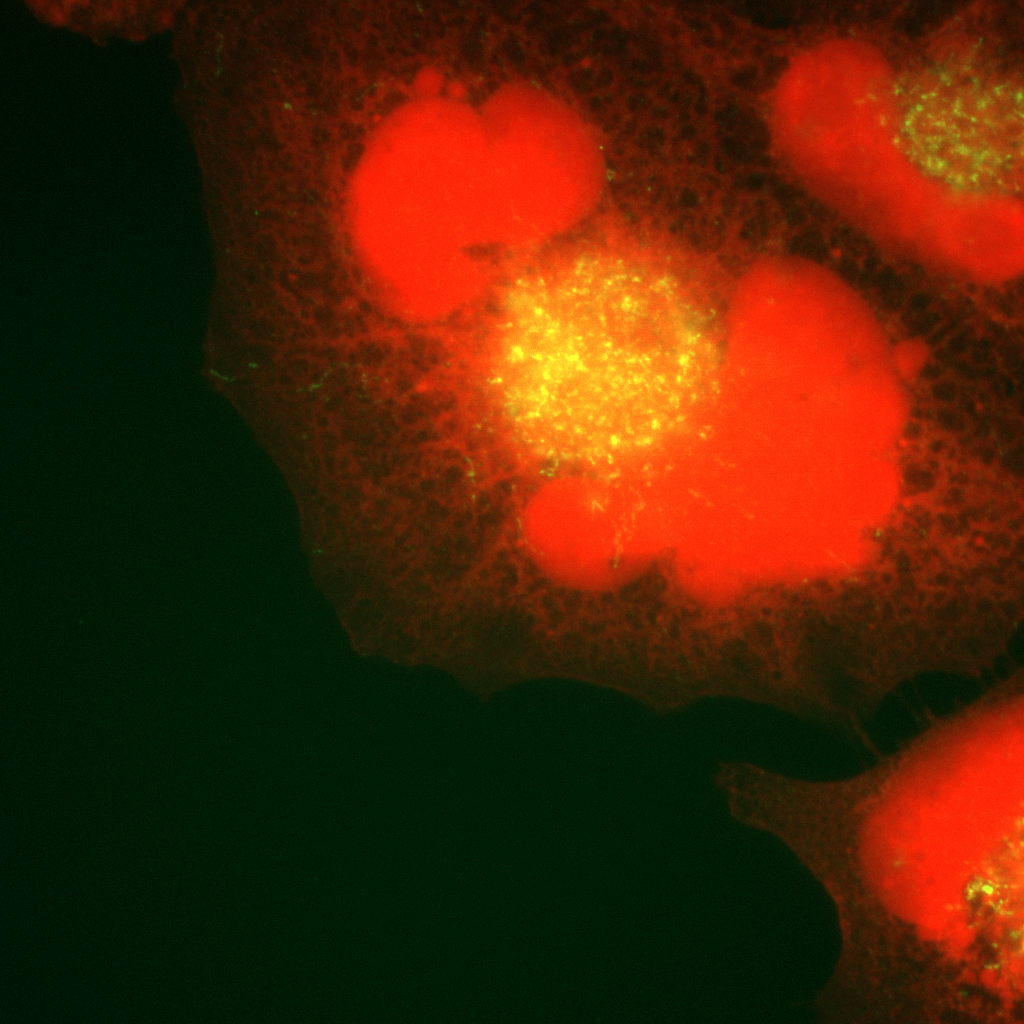

Supplement: S2 Dataset — Whole cell stain in red channel, endogenous SNX1 labelled in green channel. (ZIP) [file pone.0168294.s005.zip › Example Spastin depletion Images/Spastin knockdown 37.tif]

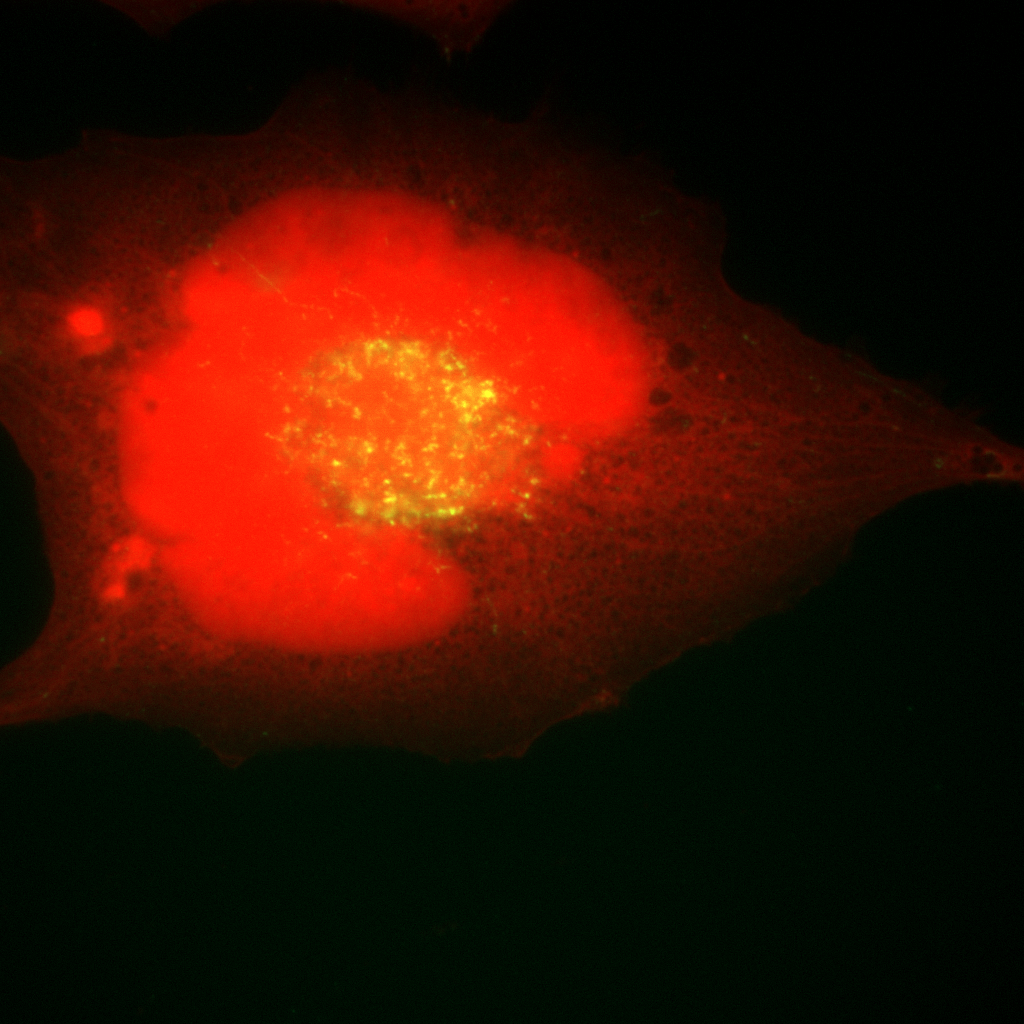

Supplement: S2 Dataset — Whole cell stain in red channel, endogenous SNX1 labelled in green channel. (ZIP) [file pone.0168294.s005.zip › Example Spastin depletion Images/Spastin knockdown 38.tif]

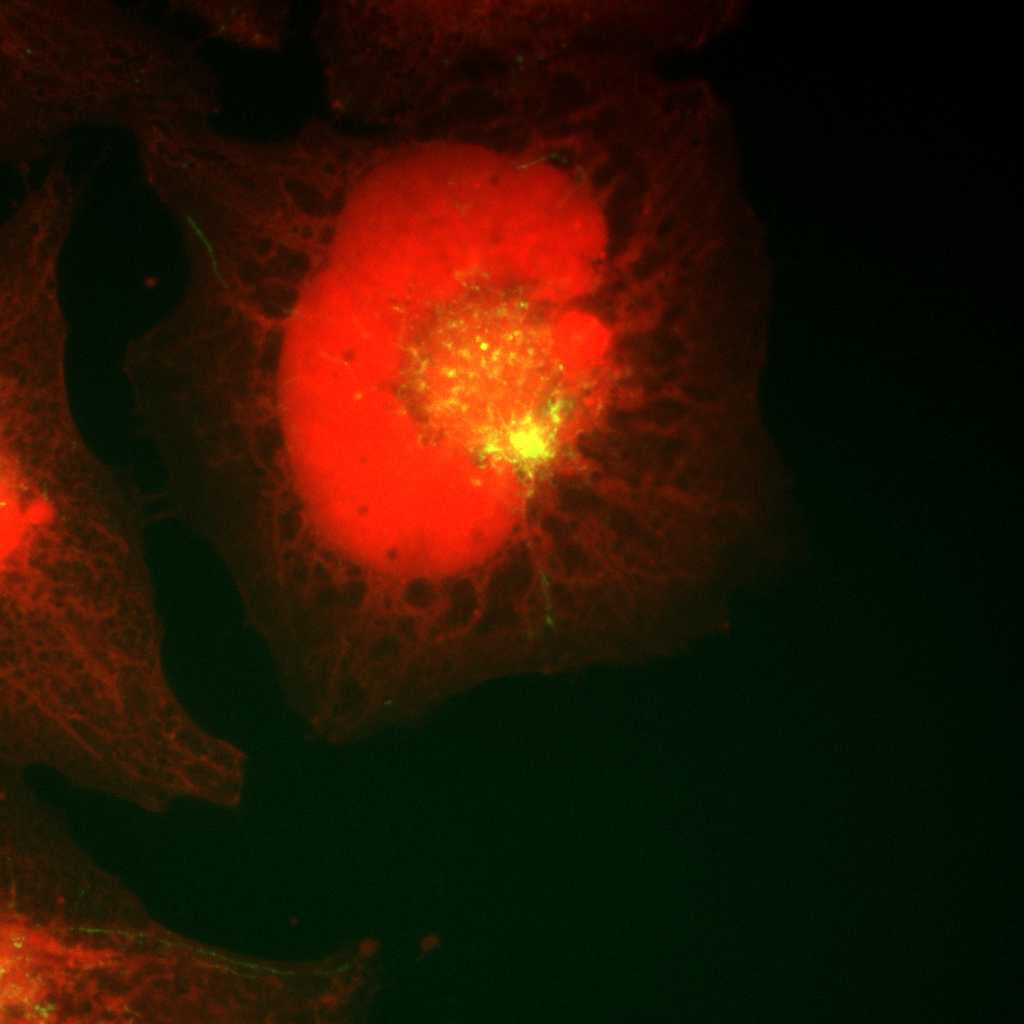

Supplement: S2 Dataset — Whole cell stain in red channel, endogenous SNX1 labelled in green channel. (ZIP) [file pone.0168294.s005.zip › Example Spastin depletion Images/Spastin knockdown 39.tif]

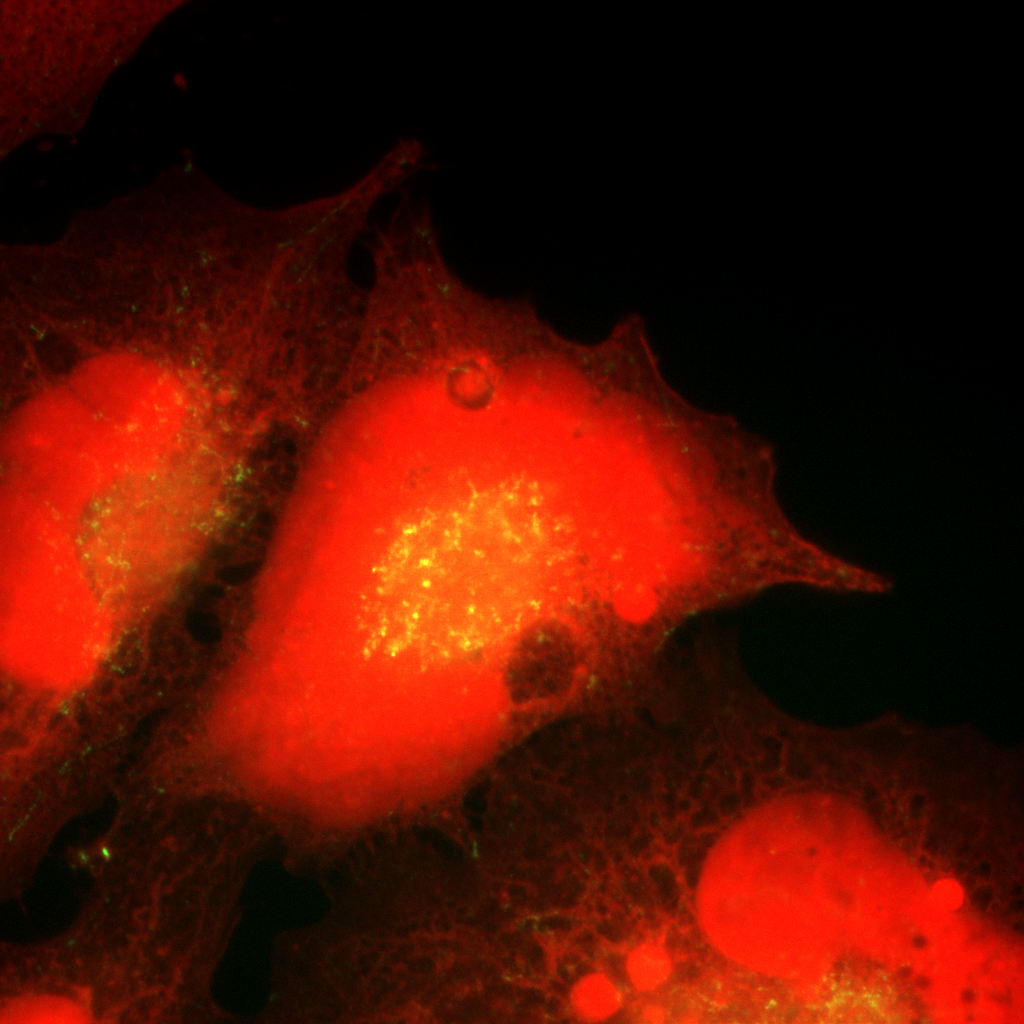

Supplement: S2 Dataset — Whole cell stain in red channel, endogenous SNX1 labelled in green channel. (ZIP) [file pone.0168294.s005.zip › Example Spastin depletion Images/Spastin knockdown 4.tif]

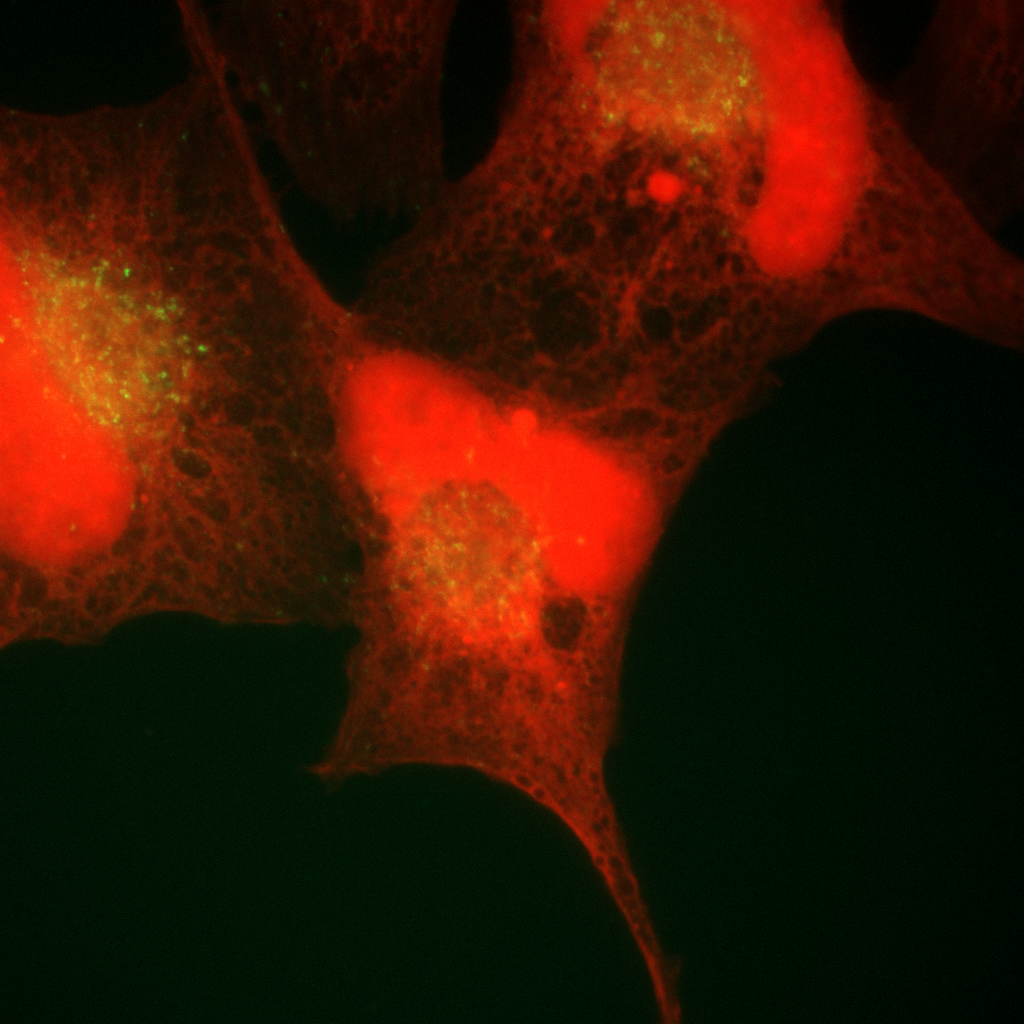

Supplement: S2 Dataset — Whole cell stain in red channel, endogenous SNX1 labelled in green channel. (ZIP) [file pone.0168294.s005.zip › Example Spastin depletion Images/Spastin knockdown 40.tif]

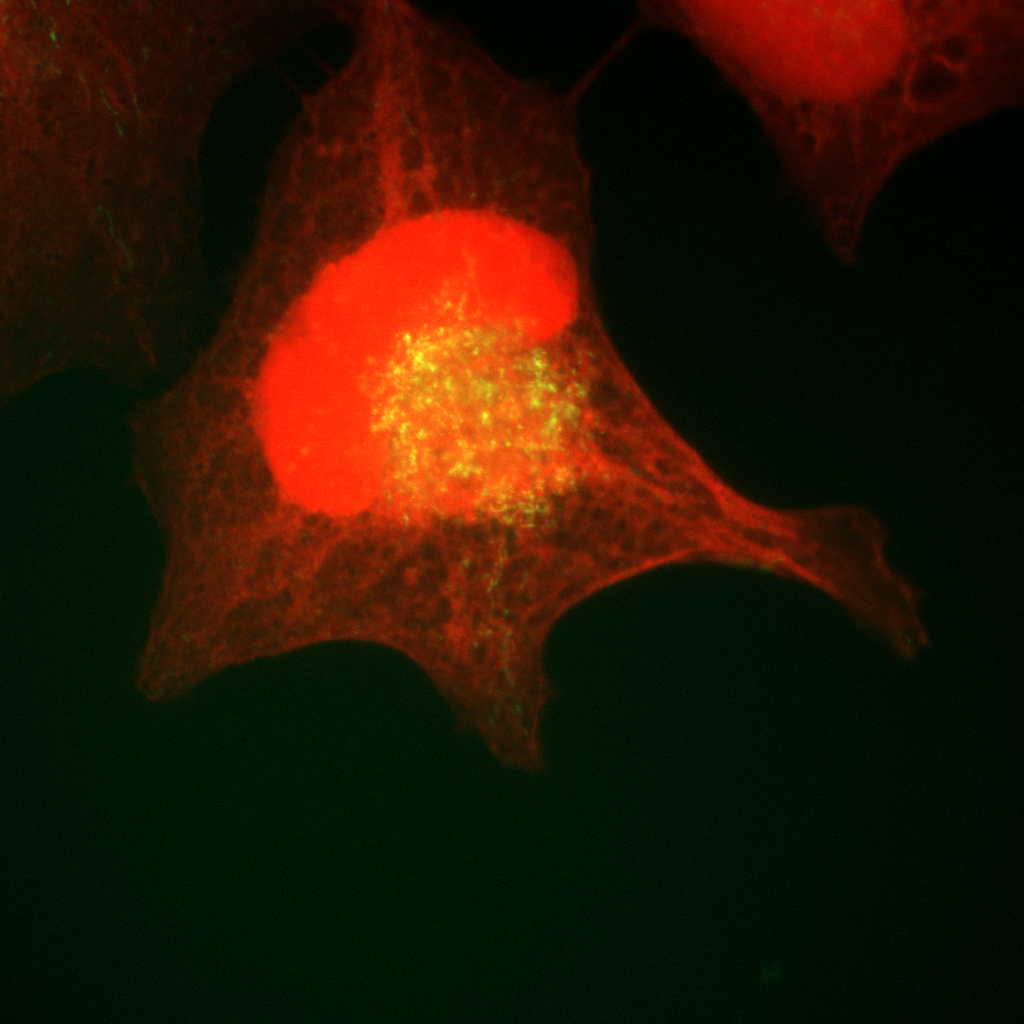

Supplement: S2 Dataset — Whole cell stain in red channel, endogenous SNX1 labelled in green channel. (ZIP) [file pone.0168294.s005.zip › Example Spastin depletion Images/Spastin knockdown 41.tif]

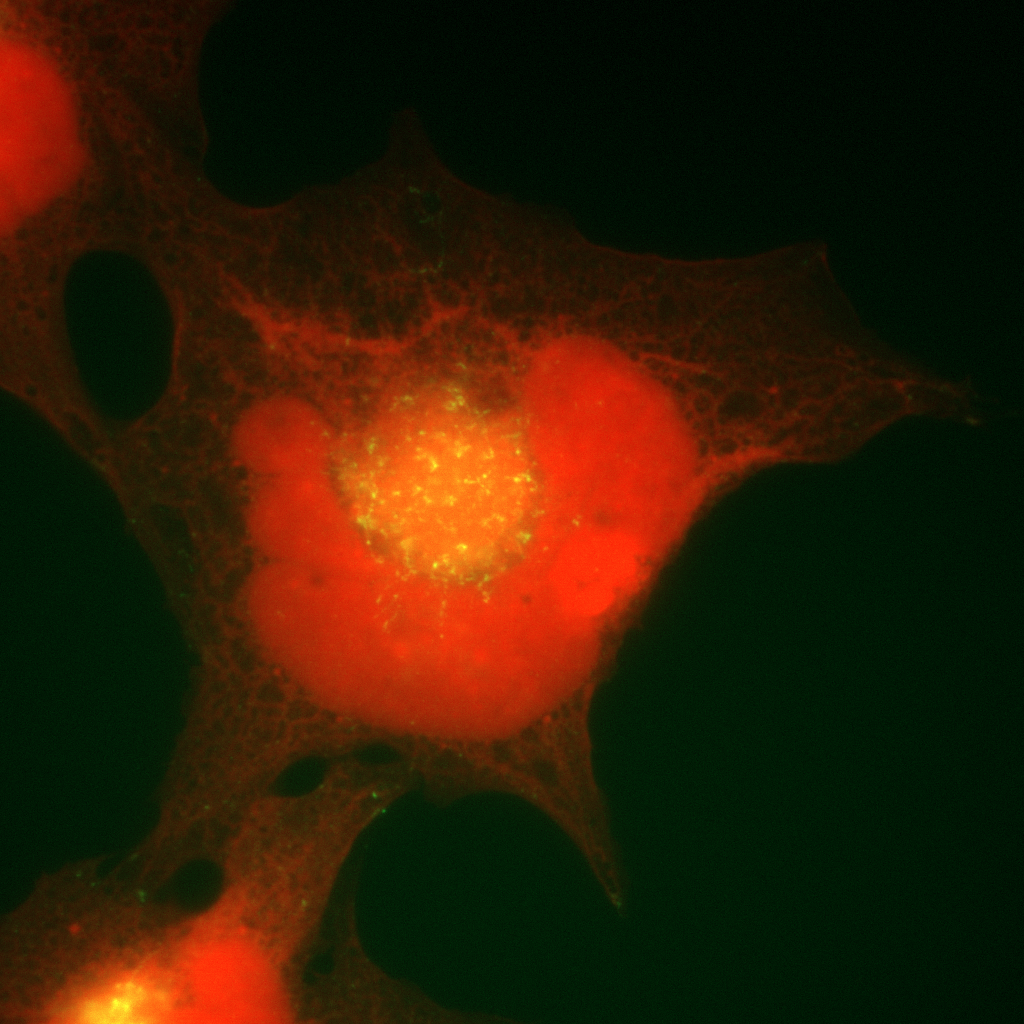

Supplement: S2 Dataset — Whole cell stain in red channel, endogenous SNX1 labelled in green channel. (ZIP) [file pone.0168294.s005.zip › Example Spastin depletion Images/Spastin knockdown 42.tif]

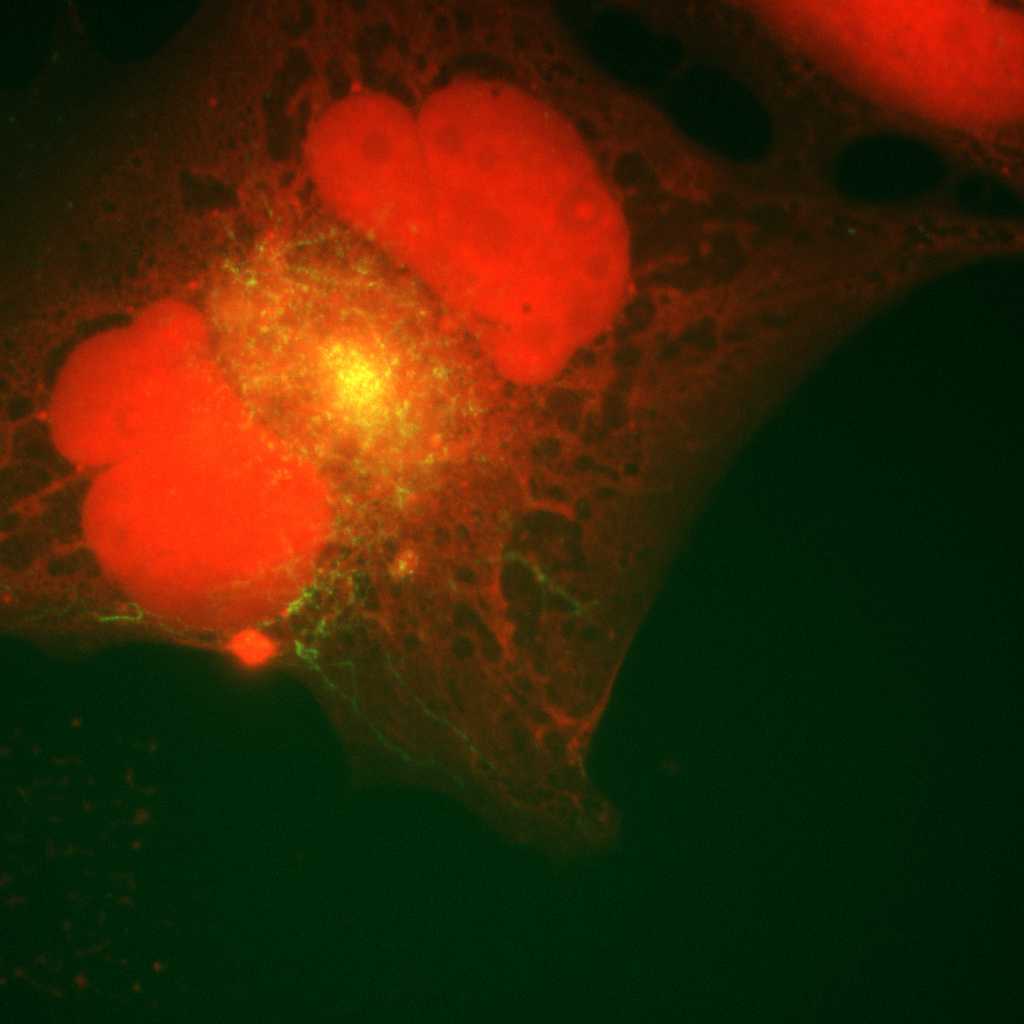

Supplement: S2 Dataset — Whole cell stain in red channel, endogenous SNX1 labelled in green channel. (ZIP) [file pone.0168294.s005.zip › Example Spastin depletion Images/Spastin knockdown 43.tif]

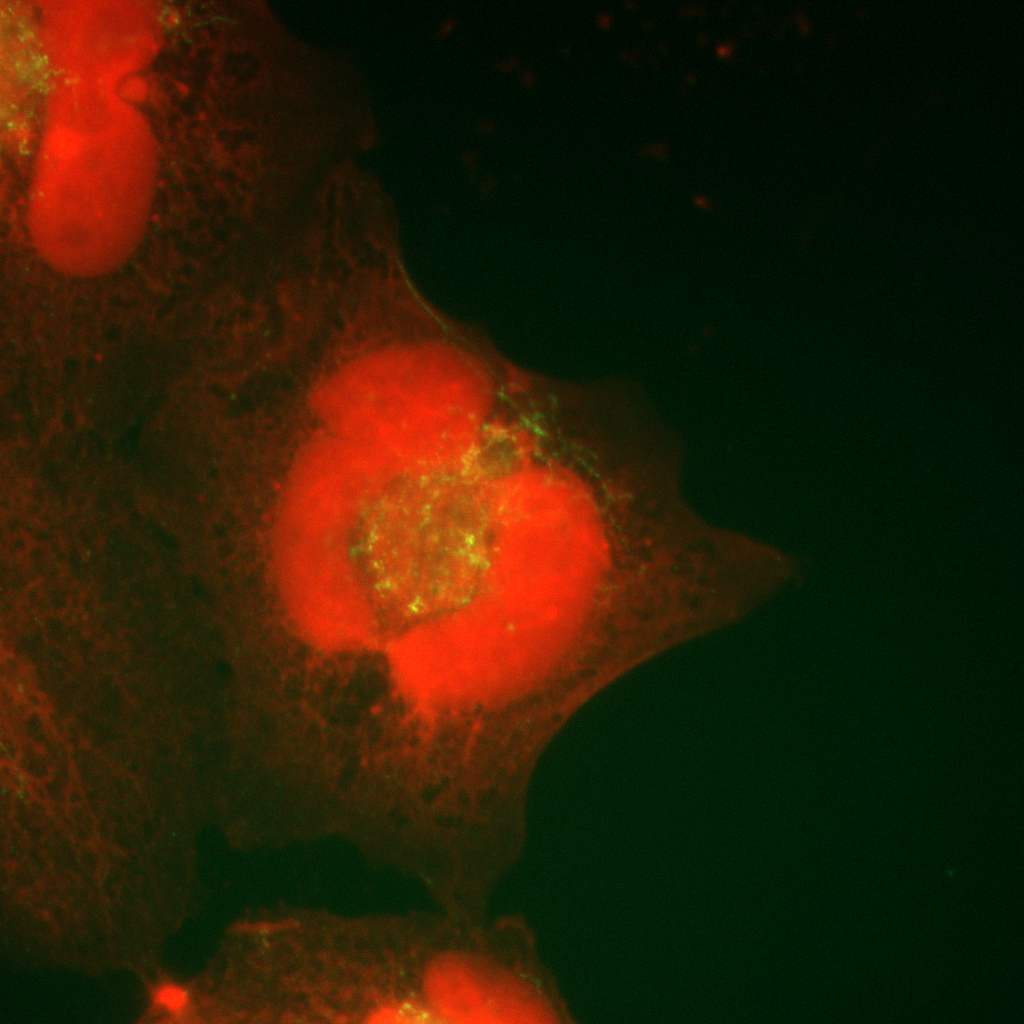

Supplement: S2 Dataset — Whole cell stain in red channel, endogenous SNX1 labelled in green channel. (ZIP) [file pone.0168294.s005.zip › Example Spastin depletion Images/Spastin knockdown 44.tif]

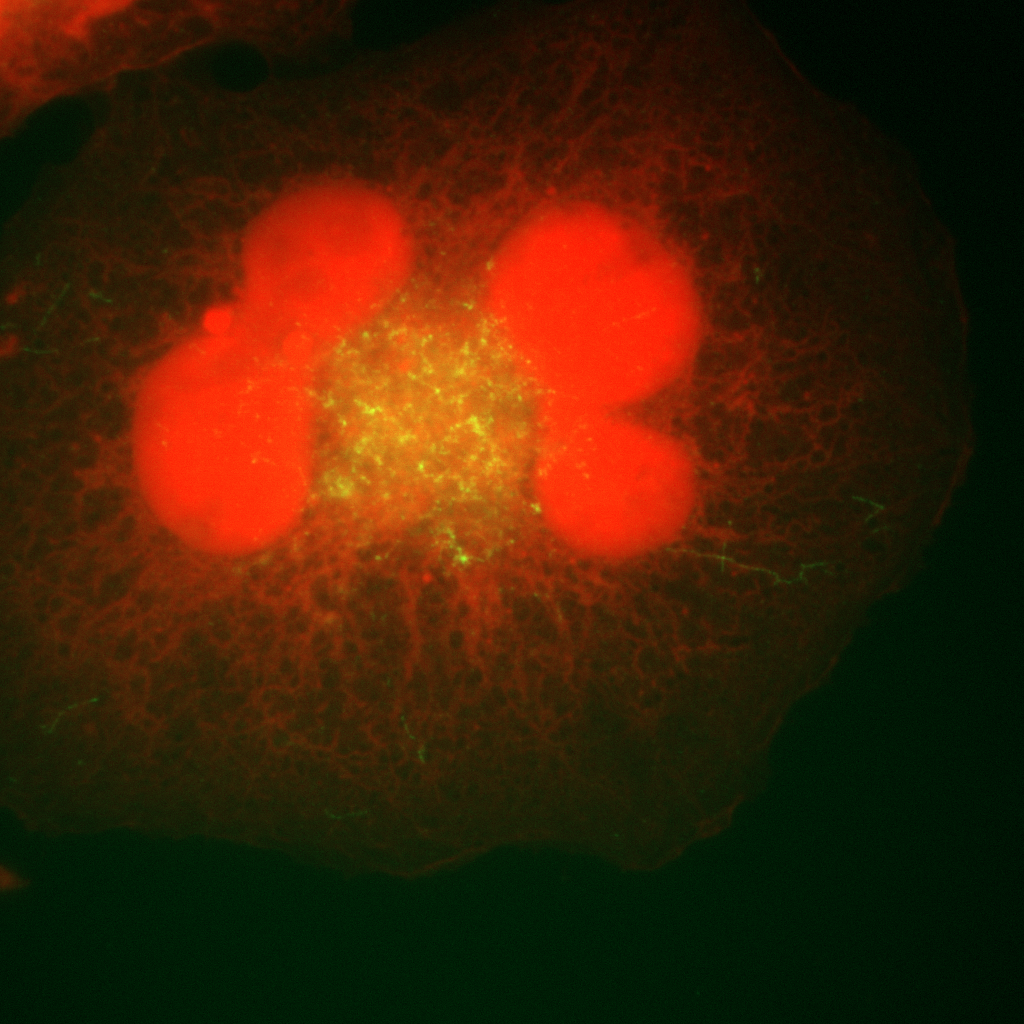

Supplement: S2 Dataset — Whole cell stain in red channel, endogenous SNX1 labelled in green channel. (ZIP) [file pone.0168294.s005.zip › Example Spastin depletion Images/Spastin knockdown 45.tif]

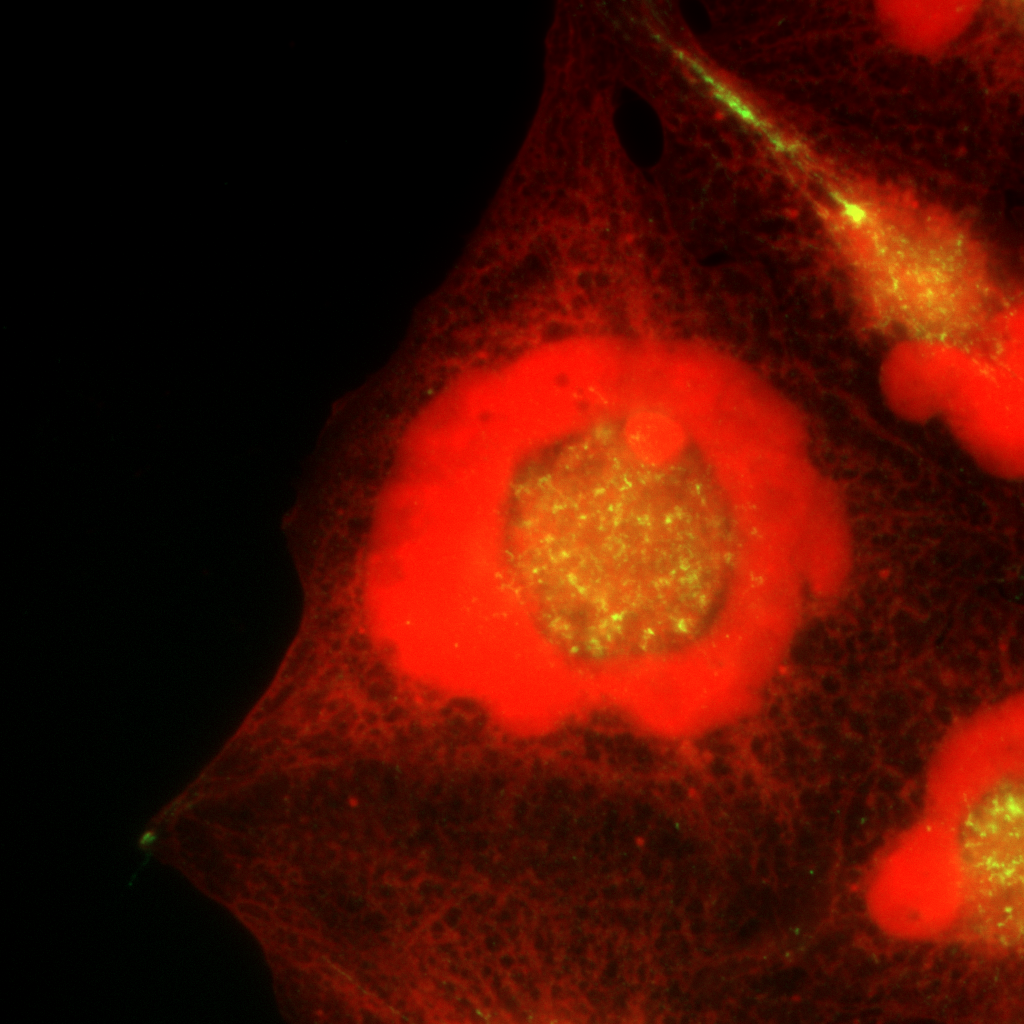

Supplement: S2 Dataset — Whole cell stain in red channel, endogenous SNX1 labelled in green channel. (ZIP) [file pone.0168294.s005.zip › Example Spastin depletion Images/Spastin knockdown 5.tif]

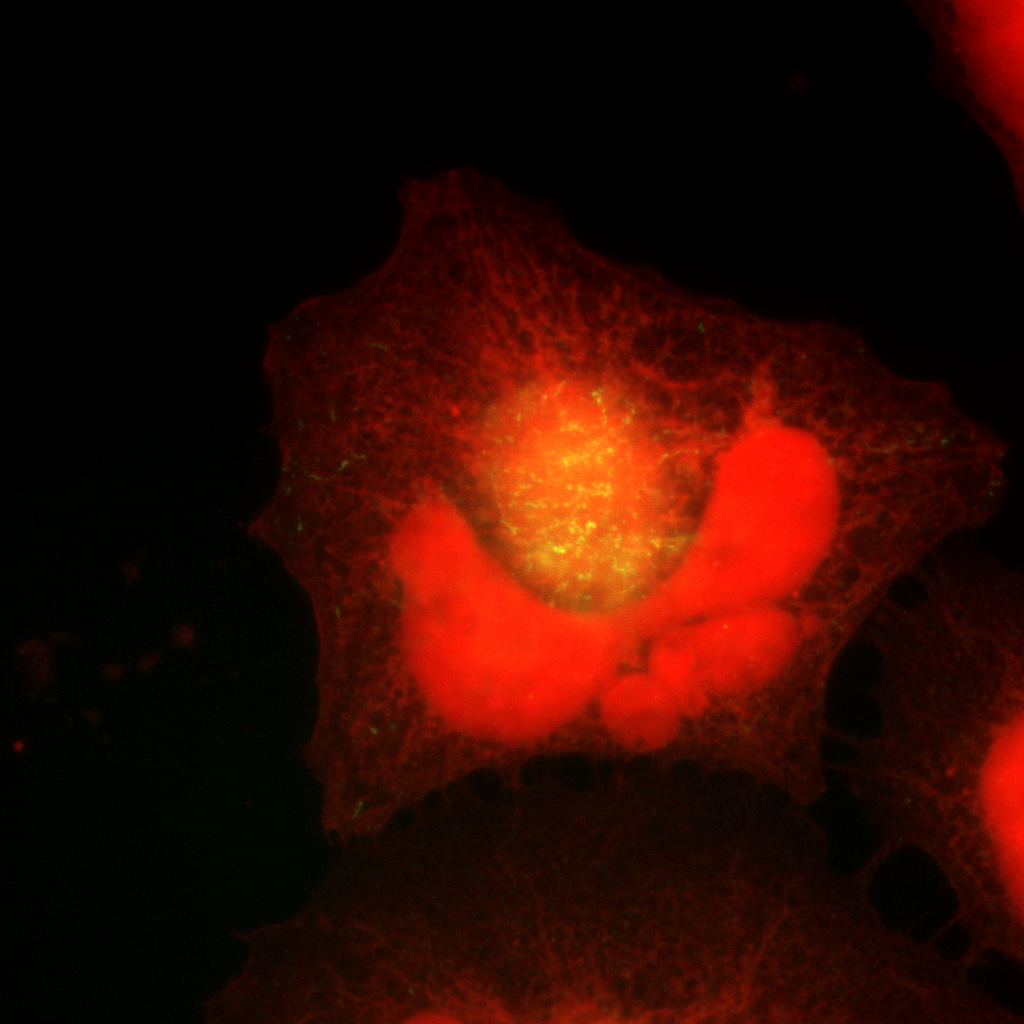

Supplement: S2 Dataset — Whole cell stain in red channel, endogenous SNX1 labelled in green channel. (ZIP) [file pone.0168294.s005.zip › Example Spastin depletion Images/Spastin knockdown 6.tif]

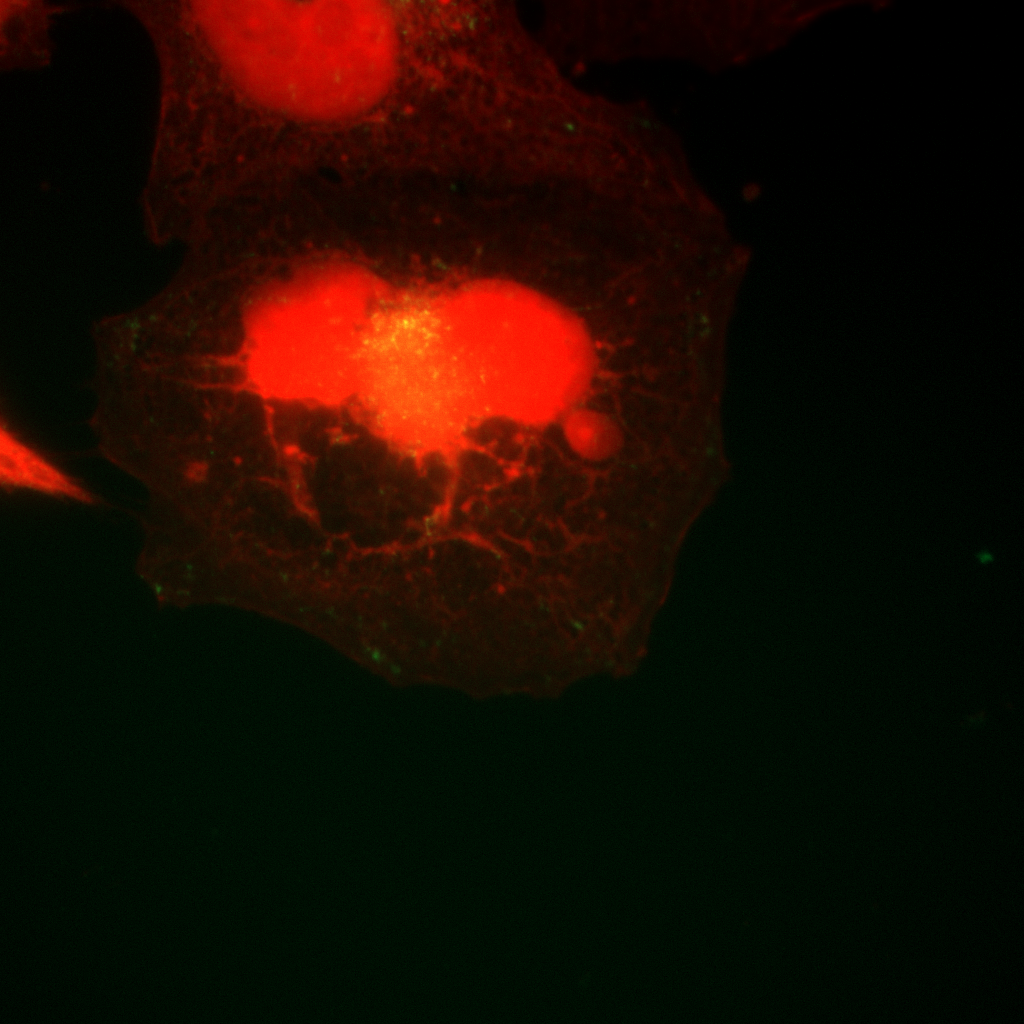

Supplement: S2 Dataset — Whole cell stain in red channel, endogenous SNX1 labelled in green channel. (ZIP) [file pone.0168294.s005.zip › Example Spastin depletion Images/Spastin knockdown 7.tif]

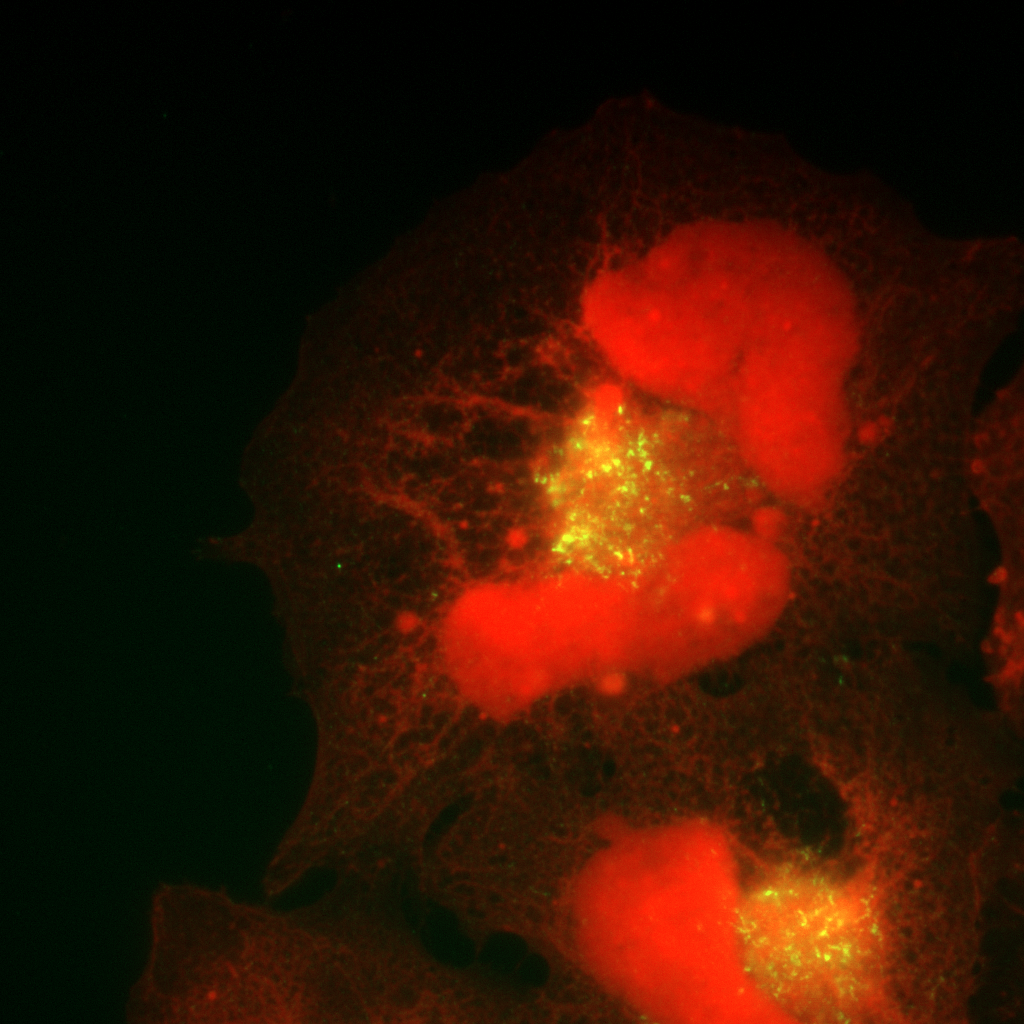

Supplement: S2 Dataset — Whole cell stain in red channel, endogenous SNX1 labelled in green channel. (ZIP) [file pone.0168294.s005.zip › Example Spastin depletion Images/Spastin knockdown 8.tif]

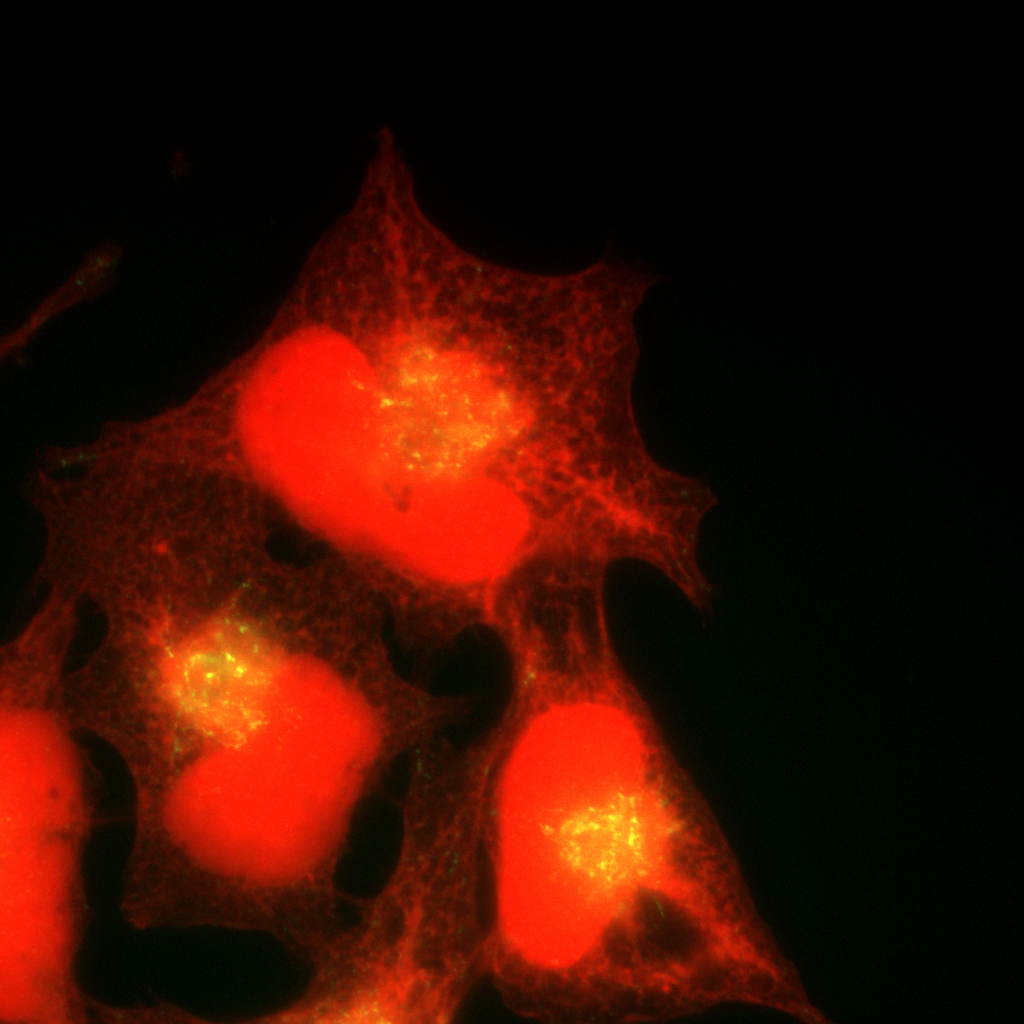

Supplement: S2 Dataset — Whole cell stain in red channel, endogenous SNX1 labelled in green channel. (ZIP) [file pone.0168294.s005.zip › Example Spastin depletion Images/Spastin knockdown 9.tif]
